# A minimalist model of extinction and range dynamics of virtual mountain species driven by warming temperatures

**DOI:** 10.1371/journal.pone.0213775

**Published:** 2019-03-18

**Authors:** Jonathan Giezendanner, Enrico Bertuzzo, Damiano Pasetto, Antoine Guisan, Andrea Rinaldo

**Affiliations:** 1 Laboratory of Ecohydrology, École Polytechnique Fédérale de Lausanne, 1015 Lausanne, Switzerland; 2 Department of Environmental Sciences, Informatics and Statistics, University of Venice Ca’ Foscari, 30123 Venezia Mestre, Italy; 3 Department of Ecology and Evolution, University of Lausanne, 1015 Lausanne, Switzerland; 4 Institute of Earth Surface Dynamics, University of Lausanne, 1015 Lausanne, Switzerland; 5 Dipartimento ICEA, Università di Padova, 35131 Padova, Italy; University of Molise, Isernia, ITALY

## Abstract

A longstanding question in ecology concerns the prediction of the fate of mountain species under climate change, where climatic and geomorphic factors but also endogenous species characteristics are jointly expected to control species distributions. A significant step forward would single out reliably landscape effects, given their constraining role and relative ease of theoretical manipulation. Here, we address population dynamics in ecosystems where the substrates for ecological interactions are mountain landscapes subject to climate warming. We use a minimalist model of metapopulation dynamics based on virtual species (i.e. a suitable assemblage of focus species) where dispersal processes interact with the spatial structure of the landscape. Climate warming is subsumed by an upward shift of species habitat altering the metapopulation capacity of the landscape and hence species viability. We find that the landscape structure is a powerful determinant of species survival, owing to the specific role of the predictably evolving connectivity of the various habitats. Range shifts and lags in tracking suitable habitat experienced by virtual species under warming conditions are singled out in different landscapes. The range of parameters is identified for which these virtual species (characterized by comparable viability thus restricting their possible fitnesses and niche widths) prove unable to cope with environmental change. The statistics of the proportion of species bound to survive is identified for each landscape, providing the temporal evolution of species range shifts and the related expected occupation patterns. A baseline dynamic model for predicting species fates in evolving habitats is thus provided.

## Introduction

Little dispute exists regarding the major impacts on biodiversity expected from climatic changes [1–4], but predictive studies blending landscape and population ecology still face serious challenges, especially when related to complex topographies [5–7]. As the rate of warming over the past 50 years (0.13±0.03 °C per decade [8]) is approximately twice that observed for the previous 50 years, extinction dynamics are likely to be challenged by evolving geophysical drivers almost everywhere [9], a special threat to mountain species owing to their high rate of local endemism [5, 10]. Shifts of geographic distributions may be rapid [11] and heterogeneous [12, 13], also in response to habitat fragmentation [14]. Species are facing two survival options: stay or go [7, 15], i.e. adapt to the new imposed local conditions or track the displaced suitable climate. Then, species that could potentially live in the new ecological landscapes created by climate warming may fail to track the displacements of their habitat and go extinct [16, 17], and those that persist, even if coping with the new conditions, might still be affected by extinction debts [18, 19].

By taking into account upward shifts of suitable species habitats [20], effects of climate warming would be reflected in the increase/decrease of suitable occupied sites along elevation gradients [17, 21] and the resulting displacements of species [3, 15]. Field evidence on changes in species lower and upper range limits, optima, and abundances [4] may thus be examined for specific landscapes. Whereas environmental drivers affecting species ranges are various and competing, at progressively larger spatial scales, air temperature, tightly linked to elevation, emerges as a key player [22, 23]. General frameworks in the literature frequently consider many other drivers. Some of them change predictably with elevation (for instance, anthropogenic pressure and, to a lesser extent, precipitation [24, 25]), while others are not elevation-dependent (such as moisture, clear-sky turbidity and cloudiness, sunshine exposure and aspect, wind strength and exposed lithology to name a few without specific taxa in mind [24, 26]). However, theoretical analyses aimed specifically at geomorphic factors are essential to inch towards the prediction of spatial biota responses [5, 27, 28].

The structure of the environmental matrix is known to affect biodiversity patterns [29–34]. Mountain landscapes provide a complex matrix, often shaped chiefly by fluvial erosion and geologic uplift [35], resulting in the majority of their surface to lie at intermediate elevations [6]. Owing to the ubiquitous fractal nature of their topographies [35], in such landscapes one may find peaks or troughs at the same elevation, resulting in different degrees of isolation and connectivity [36]. Warming temperatures would prompt species to experience alterations of the spatial configuration of their habitat, as well as an alteration of the microclimatic heterogeneity [7], because of the change in the area available at the elevation yielding optimal fitness, and hence of the proximity of areas with similar ecological characteristics (connectivity) and the dispersal ability [37], crucial determinants of species persistence [4, 6, 38]. This also implies that deriving species distribution patterns from linear elevational gradients, for simplistic geometric shapes with same relief, is ecologically useful mostly as a null model against which to compare patterns derived from real landscapes [5, 6].

Here, we use a metapopulation modelling framework [39], generalized by the incorporation of a specific fitness function describing how suitable the local landscape features are for a virtual species to thrive in (i.e. the quality of a cell [14]), similarly to what is done in habitat suitability models (HSMs) involving virtual species [40–42]. For the main purpose of this manuscript, which is to highlight the impact of different topographies on species occupancy and survival to climate warming, we make the minimalist assumption that fitness depends only on elevation. While one should be careful in using terminology like habitat when the models only consider elevation as an environmental layer, we justify our choice by a twofold argument: on the one hand, we keep ecological detail to a minimum to be capable of sorting out true landscape effects; on the other hand, extensions of our framework to include other environmental covariates, as typically done in HSMs [43] and other dynamical ecological models [44], is straightforward (see S4 File for an example directly incorporating additional landscape attributes), although outside the main scope of this paper. Landscapes are thus fully characterized by their elevation structure that defines the local species fitnesses and the processes governing species occupancy. By contrasting the occupancy results for a selection of landscapes, the influence of the elevational structure is studied.

The regional persistence of the virtual species studied here stems from balancing colonization and extinction processes [7] driven by local suitability specific to each landscape for an exactly identified range of species parameter values. In order to consistently investigate the geomorphological influence of different landscapes, these virtual species are characterized by comparable viability (i.e. the same metapopulation capacity [45]) in unbiased conditions, thus restricting their possible fitnesses and niche widths to specific parameter values. These virtual species have either large niche breadth but low fitness everywhere, or the opposite.

**Fig 1:**
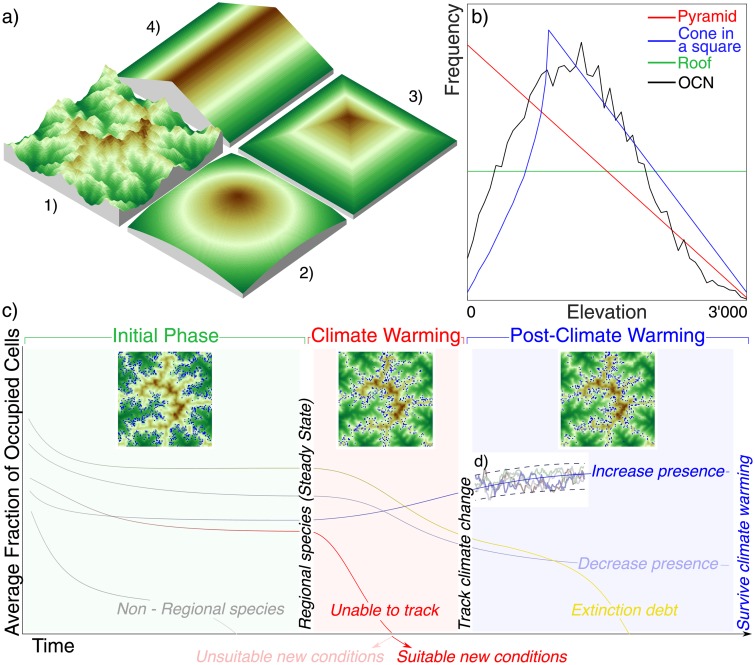
Landscapes used in the simulation and overview of the simulation. (a) The four synthetic landscapes used in the simulations meant to single out geomorphic effects: 1) a virtual realistic landscape based on the Optimal Chanel Network model (OCN, see S2 File), 2) a cone-in-a-square, 3) a pyramid and 4) a roof. (b) Hypsographic curves, defined as the area distribution at the various elevations. (c) A sketch of the three phases of the experiment: an initial phase (green) to select regional species; a climate warming phase (red) characterized by an upward shift in species optimal elevations, which discriminates between species able and unable to track climate warming; and post-climate warming phase (blue) exploring if the surviving species are suited to the new conditions, or experience extinction debt. The smooth lines depicted in the figure are meant as schematic representations of possible pathways of ensemble averages of several realizations (d) for a given species.

The overarching goal of this study is to systematically investigate how topography interplays with species parameters to concert their survival as a result of given climatic warmings (Fig 1). Owing to its deliberate simplicity and minimal parameter use, the metapopulation model is used extensively to explore the spatial occupancy of the various species. Even with such simple structure, enough degrees of freedom exist to produce realistic occupancy results.

## Materials and methods

### A metapopulation framework

A spatially explicit stochastic patch occupancy model (SPOM [14, 46]) is employed to simulate the distribution of a virtual species in a landscape. Building on a DEM with *N* cells, SPOM computes a possible distribution of occupied cells at every simulation time *t* by considering extinction and colonization processes, whose rates depend on the species properties and on the landscape features. A binary state variable *p*_*i*_(*t*) is set to 1 when the cell *i* is occupied and 0 when empty (*i* = 1, …, *N*). Starting from a given initial distribution of occupied cells, at each time step, the model allows unoccupied cells to be colonized by surrounding occupied cells with probability *P*_*C*,*i*_(*t* + Δ*t*) = *P*[*p*_*i*_(*t* + Δ*t*) = 1|*p*_*i*_(*t*) = 0]. Then, the cell becomes occupied at time *t* + Δ*t* depending on a random sample from a Bernoulli distribution with parameter *P*_*C*,*i*_(*t*). Similarly, species in occupied cells can go extinct with probability *P*_*E*,*i*_(*t* + Δ*t*) = *P*[*p*_*i*_(*t* + Δ*t*) = 0|*p*_*i*_(*t*) = 1]. SPOM works as a Markov chain, where, for each cell, the probabilities of colonization and extinction events are modelled with the following exponential distributions (and the probabilities of these events not happening as their respective complements):

$$\begin{array}{cc} & P_{C,i}\left(t+\Delta t\right)=P[p_{i}\left(t+\Delta t\right)=1\;|\;p_{i}\left(t\right)=0]=1-\exp\left(-C_{i}\left(t\right)\cdot \Delta t\right), \end{array}$$ (1a)

$$\begin{array}{cc} & P_{E,i}\left(t+\Delta t\right)=P[p_{i}\left(t+\Delta t\right)=0\;|\;p_{i}\left(t\right)=1]=1-\exp\left(-E_{i}\cdot \Delta t\right), \end{array}$$ (1b)

where Δ*t* is the simulation time step and *E*_*i*_ and *C*_*i*_(*t*) are the extinction and colonization rates (with dimension 1/*t*) for cell *i* at time *t*. Note that *C*_*i*_(*t*) is time-dependent because it depends on the current distribution of the species. The colonization and extinction mechanisms are directly related to a fitness function, *f*_*i*_ (described in the next section), which measures the suitability of the features of patch *i* for the species. The local extinction rate on a cell *i* is inversely proportional to the fitness, i.e., *E*_*i*_ = *e*/*f*_*i*_, where *e* is the extinction constant. The colonization rate of an unoccupied cell is driven by the sum of the contributions from surrounding occupied cells and is defined by a two-dimensional exponential kernel multiplied by the fitness associated with the source cells, i.e. the connectivity to the different cells as defined by metapopulation theory [39, 46–49]:

$$\begin{array}{c} C_{i}\left(t\right)=c\sum_{j\neq i}p_{j}\left(t\right)\frac{\exp(-d_{ij}/D)}{2\pi D^{2}}f_{j}, \end{array}$$ (2)

where *d*_*ij*_ is the distance between cells *i* and *j*, *D* the dispersal distance and *c* the colonization constant. Notice that connectivity is solely based on Euclidean distance and does not depend on the suitability of the path between cells.

### Fitness

We represent the species suitability at cell *i* having elevation *z*_*i*_ by the following fitness function *f*_*i*_ [6, 14]:

$$\begin{array}{c} f_{i}=f_{\max}\exp(-\frac{{\left(z_{i}-z_{\text{opt}}\right)}^{2}}{2\sigma^{2}}), \end{array}$$ (3)

where: *z*_opt_ describes the elevation where the species shows its maximal fitness; *σ* is the niche width, which sets how fast fitness decreases departing from *z*_opt_; and *f*_max_ is the maximum value of the fitness of the chosen pool of species. Once these species-specific parameters are assigned, the heterogeneity of the landscape matrix dictates the spatial distribution of fitness.

In the ensuing simulations, fitness is assumed to depend strictly on elevation. In another context, where geomorphic effects were not the main subject of inquiry, such a condition could be relaxed by considering other environmental covariates, making fitness more realistically dependent on habitat suitability (see e.g. [43]).

A number of approaches have used similar fitness functions [6, 14, 50]. For example, in studies of adaptation dynamics of spatially heterogeneous metapopulations, individuals have been assumed to be classified with respect to their phenotypes. Phenotypes are characterized by the value, or the strategy, *x*_*i*_ of a continuous trait *x* [50], where each habitat encountered in each patch determines the probability of survival of the phenotype, typically via Eq 3. Therein, differences between optimal traits (analog to *z*_opt_) for the different habitats generate tradeoffs and selective maladaptations.

### Comparable species viability

The overarching goal of this paper is to highlight the effects of geomorphology on species survival in the context of a minimalist metapopulation model. In order to single out these effects, we designed a framework where, for selected combinations of *f*_max_ and *σ* (Eq (3)), the considered species all display a comparable viability (i.e. metapopulation capacity *sensu* Hanski [45]). This is done in a geomorphologically unbiased environment, i.e. a landscape which does not favor selected species under mean-field assumptions (infinite dispersal). This ensures that a hypersurface in parameter space is postulated that contains only species with comparable viability, such that differences in species fate computed in various landscapes are directly related to different geomorphologies.

We define such geomorphologically unbiased environment moving from a 1D-landscape with a constant slope and infinite length where the metapopulation capacity is influenced by neither niche width nor optimal elevation. Within these assumptions, we center the landscape on the optimal elevation (i.e., *z*(*x* = 0) = *z*_opt_) and consider a finite domain of size [−*L*, *L*], with *L* large enough. The spatial discretization of the domain consists of *N* + 1 elements, *x*_0_ = −*L*, …, *x*_*N*/2_ = 0, …, *x*_*N*_ = *L*, with the distance between two points defined as Δ*x* (Δ*x* = 1 in this paper) and corresponding elevations *z_i_*(*x_i_*) = *x_i_* for *i* = 0, …, *N*.

The related metapopulation capacity, λ_*M*_, defines the theoretical threshold of the extinction to colonization ratio above which the population has no chance of survival (i.e., a species persists in the domain if and only if λ_*M*_ > *e*/*c*) [45], and is computed as the leading eigenvalue of the landscape matrix **M**, derived from the Jacobian of the system **J** = *c* **M** − *e* **I** [45, 51]. The matrix **M** contains information about the landscape and the quality of the patches. In the context of a geomorphologically unbiased landscape, and considering the mean-field theory, the elements of **M** are *m*_*ij*_ = *f*_*i*_ *f*_*j*_ if *i* ≠ *j* and *m*_*ij*_ = 0 if *i* = *j*. The values of the largest eigenvalue cannot be computed analytically. However, the Perron-Frobenius theorem provides an upper bound to the largest eigenvalue. Such upper bound is given as the maximum of the sums of the absolute value in a single row (or column) of the matrix. Considering the definition of **M**, the maximum of the row sum is obtained for the row corresponding to *z* = *z_opt_*, i.e. *i* = *N*/2:

$$\begin{array}{c} \lambda_{M}(f)\leq \sum_{j=0}^{N}f(z_{opt})f(z_{j})\Delta x, \end{array}$$ (4)

where *f* is the fitness function for a given species described in Eq (3). Letting *L* go to ∞ and reducing the size of the single elements to zero, the series in Eq (4) converges to the following integral:

$$\begin{array}{cc} & \int_{-\infty }^{\infty }f(z_{\text{opt}})f\left(z(x)\right)dx,\\ = & {\left(f_{\max}\right)}^{2}\int_{-\infty }^{\infty }\exp\left(-\frac{{\left(z(x)-z_{\text{opt}}\right)}^{2}}{2\sigma^{2}}\right)dx,\\ = & {\left(f_{\max}\right)}^{2}\sigma \sqrt{2\pi }, \end{array}$$ (5)

which, in order to constrain the largest eigenvalue of **M** to the same value for all niche widths, yields:

$$\begin{array}{c} f_{\max}=\frac{1}{\sqrt{\sigma }}\;,\;\forall \sigma . \end{array}$$ (6)

By incorporating this result in Eq 3, we obtain an ensemble of species having the same metapopulation capacity (i.e., same probability of surviving) in this theoretical domain. Thus, comparing virtual species having the proposed fitness function permits to understand the real structural effects of the landscapes, given initially unbiased species in the sense of survival ability.

### Climate warming

To simulate climate warming impacts, the optimal elevation of each species is gradually shifted. The IPCC reports different possible long-term greenhouse gas concentration trajectories, representative concentration pathways [9]. The worst-case scenario, RCP 8.5, predicts an increase in temperature in the range of Δ*T* = 4°C over the next century (Δ*t*_*c*_). This scenario is chosen in order to enhance as much as possible, yet not unrealistically, the impact of climate warming on the metapopulation model. We choose to uniformly change the optimal elevation of each species in the landscape: assuming a typical average global environmental lapse rate for air temperature of *γ*_*w*_ = 1/150°C/m [52], the optimal elevation thus changes at a speed of Δ*z*/Δ*t* = Δ*T*/(*γ*_w_ ⋅ Δ*t*_c_) = (4⋅150)/100 = 6 m/year, leading to a new optimal elevation $z_{opt}(t+\Delta t)=z_{opt}(t)+\left(\frac{\Delta T}{\gamma_{w}\Delta t_{c}}\right)\Delta t$ after each time step.

While the choice of lapse rate is justified based on rates of environmental change, empirical evidence suggests that many species, mostly plants [53] and birds [54], shift in elevation at slower paces than those assumed here for the drivers [55]. Note that lags can differ greatly in intensity for different altitudes [53]. Lags are obtained here by simulation and depend on the transient states given the imposed warming.

### Simulations

**Fig 2:**
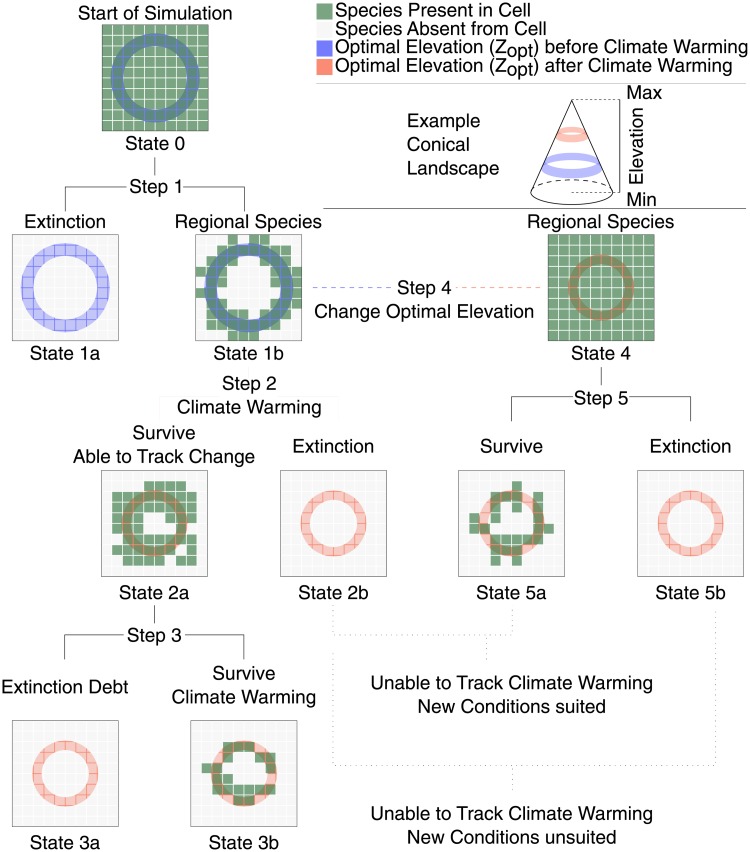
Overview of the different states and steps of the simulation. For simplicity the landscape is displayed as a cone. 100 random solution of the SPOM model are generated for all different combinations of parameters and landscapes. In Step 1 of each run, SPOM reaches an equilibrium occupancy starting from full occupancy (State 0). Species belonging to the regional pool, i.e. surviving Step 1, are utilized (State 1b) and climate warming is applied (Step 2) leading to survival (State 2a) or extinction (State 2b). Extinction debt is evaluated by computing the equilibrium condition for the species surviving to climate warming (Step 3). Additionally, new simulations are started (Steps 4-5) by computing their equilibrium occupancy starting from full occupancy and considering the optimal elevation after climate warming. This step identifies species unable to track climate warming but which would have been able to survive the new conditions (extinct suited), and species which went extinct with climate warming and for which these conditions would not have been suited anyway (loss of suitable habitat, extinct unsuited).

We generated a pool of 4000 virtual species with the parameter range for dispersal distances, niche widths and optimal elevations assumed such that they cover the scope of the regional species parameter, determined by running the first step of the simulation several times beforehand (*z*_opt_ ∈ [0, 3000], *σ* ∈ [25, 600] and *D* ∈ [0.1, 12]) for fixed values of *e* = 0.02 and *c* = 15 years^−1^. From this pool, a subset of virtual species, starting from a fully occupied landscape (i.e. each cell is occupied by the species, initial condition), persist in the landscape after a simulation is carried out until stationarity under constant climatic condition (Fig 2, step 1). Species belonging to this regional pool serve as initial condition for the evaluation of the effects of warming scenarios. Owing to the stochastic nature of SPOM, the persistence of a species is evaluated by repeating the simulation 100 times for each set of parameters (i.e., species) and landscape. Species that are still present in the landscape at the end of at least half of the 100 random runs are considered as regional species (Fig 2, Step 1).

In order to have 100 occupancy configurations for each of these species, runs leading to species disappearing from the landscape during step 1 are replaced by randomly selecting an equilibrium configuration from the remaining ones.

Starting from the occupancy of species obtained in step 1, we apply climate warming (step 2) as an upward shift of the niche for approximately 100 years.

Step 2 identifies species unable to track climate warming, i.e. thus initial species that go extinct during climate warming. Climatic conditions are then frozen and the experiment continues until the extinction debt has been paid off (step 3) which identifies the species unable to cope with the new temperatures (i.e. species affected by extinction debt [18]).

The last two steps (steps 4-5) consist in understanding whether the species which went extinct during the climate warming phase would be able to survive given their new optimal elevation (extinct suited), or if they would have gone extinct anyway due to loss of suitable habitat (extinct unsuited). In step 4, species are allowed once again to fully occupy the landscape, but their optimal elevations are shifted to the value after climate warming. The simulation is then run until reaching a steady state (step 5), allowing us to find the species surviving in the new conditions, and by comparison with state 2b, identifying the fate extinct suited/unsuited.

This method underpins observations of species transient states, and therefore singles out specific fates. Such fates can be the disappearance of species suitable to post-climate warming conditions due to their inability to track favourable conditions, or species survival for some time after climate warming, only to go extinct later. Note that the induced understanding of transient effects distinguishes metapopulation studies from habitat suitability and species distribution models (SDMs) [43, 56]. Note also that dynamical models sitting on top of SDMs [57] in order to capture the transient states and take advantage of the powerful SDMs tools exist.

**Fig 3:**
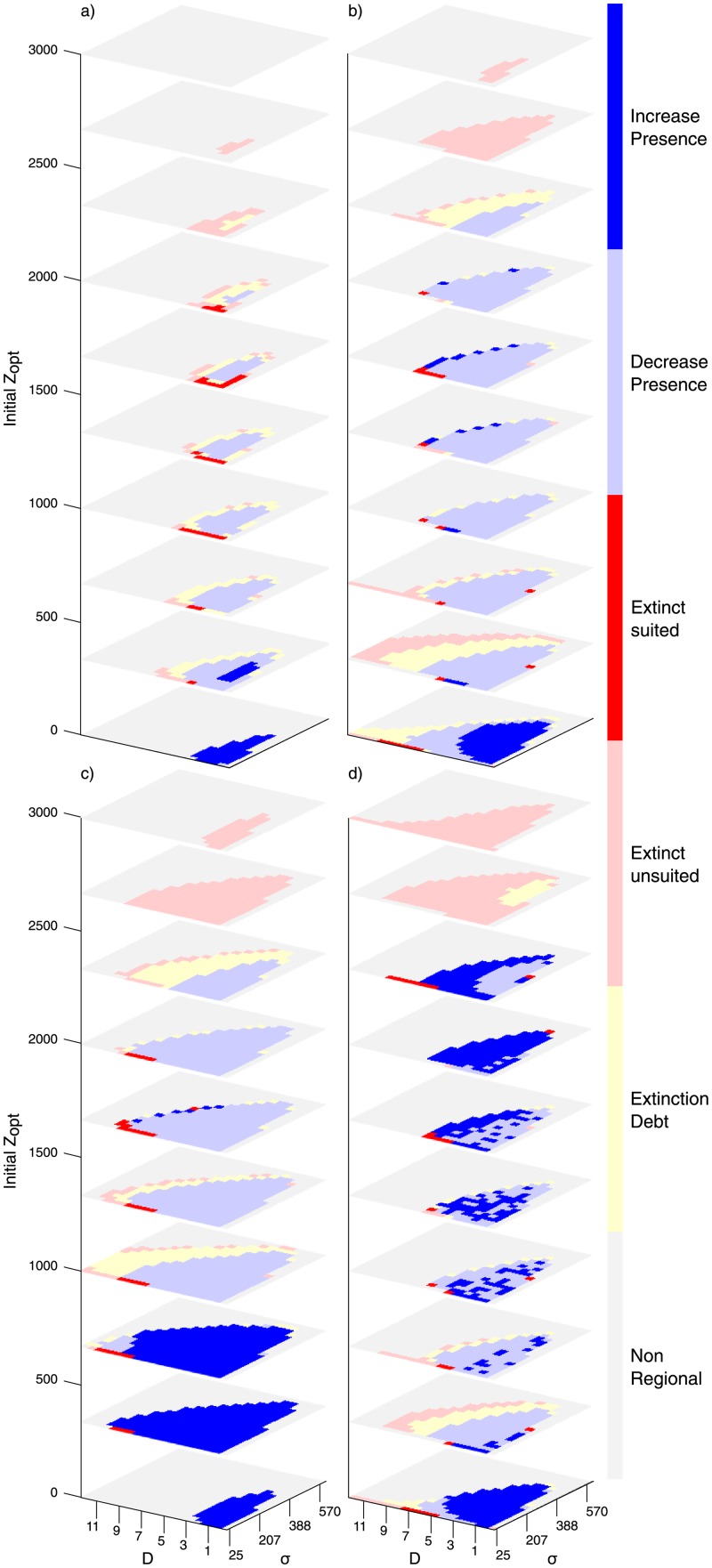
Outcome of the metapopulation runs depends on the initial optimal elevation *z*_opt_, niche width *σ* and dispersal distance *D*. Colors represent the fate of the virtual species (same color-code as in Fig 1 with detailed explanation of the fates in Fig 2), which is determined by the most probable outcome after 100 random model runs. Results are presented for the following landscapes: (a) OCN; (b) pyramid; (c) cone in a square; and (d) roof-like landscape. The range of parameters shown has been chosen to contain the areas where significant change is detected. See S3 File for the complete set of results.

The coefficients of extinction *e* and colonization *c* have been chosen such that all the possible species fates are observed in the simulations. Other choice would have been possible because, as long as the ratio between the coefficients stays constant, the outcome of the simulation remains the same (for small enough values of *dt* [45]). Other coefficient values would have generate a different pool of regional species, but the landscape occupancy would have been preserved (c.f. Fig 3 in the results section).

### Landscapes used in the simulation

We consider three different types of landscapes: synthetic (roof, cone-in-a-square and pyramid), realistic synthetic (Optimal Chanel Networks (OCNs)) and real (the Gran Paradiso National Park—GPNP, Italy and the Vaud Alpes, Switzerland). OCNs, synthetic landscapes with periodic boundary conditions previously used for ecological applications in the context of climate warming [58], are obtained by a metaheuristic approach by looking for a local minimum of total energy dissipation by iterating over different configurations of drainage directions corresponding to different topographic slopes [35, 59–61] (S2 File). The DEM of the real landscapes (GPNP and Vaud) are extracted from the online earth explorer tool, courtesy of the NASA EOSDIS Land Processes Distributed Active Archive Center (LP DAAC), USGS/Earth Resources Observation and Science (EROS) Center, Sioux Falls, South Dakota (https://earthexplorer.usgs.gov/). The results are computed on the administrative limits of GPNP and Vaud Ales, while the simulations are performed over a square with a buffer around the area to avoid border effects. The elevation fields are rescaled such that the relief would match to the synthetic landscapes (0-3000 meters).

These different landscape shapes are designed to probe boundary and structural geomorphic effects. For instance, the typical hump-shaped distribution of hypsographic curves (defined as the area distribution at the various elevations) found in nature [5, 6] (GPNP, Vaud Alpes) can be constructed in unrealistic geometries (Fig 1b, cone in a square) and realistic synthetic landscapes (OCN). The comparison among the different occupancies provided by the metapopulation model for a virtual species on these different landscapes highlights the influences of the spatial complexity of the topography on the habitat connectivity and fragmentation (e.g. comparing cone and OCN) and the possible impact of boundary problems (e.g. comparing OCN and real landscapes). Geometric landscapes are further used to understand how relief boundaries interact with niche width (comparing pyramid and roof), and how connectivity changes with constant hypsographic curves, as in the case of the roof. Comparative studies on real topographies and OCN landscapes are employed to highlight the influence of steep slopes characteristic of real landscapes, which are not present in OCNs where fluvial erosion is the dominant factor.

## Results

### Initial occupancy patterns

**Fig 4:**
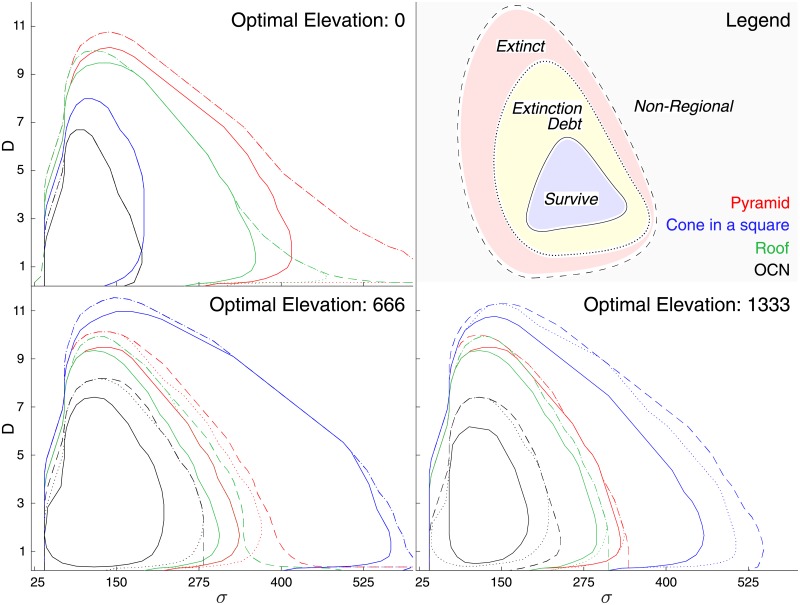
Progression of the parameter limits during the simulation. Parameter limits (niche width *σ*, dispersal *D*) of the initial pool of regional virtual species (dashed), species present after climate change (dotted) and species present at steady state after climate change (line). The figures show species with initial optimal elevations (*z*_opt_) of 0, 666 and 1333 m.

We analyze how landscape features control the persistence of species with specific parameter values. In all landscapes, and for all the optimal elevations characterizing the different species, only a subset of the considered species persisted after the initial phase with constant climatic conditions (regional species, color-coded in Fig 3). The various landscape shapes have differing presence patterns even if endowed with similar elevation distributions, like the OCNs and the cone in a square (Figs 1 and 4). The OCNs showed a limited region of parameter constituting the regional pool of species, suggesting an important effect of spatial aggregation of suitable habitat/landscape fragmentation (Fig 3).

### Climate change effects

In all considered landscapes, a subset of the parameters leads to the extinction of the species after climate change (Fig 3 (red) and Fig 4 (dashed line)). Results show that extinction debt is frequent for large niche widths and dispersal distances (Fig 3 (yellow) and Fig 4 (dotted line)). Part of the regional pool of species having an initial optimal elevation facing a rising hypsographic curve, that is, an increase of area of suitable habitat resulting from the upslope shift, i.e. when *z*_opt_ is located below the position of the peak (species in dark blue in Fig 3a and 3c) experienced an increase in their occupancy and range, whereas species ending up around peaks in the landscape after climate change, or when *z*_opt_ is located above the peak in the hypsometric curve, were unequivocally affected by a strong occupancy reduction and range reduction (all colors except dark blue in Fig 3a and 3c for *z*_opt_ > (500/1000) m). Thus, for a hump-shaped hypsographic curve, where the majority of land lies at intermediate elevations (OCNs, cones, real landscapes), the fate of a species depends on the relative position of the peak elevation compared to its initial *z*_opt_.

Extinction due to inability to track change has many causes. Species unsuited to new conditions are found where suitable habitat shrinks or disappears (loss of suitable habitat). Interestingly, however, in real landscapes and OCN replicas, species with small niche width are confronted with this likely outcome even if they gain habitat area or connectivity [39] with climate change. Species unable to track, but that would have been capable of surviving under altered conditions, i.e., lagging behind the rate of climate change [53, 55] (extinct suited, Fig 3, dark red), were found only for a small number of parameter sets, mostly for species with small niche width and relatively large dispersal values, and almost exclusively on species with optimal elevations situated in the decreasing part of the hypsographic curves. Species surviving climate warming (Fig 3, blue, Fig 4, straight line) were found to adjust their occupancy compared to their initial states. Species with a decreasing presence (Fig 3, light blue) were found when suitable area and/or proximity/connectivity decreased or when their niche ranges exceeded the maximum elevations. This pattern was commonplace for the pyramid where area monotonically decreases with elevation (Fig 1b).

### Extension to real landscapes

**Fig 5:**
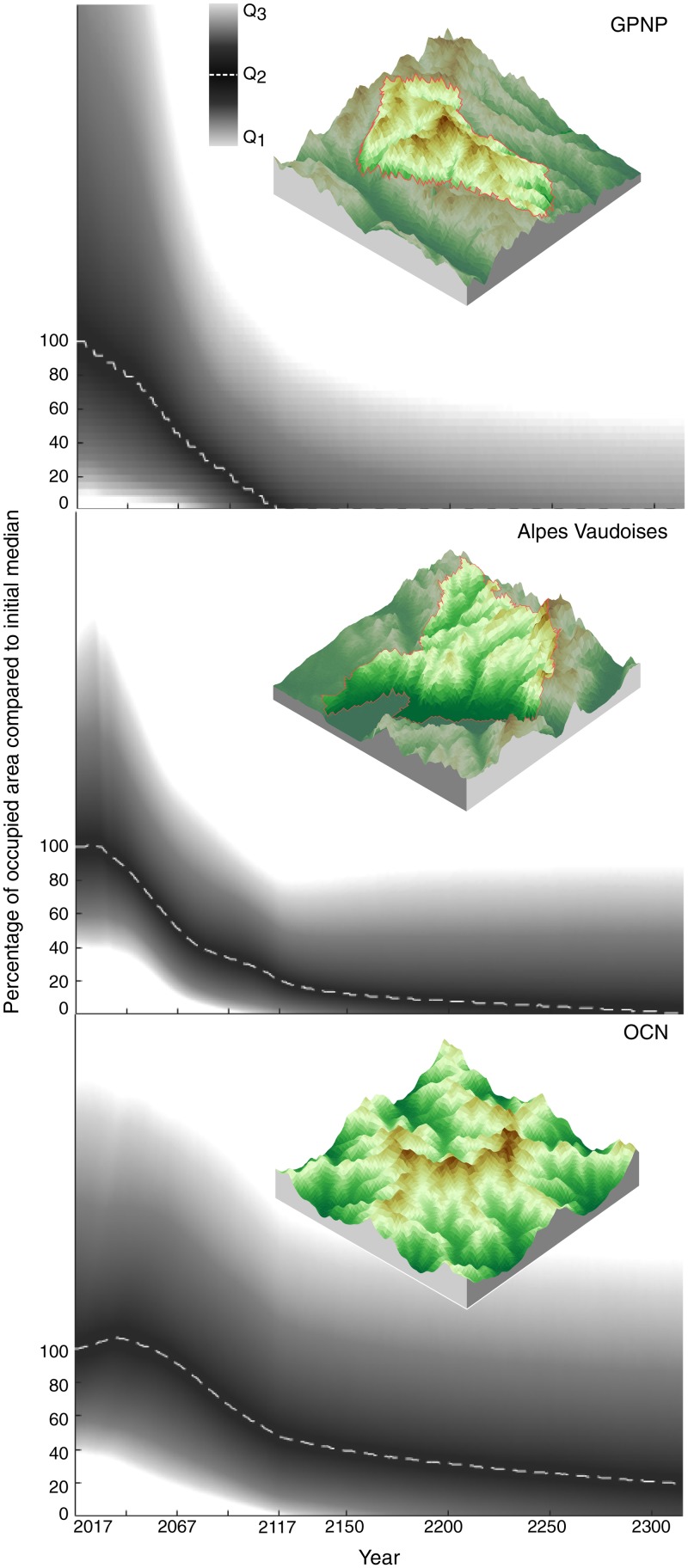
Species occupancy during (2017-2117) and after climate warming (after 2117) relative to the initial median occupancy. The dashed line represents the median percentage of occupied area by regional virtual species that decreases during and after climate warming. The red lines in the DEMs represent the limits where area was considered.

We analyzed real landscapes using DEMs for GPNP and Vaud Alps (Fig 5). We looked at how the geographic range of a pool of regional species shifts in response to climate warming. Fig 5 shows the temporal changes in the percentage of occupied area by a species compared to the initial median occupation. We do not consider in this analysis species that can immigrate from outside the system (e.g., from lower elevations) but focus only on the fates of regional species. As expected, occupancy decreased during climate warming. Interestingly, a long period is required to stabilize the occupancy after the imposed change. Species occupancy continued to decrease after the climate stabilized, and the median occupancy tended to zero in GPNP and Vaud Alpes. Owing to its geomorphological attributes, the GPNP region, which has the majority of land close to the maximum elevation, suffered the most from the loss in occupancy.

## Discussion

Given our parameter set, three intertwined factors govern the fate of the species at any site in a landscape: the initial occupancy distribution, the distance at each site from areas sharing similar fitness, and the available area around the optimal elevation, the main niche factor. Taken separately, we find that their effects do not suffice in predicting the fate of a species under climate warming. For example, the hypsographic curve alone does not subsume all processes governing species presence. Even in the simple, unrealistic geometric landscapes designed to distinguish geomorphic effects, the entanglement of those processes generates unforeseen outcomes (Figs 3 and 4). For instance, species endowed with their niche partially outside the landscape elevation range before climate warming, may actually exploit increased geographic range projection even if the specific hypsographic curve is steadily decreasing (Fig 1b, see also S3 File). Occupancy thus reflects a complex balance between area availability, connectivity and realized niche (see also [43] for an analogous effect captured by habitat suitability models). One may note that connectivity does not refer to dispersal alone. In fact, it typically incorporates some measures of land cover, land use, or landscape ‘permeability’ and is considered more a feature of the landscape rather than simply of the species ecology (like the dispersal distance) [5].

Our results suggest that equally viable species endowed with very large niche widths *σ* successfully track climate warming but often do not survive afterwards. In fact, under the imposed constraint on species parameters, such species have a relatively low fitness everywhere, which only allows survival if enough surface is colonized. Strong colonization is thus required to grant species survival relative to large dispersal distances. Otherwise, colonization will not compensate the low fitness and most of such areas—even if large—will slowly become unsuitable, leading to extinction debts. This particularly affects strong dispersers, whereas weak dispersers are more subject to landscape fragmentation because they rely on close-by areas to persist. Species with smaller niche and higher fitness are less affected by extinction debts, but may go extinct before the end of the imposed temperature rise, i.e., they might either track climate warming, and thus thrive given the new conditions, or go extinct during the process despite being suitable to the new conditions because of their isolation and the lag in tracking the new conditions. For such species, the local conditions are of utmost importance, because the fitness in a single patch suffices to make them survive without help from surrounding occupied cells (reduced available area), which causes them to be particularly sensitive to changes in available, close-by suitable habitats, and fragmentation (lack of short distance escape opportunities [53]). Such species are highly sensitive to microclimatic heterogeneity [7].

Realistic landscape heterogeneities are found to strongly impact species survival (Fig 4). The domain of parameters describing surviving species proves much smaller than that obtained for simple geometries. The defining role of connectivity is confirmed by noting that OCNs and the cone-in-a-square construct exhibit a rather similar hypsographic curve (Fig 1b) but rather different species fates (Fig 3). Local effects, such as mid-elevation plateaus or isolated peaks, influence the spatial projection of the niche, the geographical range and the proximity of similar areas (as in [62]), thus locally reducing the fitness of the species. If a landscape has a self-affine structure like many mountain ranges [35], highly fragmented connectivity is generated whereby species with larger niches but lower fitness struggle to subsist due to the weaker mutual rescue effect of occupied cells owing to meta-population processes of patch colonization-extinction. The differences between real and model landscape results are explained by the fact that OCNs imply large drainage densities (small ratios of the total river length to the catchment area normalized by the landscape characteristic size) and thus flatter slopes than in the real mountain landscapes studied here.

Certain assumptions of the minimalist model may be relaxed. They concern:

- the lack of incorporation of actual habitat suitabilities, here subsumed by the model Eq (3) chosen to strengthen the signals due to landscape effects. Although generalizable, such assumptions prevent a detailed ecological study of the species surviving the range shift beyond broad-brush statistics of their survival—however useful;
- the choice of characterizing climate warming as uniform and driven only by air temperature. Such assumption may be relaxed by using suitable climate and weather generators of various origins. This, however, would come at the risk of clouding the geomorphic effects highlighted in this work;
- the lack of heterogeneity of range shifts at the landscape scale [12, 13], here modeled by a simple upslope shift;
- the parameter choices describing local fitnesses, which is related to the constraints placed to maximal fitness (*f*_max_) to approximately conserve metapopulation capacity (Methods). This assumption reduces the number of species analyzed and yields a comparison in that similar viability of the species is assumed. Such assumption, like for instance the introduction of super-species with large width and high fitness that would colonize any landscape at every elevation, could be be introduced for specific studies at no change in the procedure (see S1 File for an example of a simulation with such a species);
- the neglect of invasions of species from lower elevations, unrepresented here. While we stand by our choice in view of the scopes of this paper, we note that neglecting them prevents a specific ecological study of how generalist or specialist species fare under the same circumstances in a given landscape. To that end, forthcoming metapopulation studies will be based on field evidence, possibly using generalized fitnesses that include all relevant covariates. S4 File gives more details about the effects of the factors we currently neglect in the mainstream discussion, and about technicalities on possible generalizations.

Moreover, long term evolution of populations can lead to local adaptation to environmental conditions (see e.g. [50] for a dynamic study based on spatially heterogeneous metapopulations). However, in the minimalist model pursued herein we assume that ecological timescales are much smaller than evolutionary ones. Phenotipic evolution of spatial metapopulations could instead be a possible development of the present approach, where heterogeneities might be provided by habitat suitability models [56] tailored to mountain ecosystems.

We nonetheless suggest, from the bulk of our extensive calculations, that strong influences are waged by broad geomorphic features of a landscape not only for biodiversity in equilibrium with the current climate [5, 6], but also for the long-term impact of climate warming on species survival. This is likely to be true in general, but especially so locally for heterogeneous landscapes. The extinction debt [17–19], which is hardly measurable as an ongoing process, is found to be a dominant feature in species dynamics according to our modeling approach for any realistic landscape. This feature would make it difficult (if not impossible) to predict changes before they actually happen without models. Especially alarming seems the suggestion that the effects of warming would not be limited to phasing, but would rather be also characterized by the disappearance of large amounts of occupied areas long time after the actual change in the driver. The timescales of the ecological response triggered by the imposed geographic range shift, in fact, are suggested to be much longer than that of the phasing itself, as extinction debts may be operating even centuries later.

## Conclusion

Our main conclusions can be summarized as follows.

Computational studies on the effects of climate warming prove essential to sort out genuine landscape effects on metapopulation range dynamics and spatial occupation of species under climate warming, not simply in terms of stationary states but also of transients defining the range of possible extinction timescales. Geomorphic effects on species survival can be sorted out because a climate warming scenario was applied to a regional pool of species previously filtered for the initial temperature regime under the discriminating (and defining) requirement of equal ecological viability (i.e. same metapopulation capacity). This results in a specific constraint on the virtual species’ fitnesses sensitive to the geomorphic effects sought after.

Minimalist models of the type explored here allow us to quantitatively show how extinction debts unfold. This is due to their computational ease that allows extensive search in the space of species parameters.

Future work will exploit the proposed framework to include other dimensions of habitat suitability in generalized fitness models. This may be done by correlating fitness to a number of environmental covariates (e.g., precipitation, soil type, aspect (S4 File), resources, land-use, etc.) rather than simply to elevation, or allowing metapopulation capacity to vary, i.e., incorporating species with both small/large niche breadth/fitness.

## Supporting information

S1 File Species parameters trade-off. (PDF)

S2 File Building statistically identical replicas of mountain topographies. (PDF)

S3 File Computational experiment. (PDF)

S4 File Generalized fitness model. (PDF)

## References

[1] Parmesan C, Yohe G. A globally coherent fingerprint of climate change impacts across natural systems. Nature. 2003;421:37–42. 10.1038/nature01286 12511946

[2] Parmesan C. Ecological and evolutionary responses to recent climate change. Annual Review of Ecology, Evolution, and Systematics. 2006;15:365–377.

[3] Lenoir J, Svenning JC. Climate-related range shifts—a global multidimensional synthesis and new research directions. Ecography. 2015;38(1):15–28. 10.1111/ecog.00967

[4] Rumpf SB, Hülber K, Klonner G, Moser D, Schütz M, Wessely J, et al Range dynamics of mountain plants decrease with elevation. Proceedings of the National Academy of Sciences of the United States of America. 2018;115:1–6. 10.1073/pnas.1713936115 PMC5828587 29378939

[5] Elsen PR, Tingley MW. Global mountain topography and the fate of montane species under climate change. Nature Climate Change. 2015;5:5–10. 10.1038/nclimate2656

[6] Bertuzzo E, Carrara F, Mari L, Altermatt F, Rodriguez-Iturbe I, Rinaldo A. Geomorphic controls on elevational gradients of species richness. Proceedings of the National Academy of Sciences of the United States of America. 2016;113(7):1737–1742. 10.1073/pnas.1518922113 26831107 PMC4763792

[7] Graae BJ, Vandvik V, Armbruster WS, Eiserhardt WL, Svenning JC, Hylander K, et al Stay or go—how topographic complexity influences alpine plant population and community responses to climate change. Perspectives in Plant Ecology, Evolution and Systematics. 2017;30:41–50. 10.1016/j.ppees.2017.09.008

[8] IPCC. Climate Change 2013: The Physical Science Basis. Stocker TF, Qin D, Plattner GK, Tignor M, Allen SK, Boschung J, et al, editors. Cambridge, United Kingdom and New York, NY, USA: Cambridge University Press; 2013.

[9] IPCC. Climate Change 2014: Impacts, Adaptation and Vulnerability. Field CB, Barros VR, Mastrandrea M, Mach KJ, Abdrabo M, Adger W, et al, editors. Cambridge University Press; 2014.

[10] McCain CM, Colwell RK. Assessing montane biodiversity from discordant shifts in temperature and precipitation in a changing climate. Ecology Letters. 2007;14:1236–1245. 10.1111/j.1461-0248.2011.01695.x 21981631

[11] Chen IC, Hill JK, Ohlemüller R, Roy DB, Thomas CD. Rapid range shifts of species associated with high levels of climate warming. Science. 2011;20(333):1024–1026. 10.1126/science.1206432 21852500

[12] Lenoir J, Gégout JC, Guisan A, Vittoz P, Wohlgemuth T, Zimmermann NE, et al Going against the flow: Potential mechanisms for unexpected downslope range shifts in a warming climate. Ecography. 2010;33(2):295–303.

[13] Tingley MW, Koo MS, Moritz C, Rush AC, Beissinger SR. The push and pull of climate change causes heterogeneous shifts in avian elevational ranges. Global Change Biology. 2012;18:3279–3290. 10.1111/j.1365-2486.2012.02784.x

[14] Rybicki J, Hanski I. Species-area relationships and extinctions caused by habitat loss and fragmentation. Ecology Letters. 2013;16:27–38. 10.1111/ele.12065 23452159

[15] Theurillat JP, Guisan A. Potential impact of climate change on vegetation in the European Alps: a review. Climatic Change. 2001;50:77–109. 10.1023/A:1010632015572

[16] Guisan A, Theurillat JP. Assessing alpine plant vulnerability to climate change: a modeling perspective. Integrated Assessment. 2001;1(1):307–320.

[17] Dullinger S, Gattringer A, Thuiller W, Moser D, Zimmermann N, Guisan A, et al Extinction debt of high-mountain plants under twenty-first-century climate change. Nature Climate Change. 2012;2(8):619–622. 10.1038/nclimate1514

[18] Tilman D, May RM, Lehman CL, Nowak MA. Habitat destruction and the extinction debt. Nature. 1994;371(6492):65–66. 10.1038/371065a0

[19] Steinbauer M, Grytnes JA, Jurasinski G, Kulonen A, Lenoir J, Pauli H, et al Accelerated increase in plant species richness on mountain summits is linked to warming. Nature. 2018;556(7700):231 10.1038/s41586-018-0005-6 29618821

[20] Seebens H, Blackburn TM, Dyer EE, Genovesi P, Hulme PE, Jeschke JM, et al Global rise in emerging alien species results from increased accessibility of new source pools. Proceedings of the National Academy of Sciences of the United States of America. 2018;115(10):E2264–E2273. 10.1073/pnas.1719429115 29432147 PMC5877962

[21] Vetaas OR, Grytnes JA. Distribution of vascular plant species richness and endemic richness along the Himalayan elevation gradient in Nepal. Global Ecology and Biogeography. 2002;11(4):291–301. 10.1046/j.1466-822X.2002.00297.x

[22] MacArthur RH. Geographical Ecology. New York: Harper and Rowe Publishers; 1972.

[23] Brown JH. Macroecology. The University of Chicago Press; 1995.

[24] Körner C. The use of ‘altitude’ in ecological research. Trends in Ecology & Evolution. 2007;22(11):569–574. 10.1016/j.tree.2007.09.006 17988759

[25] Nogues-Bravo D, Araujo MB, Romdal T, Rahbek C. Scale effects and human impact on the elevational species richness gradients. Nature. 2008;453:216–219. 10.1038/nature06812 18464741

[26] McCain CM, Grytnes JA. Elevational Gradients in Species Richness. Encylcopedia of Life Sciences. 2010;15:1–10.

[27] Lenoir J, Gegout JC, Marquet PA, de Ruffray P, Brisse H. A significant upward shift in plant species optimum elevation during the 20th century. Science. 2008;320(5884):1768–1771. 10.1126/science.1156831 18583610

[28] Marquet PA, Allen AP, Brown JH, Dunne JA, Enquist BJ, Gillooly JF, et al On theory in ecology. Bioscience. 2014;64(8):701–710. 10.1093/biosci/biu098

[29] Wiens JA. Metapopulation Dynamics and Landscape Ecology In: Hanski I, Gilpin ME, editors. Metapopulation Biology. San Diego: Academic Press; 1997 p. 43–62.

[30] Economo EP, Keitt TH. Species diversity in neutral metacommunities: a network approach. Ecology Letters. 2008;11(1):52–62. 10.1111/j.1461-0248.2007.01126.x 18021244

[31] Muneepeerakul R, Bertuzzo E, Lynch HJ, Fagan WF, Rinaldo A, Rodriguez-Iturbe I. Neutral metacommunity models predict fish diversity patterns in Mississippi Missouri basin. Nature. 2008;453:220–223. 10.1038/nature06813 18464742

[32] Economo EP, Keitt TH. Network isolation and local diversity in neutral metacommunities. Oikos. 2010;119(8):1355–1363. 10.1111/j.1600-0706.2010.18272.x

[33] Bertuzzo E, Suweis S, Mari L, Rodriguez-Iturbe I, Rinaldo A. Spatial effects on species persistence and implications for biodiversity. Proceedings of the National Academy of Sciences of the United States of America. 2011;108:4346–4351. 10.1073/pnas.1017274108 21368181 PMC3060266

[34] Carrara F, Altermatt F, Rodriguez-Iturbe I, Rinaldo A. Dendritic connectivity controls biodiversity patterns in experimental metacommunities. Proceedings of the National Academy of Sciences of the United States of America. 2012;109:5761–5766. 10.1073/pnas.1119651109 22460788 PMC3326484

[35] Rodríguez-Iturbe I, Rinaldo A. Fractal river basins: chance and self-organization. Cambridge University Press; 2001.

[36] Colwell RK, Lees DC. The mid-domain effect: Geometric constraints on the geography of species richness. Trends in Ecology & Evolution. 2000;15(2):70–76. 10.1016/S0169-5347(99)01767-X 10652559

[37] Jacob S, Laurent E, Haegeman B, Bertrand R, Prunier JG, Legrand D, et al Habitat choice meets thermal specialization: competition with specialists may drive suboptimal habitat preferences in generalists. Proceedings of the National Academy of Sciences of the United States of America. 2018;115(47):1–33. 10.1073/pnas.1805574115 PMC6255147 30397109

[38] Harrison S, Taylor AD. Empirical Evidence for Metapopulation Dynamics In: Hanski I, Gilpin ME, editors. Metapopulation Biology. San Diego: Academic Press; 1997 p. 27–42.

[39] Hanski I. Metapopulation dynamics. Nature. 1998;396(6706):41–49. 10.1038/23876

[40] Thibaud E, Petitpierre B, Broennimann O, Davison AC, Guisan A. Measuring the relative effect of factors affecting species distribution model predictions. Methods in Ecology and Evolution. 2014;5(9):947–955. 10.1111/2041-210X.12203

[41] Fernandes RF, Scherrer D, Guisan A. How much should one sample to accurately predict the distribution of species assemblages? A virtual community approach. Ecological Informatics. 2018;48:125–134. 10.1016/j.ecoinf.2018.09.002

[42] Fernandes RF, Scherrer D, Guisan A. Effects of simulated observation errors on the performance of species distribution models. Diversity and Distributions. 2018; p. 1–14.

[43] Guisan A, Thuiller W, Zimmermann NE. Habitat Suitability and Distribution Models With Applications in R. Cambridge University Press; 2017.

[44] Pasetto D, Arenas-Castro S, Bustamante J, Casagrandi R, Chrysoulakis N, Cord AF, et al Integration of satellite remote sensing data in ecosystem modelling at local scales: Practices and trends. Methods in Ecology and Evolution. 2018;9(8):1810–1821. 10.1111/2041-210X.13018

[45] Hanski I, Ovaskainen O. The metapopulation capacity of a fragmented landscape. Nature. 2000;404:755–758. 10.1038/35008063 10783887

[46] Moilanen A. SPOMSIM: Software for stochastic patch occupancy models of metapopulation dynamics. Ecological Modelling. 2004;179(4):533–550. 10.1016/j.ecolmodel.2004.04.019

[47] Hanski I. Metapopulation ecology. Oxford University Press; 1999.

[48] Ovaskainen O, Hanski I. Spatially structured metapopulation models: Global and local assessment of metapopulation capacity. Theoretical Population Biology. 2001;60:281–302. 10.1006/tpbi.2001.1548 11878830

[49] Gu W, Heikkilä R, Hanski I. Estimating the consequences of habitat fragmentation on extinction risk in dynamic landscapes. Landscape Ecology. 2002;17:699–710. 10.1023/A:1022993317717

[50] Papaïx J, David O, Lannou C, Monod H. Dynamics of Adaptation in Spatially Heterogeneous Metapopulations. PLoS One. 2013;8(2). 10.1371/journal.pone.0054697 23424618 PMC3570538

[51] Bertuzzo E, Rodriguez-Iturbe I, Rinaldo A. Metapopulation capacity of evolving fluvial landscapes. Water Resources Research. 2015;51(4):2696–2706. 10.1002/2015WR016946

[52] Barry RG, Chorley RJ. Atmosphere, weather and climate. London: Routledge; 2009.

[53] Bertrand R, Lenoir J, Piedallu C, Dillon GR, De Ruffray P, Vidal C, et al Changes in plant community composition lag behind climate warming in lowland forests. Nature. 2011;479(7374):517–520. 10.1038/nature10548 22012261

[54] Devictor V, Van Swaay C, Brereton T, Brotons L, Chamberlain D, Heliölö J, et al Differences in the climatic debts of birds and butterflies at a continental scale. Nature Climate Change. 2012;2(2):121–124. 10.1038/nclimate1347

[55] Alexander JM, Diez JM, Hart SP, Levine JM. When climate reshuffles competitors: a call for experimental macroecology. Trends in Ecology & Evolution. 2016;31(11):831–841. 10.1016/j.tree.2016.08.003 27640784 PMC5159619

[56] Guisan A, Thuiller W. Predicting species distribution: Offering more than simple habitat models. Ecology Letters. 2005;8(9):993–1009. 10.1111/j.1461-0248.2005.00792.x 34517687

[57] Kéry M, Guillera-Arroita G, Lahoz-Monfort JJ. Analysing and mapping species range dynamics using occupancy models. Journal of Biogeography. 2013;40(8):1463–1474. 10.1111/jbi.12087

[58] Carraro L, Mari L, Gatto M, Rinaldo A, Bertuzzo E. Spread of proliferative kidney disease in fish along stream networks: A spatial metacommunity framework. Freshwater Biology. 2018;63:114–127. 10.1111/fwb.12939

[59] Banavar JR, Colaiori F, Flammini A, Maritan A, Rinaldo A. Scaling, optimality and landscape evolution. Journal of Statistical Physics. 2001;104:1–33. 10.1023/A:1010397325029

[60] Rinaldo A, Rigon R, Banavar JR, Maritan A, Rodriguez-Iturbe I. Evolution and selection of river networks: Statics, dynamics, and complexity. Proceedings of the National Academy of Sciences of the United States of America. 2014;111(7):2417–2424. 10.1073/pnas.1322700111 24550264 PMC3932906

[61] Balister P, Balogh J, Bertuzzo E, Bollobás B, Caldarelli G, Maritan A, et al River landscapes and optimal channel networks. Proceedings of the National Academy of Sciences of the United States of America. 2018;115(26):6548–6553. 10.1073/pnas.1804484115 29891709 PMC6042144

[62] Engler R, Randin CF, Vittoz P, Czaka T, Beniston M, Zimmermann NE, et al Predicting future distributions of mountain plants under climate change: does dispersal capacity matter? Ecography. 2009;32:34–45. 10.1111/j.1600-0587.2009.05789.x

