# Supplementary material for: A minimalist model of extinction and range dynamics of virtual mountain species driven by warming temperatures
Source: PLoS One. 2019 Mar 18;14(3):e0213775. doi: 10.1371/journal.pone.0213775 (PMC6422262; doi:10.1371/journal.pone.0213775)
Supplement: S1 File — (PDF) [file pone.0213775.s001.pdf]

Appendix S 1

A minimalist model of extinction and range dynamics of mountain species driven by warming temperatures

Jonathan Giezendanner[1*], Enrico Bertuzzo[1,2], Damiano Pasetto[1], Antoine Guisan[3], Andrea Rinaldo[1,4]

1 Laboratory of Ecohydrology, École Polytechnique Fédérale de Lausanne, 1015 Lausanne CH
2 Department of Environmental Sciences, Informatics and Statistics, University Cà Foscari Venice, 30123 Venezia Mestre IT
3 Department of Ecology and Evolution, University of Lausanne, 1015 Lausanne CH
4 Dipartimento ICEA, Università di Padova, 35131 Padova IT

* jonathan.giezendanner@epfl.ch

S 1 Species parameters trade-off

S 1.1 Comparable species viability

This section, meant as an addendum of section 'Comparable species viability' in the methods, provides a numerical counterpart to the theoretical justifications made in the main text (figure S1.1), and an example of results when allowing super species in the simulation (figure S1.2).

S 1.1.1 Numerical Proof

As the demonstration ensures consistency among the upper bound of the metapopulation capacity of different species, it is interesting to numerically verify the actual metapopulation capacity values in a similar conceptual landscape. Figure S1.1 shows the numerical results obtained computing the metapopulation capacity and its upper bound for different species (σ = 16, 32, 64, 128, 256, and 512) in a domain of size $L = 1500$ and different discretization refinements, Δx=0.1, 0.5, 1.0. Numerically, at decreasing values of Δx the metapopulation capacity λ_M values associated with different species tend to the same constant (see Appendix 1 for numerical examples), while the numerical upper bound proves a good estimate of the theoretical value $\sqrt{2\pi}$. With decreasing distance between the elements, the metapopulation capacity $\lambda_{\max}$ values associated to different species tend to the same constant, while the numerical upper bound is a good estimation of the theoretical value $\sqrt{2\pi}$.

Figure S1.2 shows how the parameter space gets clouded by super species able to survive everywhere when not considering comparable species viability, rendering any effort to display geomorphic effects meaningless.

S 1.2 Dispersal tradeoff

As an additional normalization step to ensure species inter-comparability, the exponential dispersal function $\mathcal{D}$ between two patches with distance d is designed such

that the kernel respects a unitary volume for all species with different dispersal parameters D [1]:

$$\mathcal{D} = \exp\left(-d/D\right)/\left(2\pi D^2\right) \tag{1}$$

Proof:

$$\int\int_{\mathrm{I\!R}^2} \frac{\exp\left(-\sqrt{x^2+y^2}/D\right)}{2\pi D^2} \mathrm{d}x\mathrm{d}y \tag{2a}$$

$$\overset{\text{polar}}{\Rightarrow} \int_0^{2\pi}\int_0^{\infty} d\mathcal{D}\ \mathrm{d}d\mathrm{d}\theta = 1\ \forall D \tag{2b}$$

This ensures that two species with different dispersal coefficients have the same dispersal volume [1].

References

1. Rybicki J, Hanski I. Species-area relationships and extinctions caused by habitat loss and fragmentation. Ecology Letters. 2013;16:27–38.

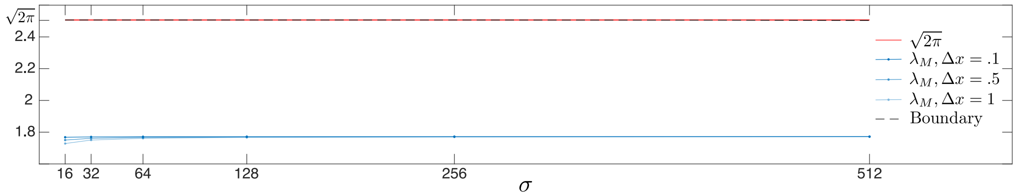

Fig S1.1. Comparison between the upper bound computed using the Perron-Frobenius theorem (black line), the theoretical value (red line), and the largest eigenvalue with different values of Δx

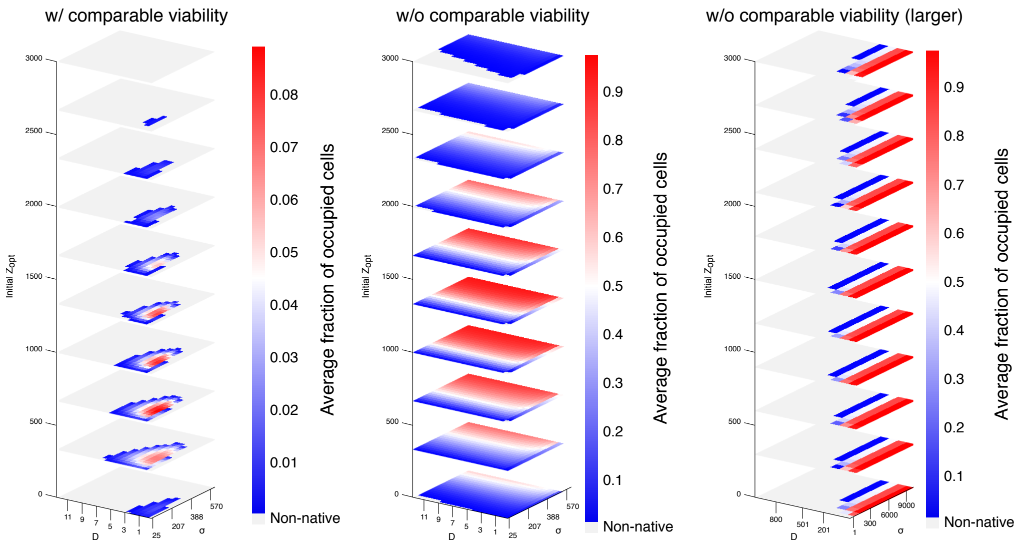

Fig S1.2. Comparison between the fraction of occupied cells for different species in the OCN landscape with comparable species viability (left panel) or without (central and right panels). The central and right panels show that w/o comparable viability, with increasing values of niche width, the fraction of occupied space tends towards one, as all space is suited for these species when the niche becomes large enough.
