# Supplementary material for: A minimalist model of extinction and range dynamics of virtual mountain species driven by warming temperatures
Source: PLoS One. 2019 Mar 18;14(3):e0213775. doi: 10.1371/journal.pone.0213775 (PMC6422262; doi:10.1371/journal.pone.0213775)
Supplement: S2 File — (PDF) [file pone.0213775.s002.pdf]

Appendix S 2
A minimalist model of extinction and range dynamics of mountain species driven by warming temperatures

Jonathan Giezendanner[1*], Enrico Bertuzzo[1,2], Damiano Pasetto[1], Antoine Guisan[3], Andrea Rinaldo[1,4]

1 Laboratory of Ecohydrology, École Polytechnique Fédérale de Lausanne, 1015 Lausanne CH
2 Department of Environmental Sciences, Informatics and Statistics, University Cà Foscari Venice, 30123 Venezia Mestre IT
3 Department of Ecology and Evolution, University of Lausanne, 1015 Lausanne CH
4 Dipartimento ICEA, Università di Padova, 35131 Padova IT

* jonathan.giezendanner@epfl.ch

S 2 Building statistically identical replicas of mountain topographies

S 2.1 OCNs

We consider, without loss of generality [1, 2], landscapes formed by square lattices with side L with $N = L \times L$ pixels (nodes). Each node has a link to one of its eight nearest neighbours forming a spanning tree with a single root – the outlet. To each pixel i of the tree, total contributing area A_i (the number of upstream pixels connected to i through flow directions) is expressed in pixel units as

$$A_i = \sum_j W_{ji} A_j + 1, \quad (1)$$

where W_{ji} is the arbitrary element of the connectivity matrix $\mathbf{W}$ (i.e. $W_{ji} = 1$ if $j \to i$ and 0 otherwise), and 1 represents the unit area of the pixel that discretizes the surface. A_i provides a proxy of the flow at point i, i.e. accumulated flow Q_i, as the sum of the injections over all connected sites upstream of site i (included) $Q_i = \sum_j W_{ji} Q_j + r_i$ (where r_i is the distributed injection). In the case of uniform injection r_i for landscape-forming events, one has $Q_i \propto A_i$, a commonly accepted hydrologic assumption. The tree configuration is uniquely determined by the set of total contributing areas $s = (A_1, A_2, \cdots, A_N)^T$ at any of the N sites making up the landscape. From equation 1 one has $s = \left(\mathbf{I} - \mathbf{W^T}\right)^{-1}$ ($\mathbf{I}$ being the identity matrix). A necessary condition for the existence of s is that the matrix at right-hand side can be inverted. In turn, this corresponds to specific spectral properties of the connectivity matrix $\mathbf{W}$ implying uniqueness of the paths from any site to the outlet i.e. s must be a tree [3].

Optimal Channel Networks (OCNs) are spanning trees minimizing a functional describing total energy dissipation $H(s)$ of the aggregate's configuration s. At a local level, say, along the i-th link of the network, energy dissipation is $H_i \propto Q_i \Delta z_i$, which makes use of suitable landscape-forming discharges ($Q_i \sim A_i$) and of the drop in

elevation along a drainage direction. Theory and field evidence indicate $\Delta z_i \sim A_i^{\gamma-1}$ with $\gamma = 0.5$ [1]. Spanning, loopless network configurations characterized by minimum energy dissipation are thus obtained by selecting the configuration s that minimizes the functional [3–5]:

$$H_\gamma(s) \ = \ \sum_{i=1}^{N} H_i \ \propto \ \sum_{i=1}^{N} A_i^\gamma. \tag{2}$$

In the selection process one needs to choose consistently tree-like configurations. This, in turn, is exactly admissible because every tree is a local minimum of total energy expenditure [6] whenever one has $\gamma < 1$ directly from the physics of the problem recapitulated by a deterministic slope-area relation [2, 7].

We adopt a simulated annealing strategy to find OCNs [3]. The algorithm starts from an initial network configuration chosen to span the whole $L \times L$ landscape towards an imposed outlet, chosen at a single or multiple sites [1]. Every network configuration is completely described by the connectivity matrix $\mathbf{W}$. At every iteration of the optimization process, one pixel is drawn at random and its link is rewired to a different nearest neighbour, provided that no loops are formed. This change affects the matrix' elements W_{ij} and, in turn, the values of the aggregated areas A_i. The change in configuration $s \to s'$ is always accepted if total energy dissipation is decreased (i.e. $H_\gamma(s') < H_\gamma(s)$). Perturbations to higher values may be accepted, however, with a probability that depends on a parameter T, termed 'temperature' owing to the thermodynamic annealing analog. On following a schedule of decreasing values of T, the network resulting from a perturbation is accepted according to the Metropolis rule, i.e., if

$$\exp\left(-\frac{H_\gamma(s') - H_\gamma(s)}{T}\right) \ < \ R \tag{3}$$

where R is a random number drawn from a uniform distribution in the interval $(0, 1)$. In this manner, when the temperature is initially high, unfavorable changes are likely to be accepted thus making the algorithm capable of jumping out of structures conditioned by the initial condition. As the temperature is reduced, changes are only accepted if they lower total energy dissipation and thus the optimum configuration gets refined and 'frozen' around a dynamically accessible minimum.

The exact linkage of OCNs with the stationary solutions of the general landscape evolution equation [7] allows us to treat OCNs as replicable planar constructs that reproduce statistically identical sets of drainage directions corresponding to stationary states of a landscape evolution equation where fluvia erosion and geologic uplift are balanced.

S 2.2 From OCNs to mountain topographies

A 3D landscape topography is associated to a planar OCN via the following procedure. Recall that a slope-area law is implied in the value of γ i.e. at site i, the upstream drop in elevation at the i-th site [1] is $\Delta z_i = \mathcal{C} A_i^{-1/2}$ (where $\mathcal{C}$ is a constant determining ultimately the overall relief of the synthetic topography, and A_i is the total contributing area at i). Moving from an outlet, whose elevation is set to 0, one follows the connectivity to attribute the elevation of the j nodes connected to it. Owing to the exact tree-like features (there exist a unique path from any site j to the outlet because loops are excluded [7] if $\gamma < 1$. Field observations and theoretical results suggest values $\gamma \approx 1/2$) such that a topography is uniquely associated with any OCN. It should be noted that the validity of the slope-area law is restricted to the channeled portion of the landscape where fluvial erosion and uplift are the dominating, balanced landscape-forming processes [1]. Suffice here to say that this is tantamount to assume

that the scale of the landscape must then be much larger than the (inverse of) the drainage density of the river basin [1]. Should one be interested in reproducing a proper mountain range, one could selectively choose the value of C. For the scopes of the present work, it is deemed that a constant value of $\mathcal{C}$ is an appropriate choice together with the assumption that the drainage density is contained witin the pixel size. In the present theoretical experiments the constant is regulated such that the relief is confined to a standard relief of 3000 m, i.e. elevation ranges from 0 to 3000 m to produce a physically meaningful context.

When standard DEMs are employed (cfr Figs. 1 and 4), local fitness at i, f_i, is directly obtained from the given elevation field z_i or from other landscape attributes (Section S3). The DEMs were retrieved from the online earth explorer tool, courtesy of the NASA EOSDIS Land Processes Distributed Active Archive Center (LP DAAC), USGS/Earth Resources Observation and Science (EROS) Center, Sioux Falls, South Dakota, `https://earthexplorer.usgs.gov/`.

References

1. Rodríguez-Iturbe I, Rinaldo A. Fractal river basins: chance and self-organization. Cambridge University Press; 2001.

2. Rinaldo A, Rigon R, Banavar JR, Maritan A, Rodriguez-Iturbe I. Evolution and selection of river networks: Statics, dynamics, and complexity. Proceedings of the National Academy of Sciences of the United States of America. 2014;111(7):2417–2424.

3. Rinaldo A, Rodriguez-Iturbe I, Rigon R, Bras RL, Ijjasz-Vasquez E, Marani A. Minimum energy and fractal structures of drainage networks. Water Resources Research. 1992;28:2183–2195.

4. Rodriguez-Iturbe I, Rinaldo A, Rigon R, Bras RL, Ijjasz-Vasquez E. Energy dissipation, runoff production and the three dimensional structure of channel networks. Water Resources Research. 1992;28(4):1095–1103.

5. Rodriguez-Iturbe I, Rinaldo A, Rigon R, Bras RL, Ijjasz-Vasquez E. Fractal structures as least energy patterns: the case of river networks. Geophysical Research Letters. 1992;19(9):889–892.

6. Banavar JR, Maritan A, Rinaldo A. Topology of the fittest network. Phys Rev Lett. 2000;84:4745–4748.

7. Banavar JR, Colaiori F, Flammini A, Maritan A, Rinaldo A. Scaling, optimality and landscape evolution. Journal of Statistical Physics. 2001;104:1–33.

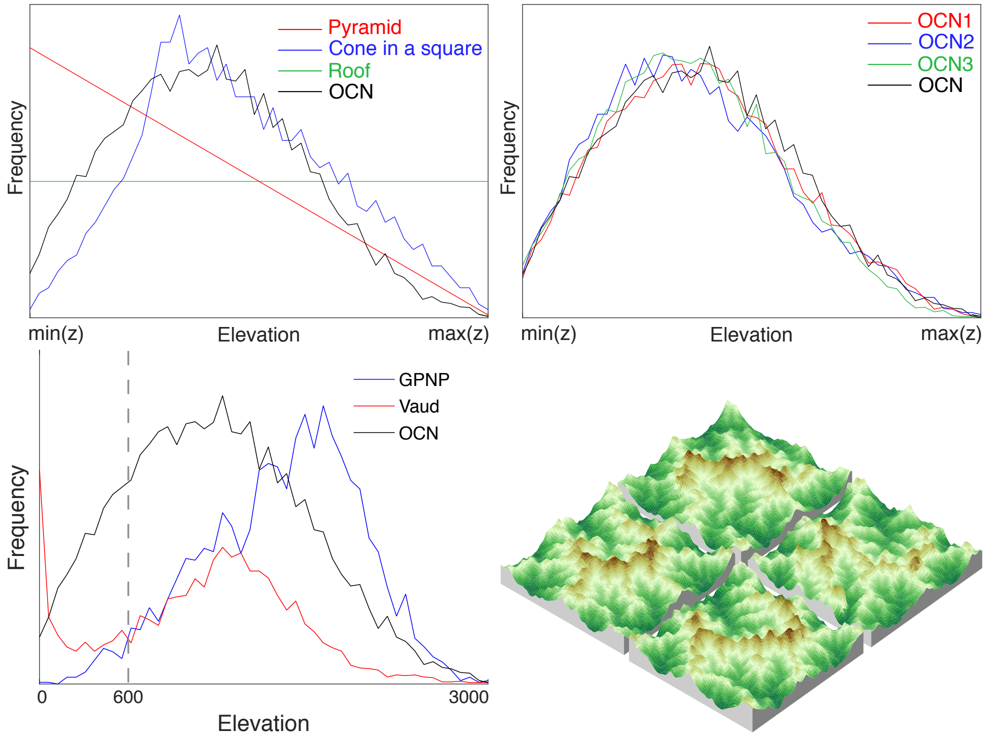

Fig S2.1. Hypsographic curves of the 8 landscapes of interest, grouped by type of landscape, with the OCN as reference in each of them. The dashed line in the right panel shows the elevation-limit creating a species-void by climate change. Note that the elevation relief has been re-scaled from 0 to 3000 to match the other landscapes' relief. The elevation fields show the four OCNs employed in the experiment, the one on the left being refereed to in the main text.
