# Supplementary material for: A minimalist model of extinction and range dynamics of virtual mountain species driven by warming temperatures
Source: PLoS One. 2019 Mar 18;14(3):e0213775. doi: 10.1371/journal.pone.0213775 (PMC6422262; doi:10.1371/journal.pone.0213775)
Supplement: S3 File — (PDF) [file pone.0213775.s003.pdf]

Appendix S 3
A minimalist model of extinction and range dynamics of mountain species driven by warming temperatures

Jonathan Giezendanner[1*], Enrico Bertuzzo[1,2], Damiano Pasetto[1], Antoine Guisan[3], Andrea Rinaldo[1,4]

1 Laboratory of Ecohydrology, École Polytechnique Fédérale de Lausanne, 1015 Lausanne CH
2 Department of Environmental Sciences, Informatics and Statistics, University Cà Foscari Venice, 30123 Venezia Mestre IT
3 Department of Ecology and Evolution, University of Lausanne, 1015 Lausanne CH
4 Dipartimento ICEA, Università di Padova, 35131 Padova IT

* jonathan.giezendanner@epfl.ch

S 3 Computational experiment

S 3.1 Landscapes hypsographic curves and landscapes choices

Eight different landscapes have been used to understand the geomorphic effects on metapopulation dynamics: three simple geometric landscapes (roof, cone-in-a-square and pyramid), four different realizations of realistic synthetic landscapes (OCNs) with similar statistical properties, and two real landscapes (*Gran Paradiso National Park* in Italy and *Vaud Alpes* in Switzerland).

The roof allows to understand how, given the same available area at each altitude, the increased connectivity towards the outer elevations of the distribution influences the distribution. The pyramid allows to comprehend how species handle decreasing available area, and the cone in a square, with its very similar hypsographic curve to the OCN (see fig. S 2.1), permits to comprehend the strong influence of complex connectivity. The elevational border of the various domain (mountain tops and valleys) are expected to influence the presence of a given species by blocking the realized niche from developing into the theoretical niche, decreasing the number of favorable cells.

The real landscapes permit to further investigate the metapopulation dynamics when connectivity is not only influenced by fluvial erosion, like this is assumed for the OCNs.

S 3.2 Simulation and additional results

As described in Figs. 1 and 5 of the main text, the experiment is done in several steps, the details of these steps are presented here. The initial stage is designed to identify the species supposed to exist in the landscape, the 'native' species. To this intend the experiment is run from a fully occupied landscape until reaching a steady state (step 0). For each species, this first step is repeated a 100 times, and species with at least 50% of the runs leading to at least one cell in the landscape being occupied are considered to be native species. The results of this step are shown in Figs. S3.1, S3.5, S3.9, S3.13, S3.17, S3.21, S3.25, S3.29 and S3.33, left the count of the species with at least one occupied cell at steady state, and center the initial average fraction of occupied cells.

The spatial occupancy generated after the initial step (state 1) is then used for step 2. For species with less than 100 runs leading to occupied landscapes, the remaining initial configurations are generated by bootstrapping from the other occupied configurations. Climate change is then applied, following the reasoning described in the methods (step 2). The fraction of occupied cells after this step (state 2) is depicted in Figs. S3.1, S3.5, S3.9, S3.13, S3.17, S3.21, S3.25, S3.29 and S3.33 (right). Here, a first classification is done: the species being able to track climate change, vs the species unable to track climate change (going extinct). For the species going extinct, an additional simulation is done (step 4 and 5), the landscape is again fully occupied and run to steady state, but with the species' optimal elevation after climate change. This allows to differentiate between species going extinct because they are unsuited, and species going extinct because they are unable to track the change but would have been able to survive in the new conditions. The number of runs leading to these outcomes are displayed in figure S3.3, S3.7, S3.11, S3.15, S3.19, S3.23, S3.27, S3.31 and S3.35 (left and center).

The last step (step 3) is run to see which species were able to follow climate change, but are actually not suited to the new conditions, the extinction debt, and, once at steady state, to see which species increase or decrease their presence.

Finally, taking into account the fates towards which the different steps of the simulation lead, a classification can be made for each species, which can be seen in Figs. S3.4, S3.8, S3.12, S3.16, S3.20, S3.24, S3.28, S3.32 and S3.36 (center).

Figure S3.1 to S3.36 present the results and the intermediate steps of the simulation of the experiment for the different landscapes of interest: Roof, Pyramid, Cone in a square, OCN (0,1,2,3), Gran Paradiso National Park (IT) and Alpes Vaudoises (CH).

S 3.3 Effect of hypsographic curve and connectivity

An interesting effect can be observed in the roof, where all elevations show the same available area (flat hypsographic curve), and the upper and lower elevations, i.e. the mountain-top and valley, affect the species by cutting the niche. This effect can also be observed in the bottom part of the pyramid where elevations close to the mid-bottom display a larger presence than the elevation band at the bottom, suggesting a strong border effect. Another effect can be observed in the roof-shape: areal presence is maximal towards the outer elevation bands, suggesting that the closer presence of similar areas towards the outer bands, i.e. increased connectivity, allows certain species to more efficiently maintain the equilibrium between extinction and colonization, as suggested by the landscape capacity plots. Interestingly, the cone and the OCN do not display the same presence patterns although endowed with similar elevation distributions. The OCN shows a more concentrated region of suitable parameter values, suggesting an important effect of the spatial aggregation of suitable area and a strong effect of landscape fragmentation, as confirmed by the landscape capacity plots.

Under climate change, increased connectivity and reduced border effect seem to positively affect species presence when moving toward mid-domain, even in landscapes displaying monotonically decreasing hypsographic curves, as for instance the case in the pyramid.

S 3.4 Connectivity and proximity to similar elevation

Figs. S3.40,S3.41,S3.39 show the average proximity to similar area of each pixel for the different landscapes at different radiuses around the pixel, using the formula $\overline{z_i} = -\log(\sqrt{\frac{\sum_j^N (z_j - z_i)^2}{N}})$, where N represents the number of pixels within a radius r around pixel i. For the OCN, one can see that close-by area is very similar in the valleys, but start to move to the mid-domain if considering a larger radius. The geometric

landscapes behave in a similar fashion, where regions at the border of the domain are more sensitive to areas in a small radius (probably because of the periodicity of the landscape), and when larger radii are used, the mid-domain becomes more relevant.

S 3.5 Effect on species diversity

Figure S3.44 shows the α-diversity (i.e. the number of species, among the selected pool of native species, that simultaneously occupy a patch) in the OCN for the different phases of the experiment, i.e. initially, after climate change and at steady state after climate change, computed from a discrete choice of values of σ, D and z_{opt}. Therein, an initially high α-diversity is shown towards the lowest regions of the OCNs where the proximity (shown in Figs. S3.40, S3.41, S3.39) of suitable area is higher, but there is lower available area and realized niche than at the mid-elevations, thus creating a left-skewed hump-shaped distribution. Under climate change, the peak of α-diversity is naturally shifted towards the mid-domain as species start moving upwards, and no other possibly invading species are considered (the species pool is not changing). The scatter plot in Fig. S3.44 shows how the average α-diversity at mid-elevation tends to increase compared to the initial situation, only to later stabilize and end up at a lower level than the initial value. Species lagging in adapting their distribution to the actual optimal range imposed by climate change overlap with species catching up from below, creating an artificial temporal elevated value of α-diversity, which is then lowered by the species moving towards their respective ranges at steady state, and other species going extinct in the process.

Different currencies exist (correlative, mechanistic, and trait-based) to assess species' climate change vulnerability [1]. For example, α- and β-diversities are often viewed as suitable measures to assess the change occurring at a certain location [2, 3]. In this context, Fig. S3.44 suggests that such indicators have to be handled with caution. In fact, in high elevations, unaffected by the species pool, climate change seems to show an increase in α-diversity compared to the initial value. This temporary increase is generated by a lag of species in the process of adapting to their new habitat, which might be interpreted as an early warning.

References

1. Pacifici M, Foden W, Visconti P, Watson J, Butchart HMS, Kovacs K, et al. Assessing species vulnerability to climate change. Nature Climate Change. 2015;5:215–224.

2. Whittaker RH. Evolution and Measurement of Species Diversity. Taxon. 1972;21(2):213–251.

3. Anderson MJ, Crist TO, Chase JM, Vellend M, Inouye BD, Freestone AL, et al. Navigating the multiple meanings of beta diversity: A roadmap for the practicing ecologist. Ecology Letters. 2011;14(1):19–28.

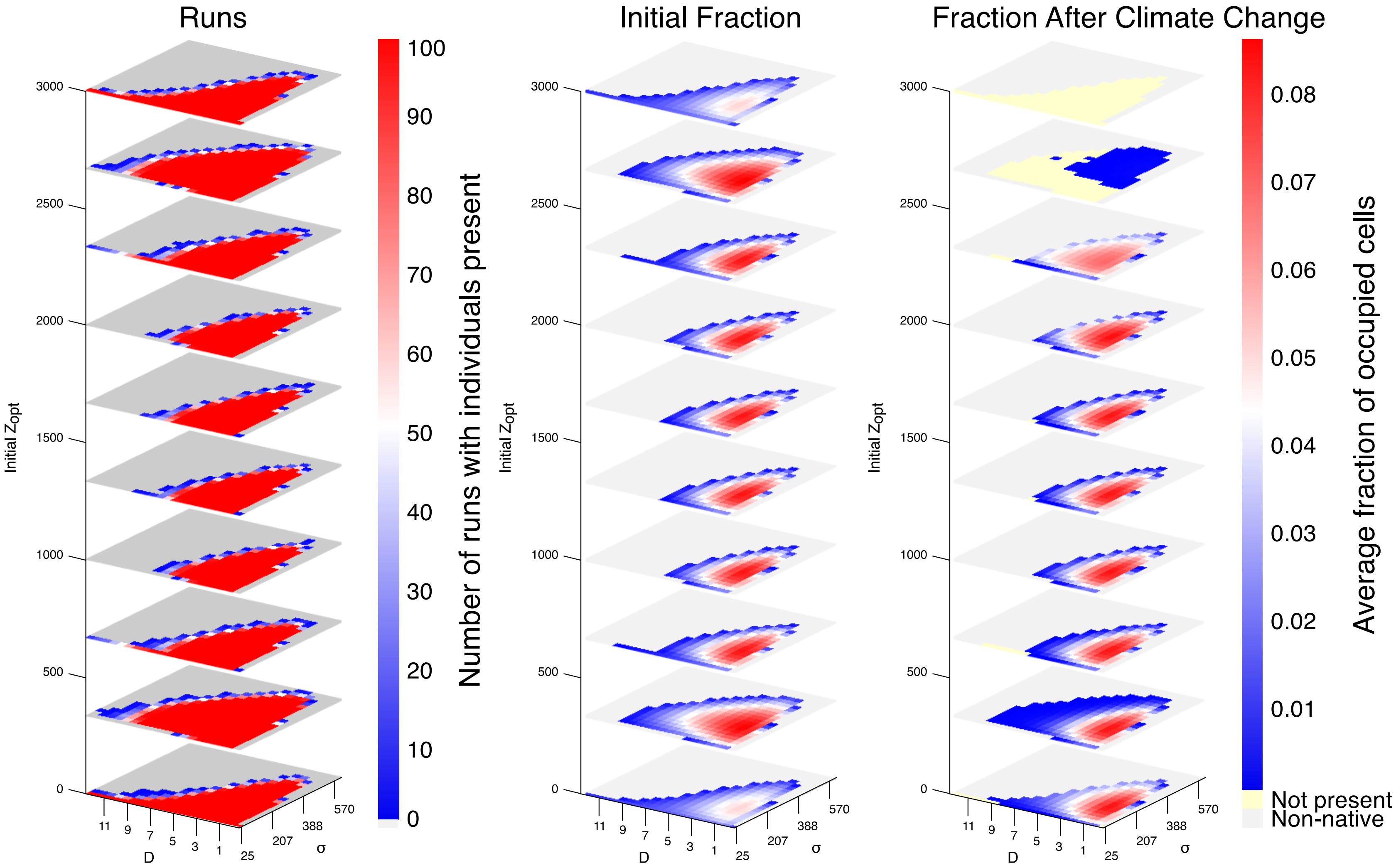

Fig S3.1. Intermediate results for the 'Roof' landscape. Number of runs leading to at least one occupied cell after the initial phase (left). Average fraction of occupied cell in the 100x100 grid-system over 100 runs, after the initial phase (center), and after climate change (right).

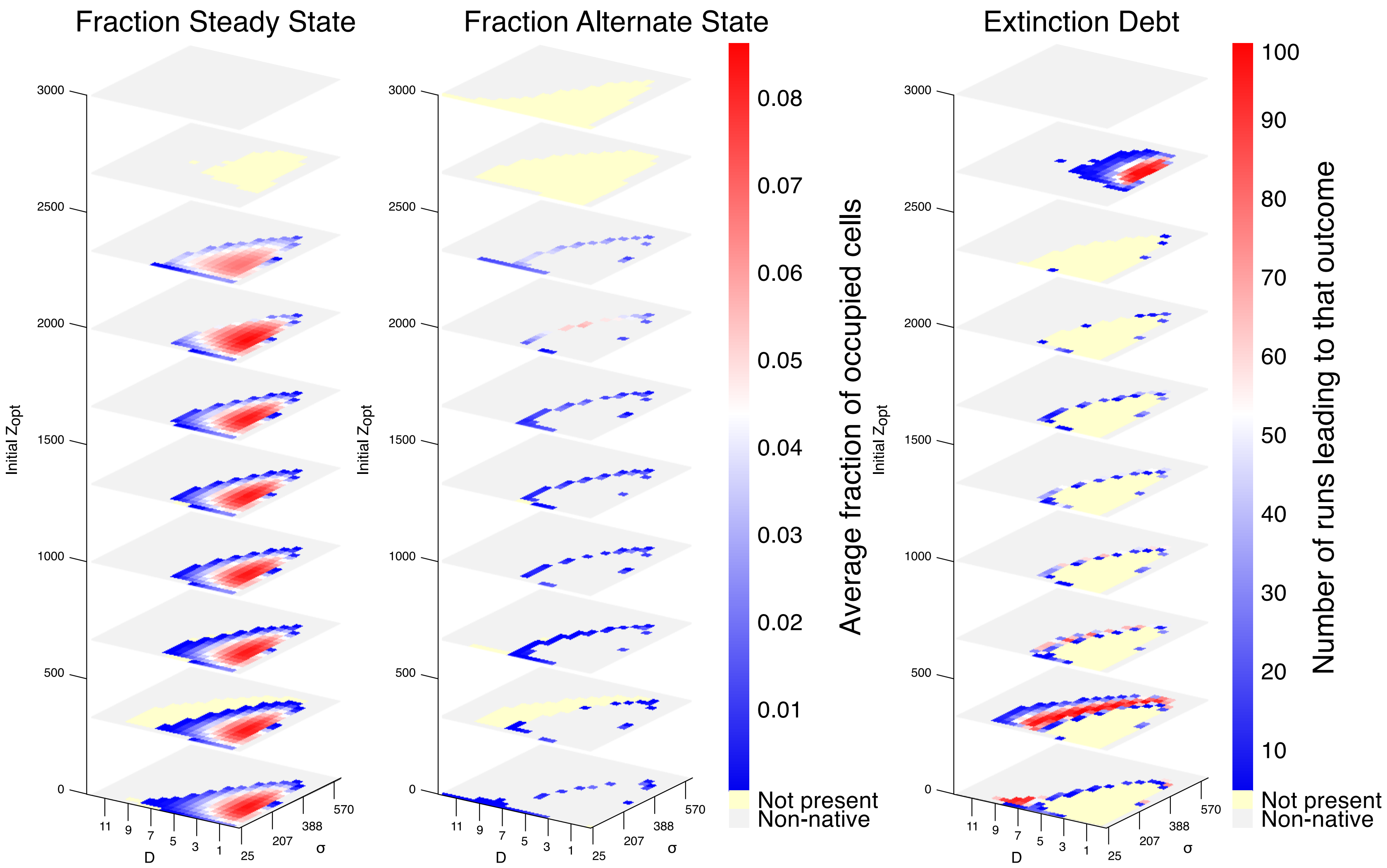

Fig S3.2. Intermediate results for the 'Roof' landscape. Average fraction of occupied cell in the 100x100 grid-system over 100 runs, at steady state after climate change (left), and at the alternate state for the species going extinct during the climate change phase (see methods) (center). Number of runs leading to the fate 'Extinction debt' for each species (right).

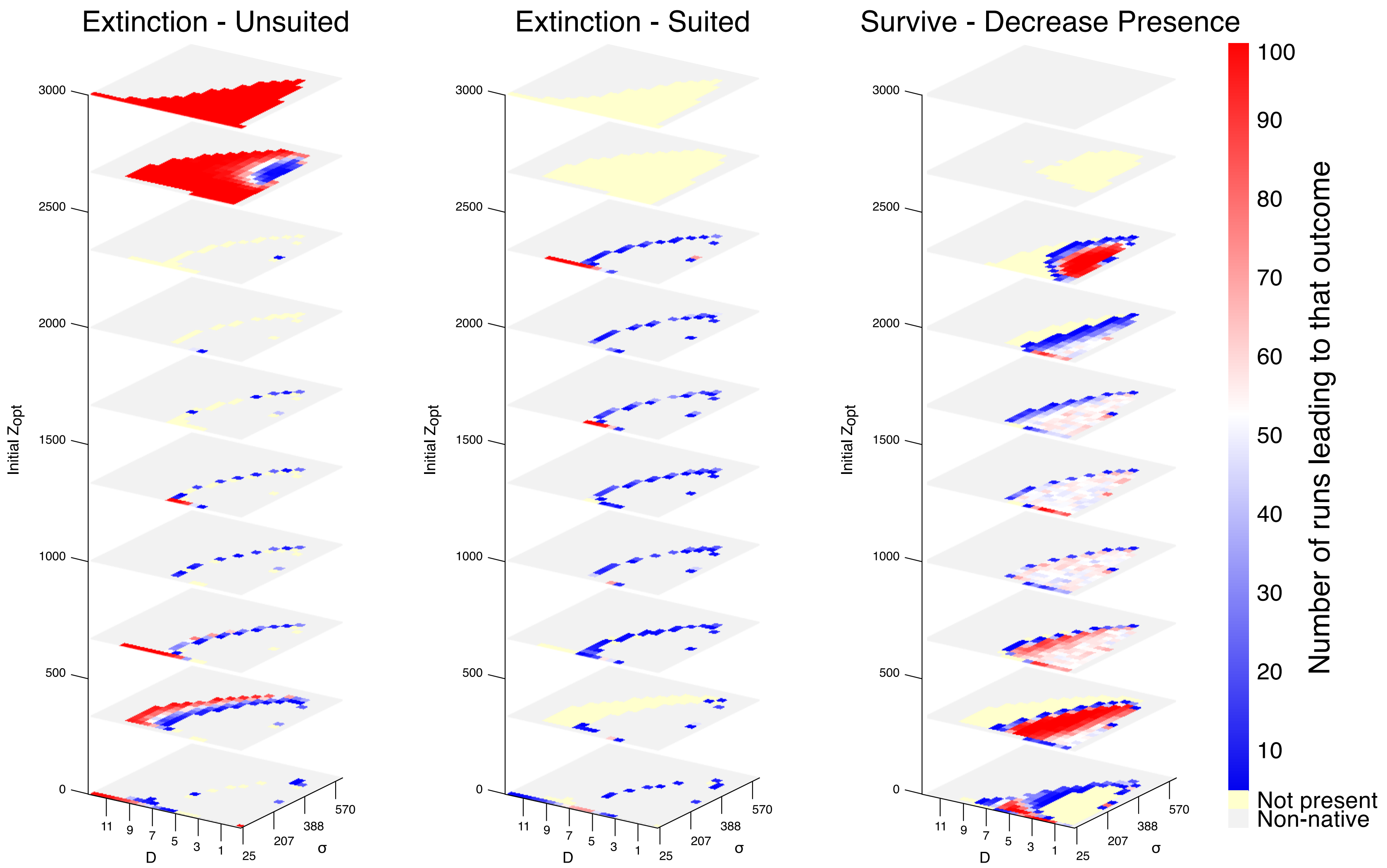

Fig S3.3. Intermediate results for the 'Roof' landscape. Number of runs leading to the fate 'Extinction unsuited' for each species (left), 'Extinction suited' (center) and 'Survive - Decrease Presence' (right).

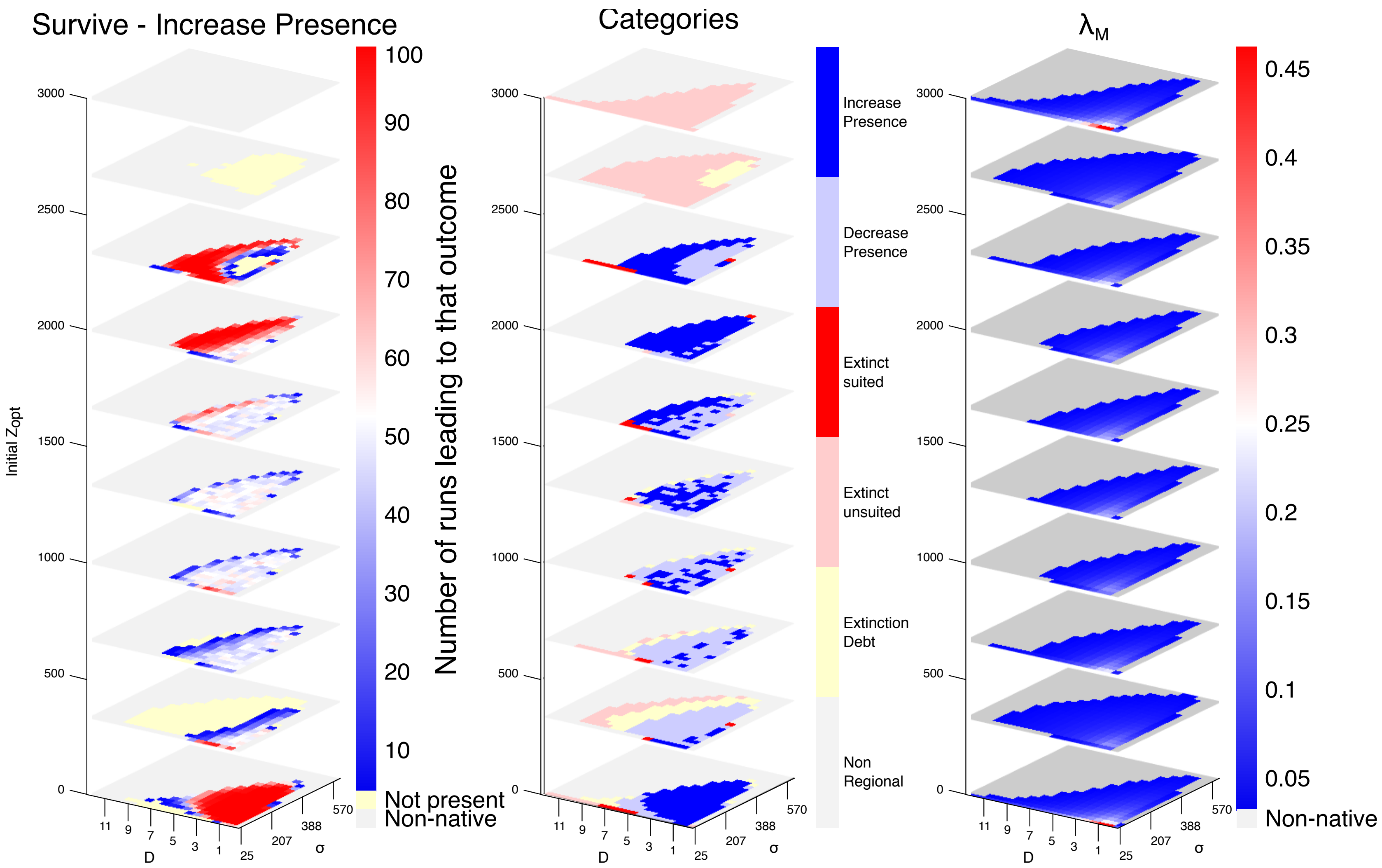

Fig S3.4. Intermediate results for the 'Roof' landscape. Number of runs leading to the fate 'Survive - Increase Presence' for each species (left). Classification of the most probable outcome for each species based on the previous figures (center). λ_M-value for each species, defining the threshold value of e/c for species survivability in the landscape.

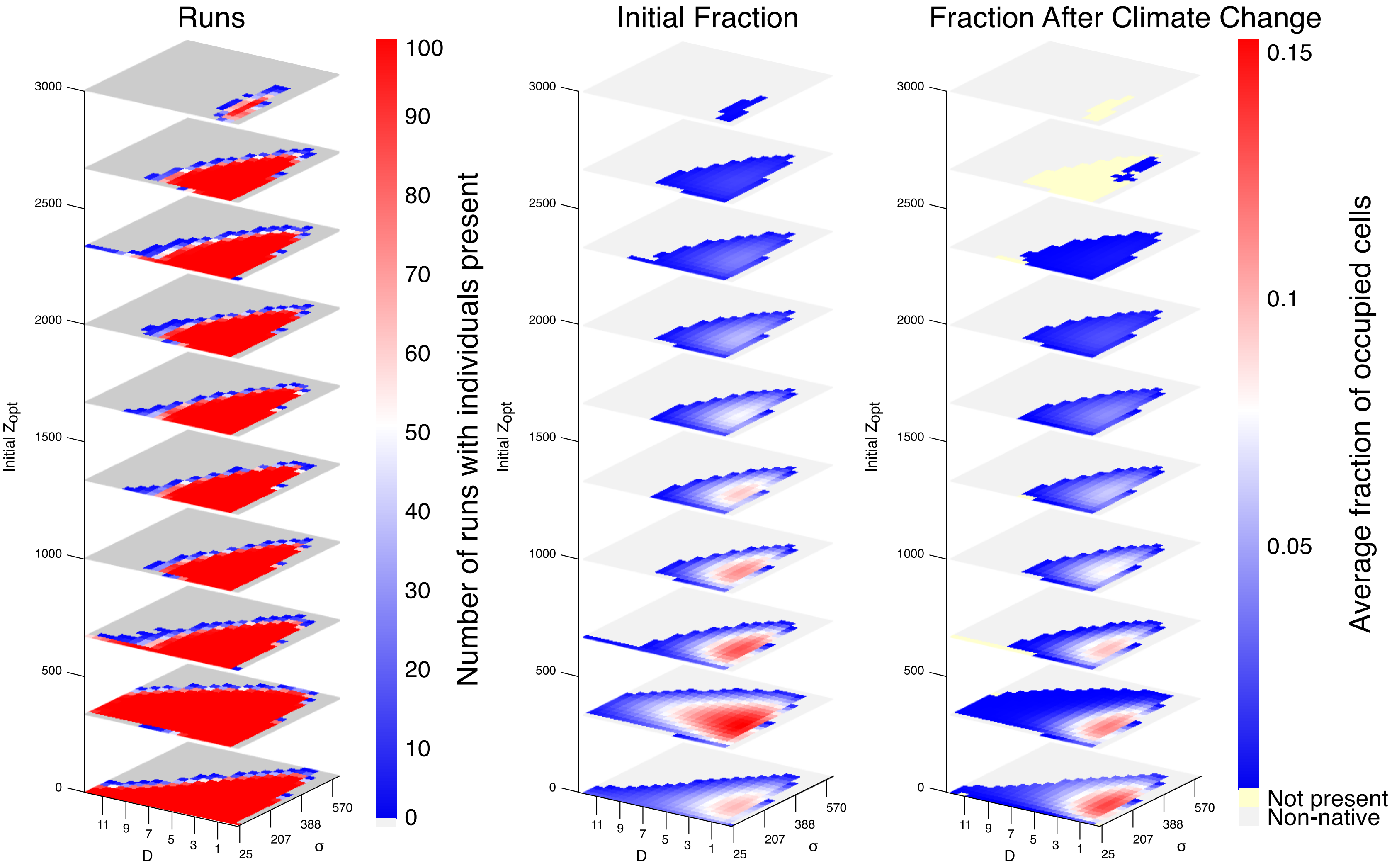

Fig S3.5. Same as figure S3.1 but for the Pyramid

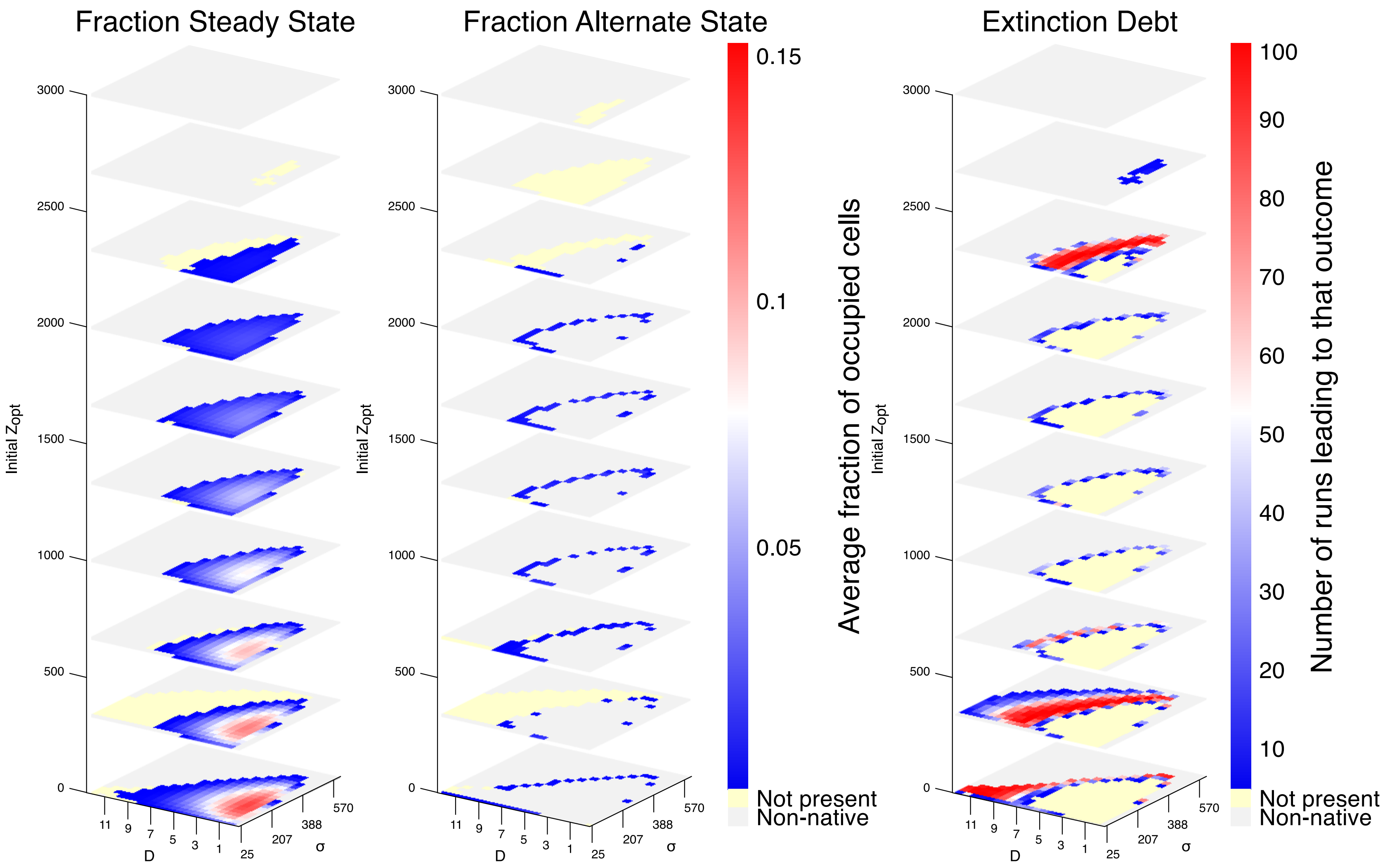

Fig S3.6. Same as figure S3.2 but for the Pyramid

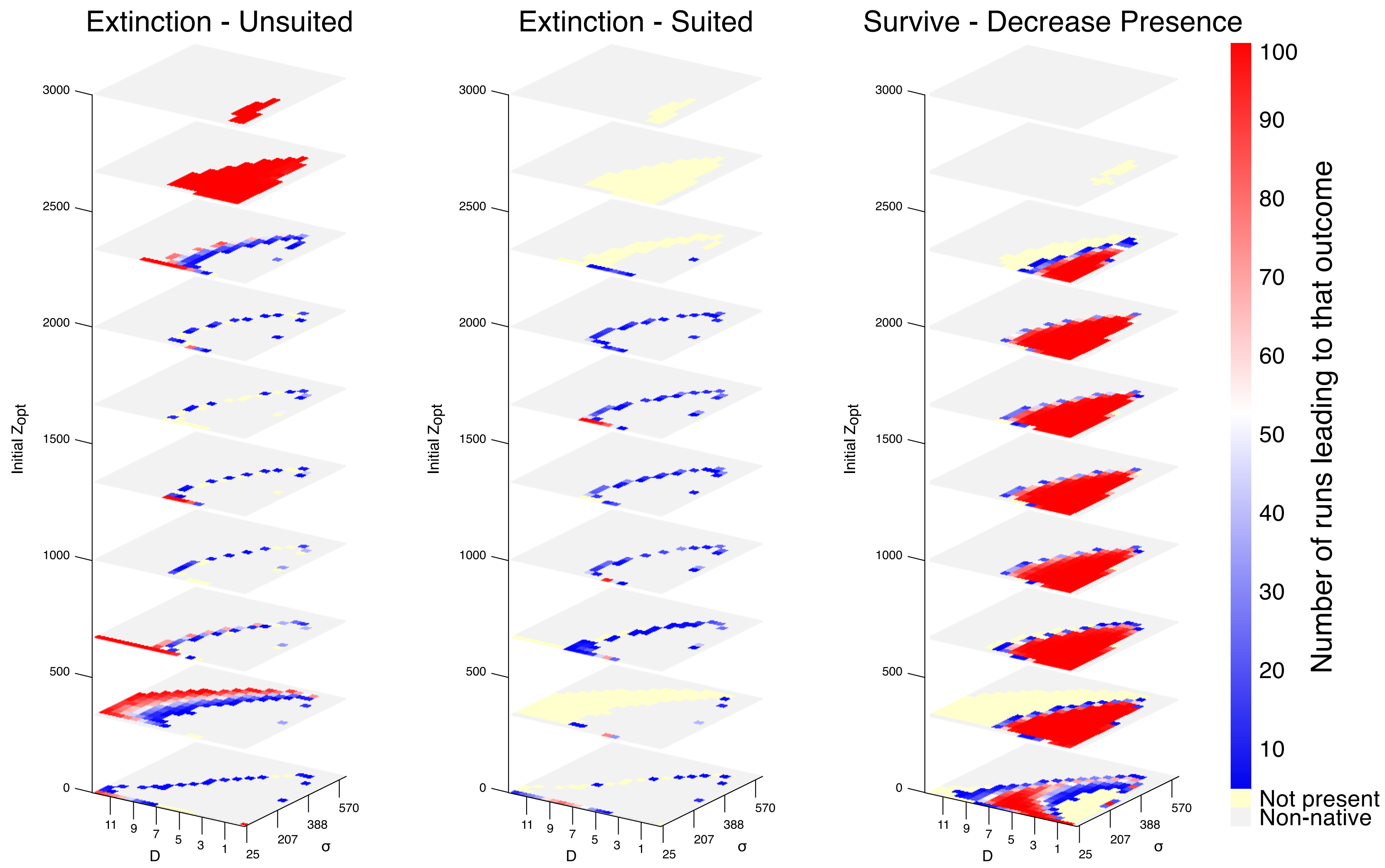

Fig S3.7. Same as figure S3.3 but for the Pyramid

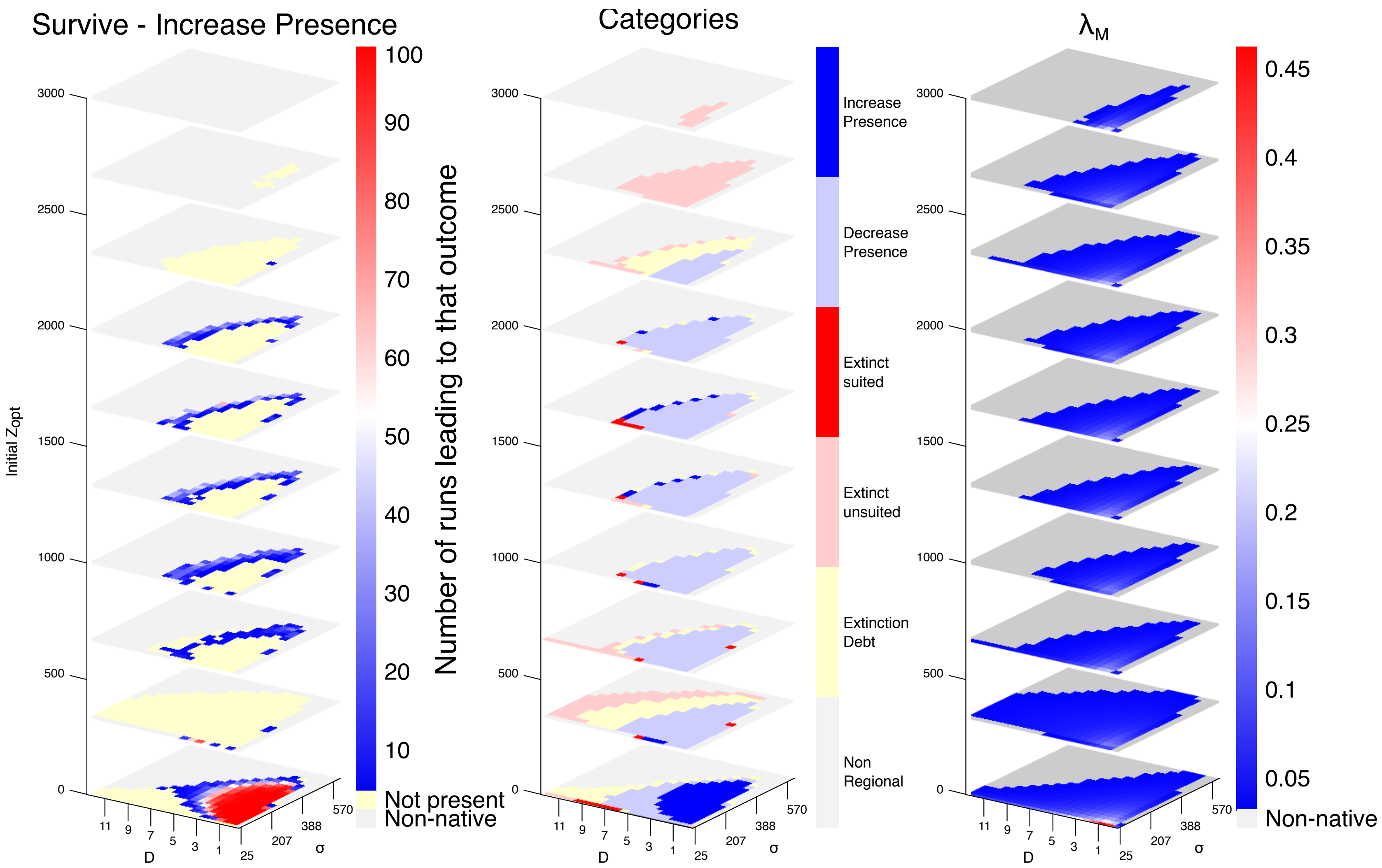

Fig S3.8. Same as figure S3.4 but for the Pyramid

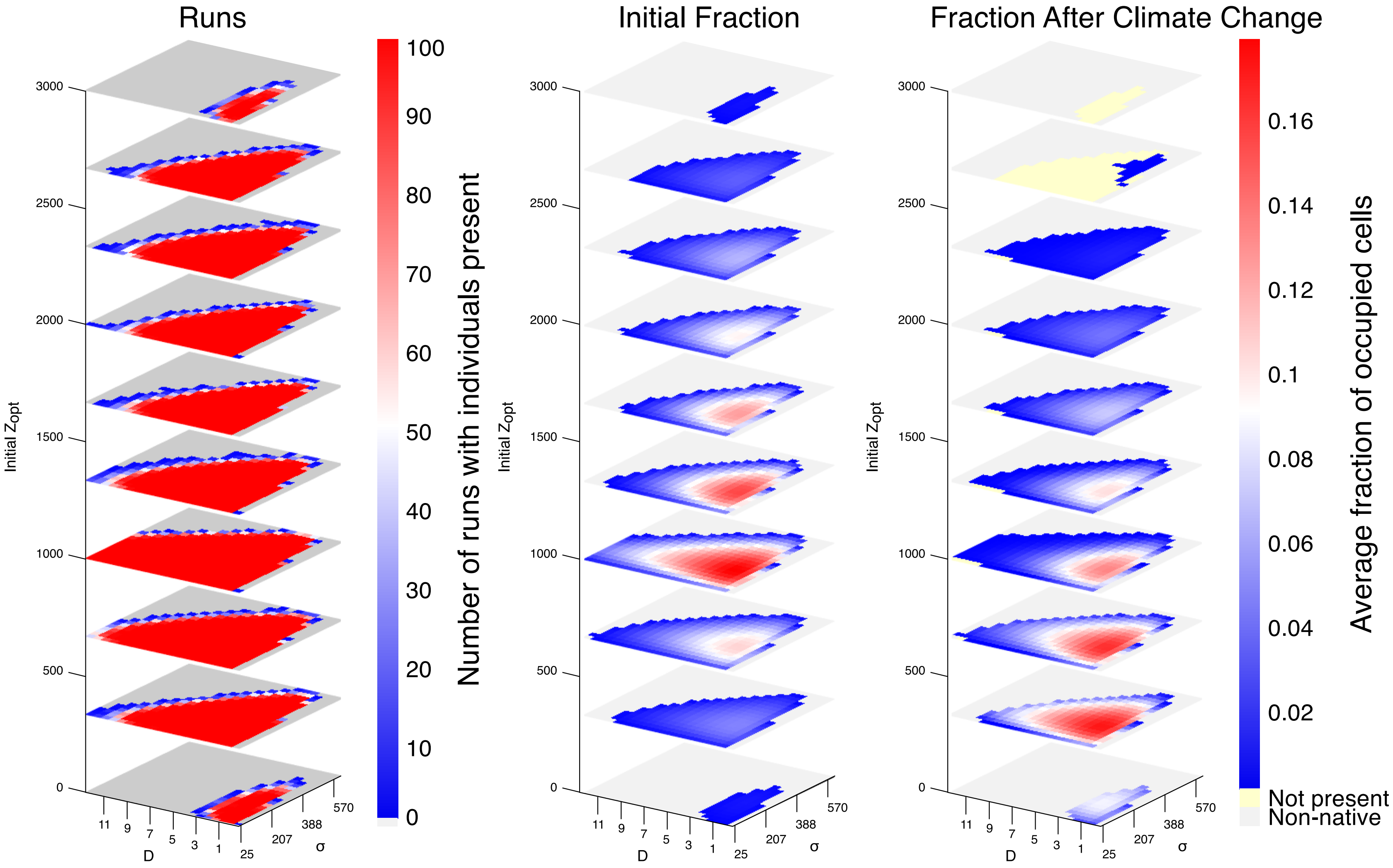

Fig S3.9. Same as figure S3.1 but for the Cone in a square

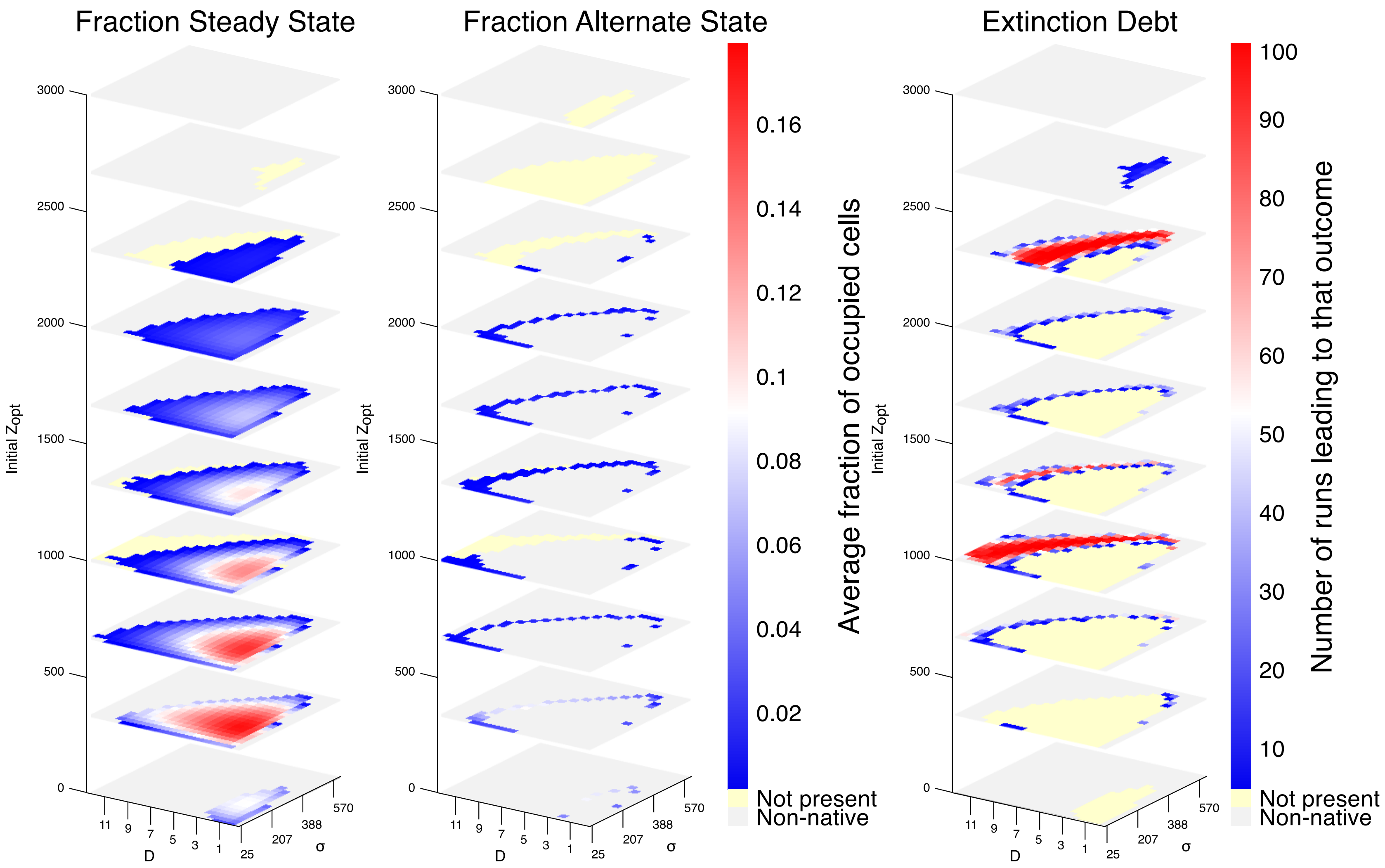

Fig S3.10. Same as figure S3.2 but for the Cone in a square

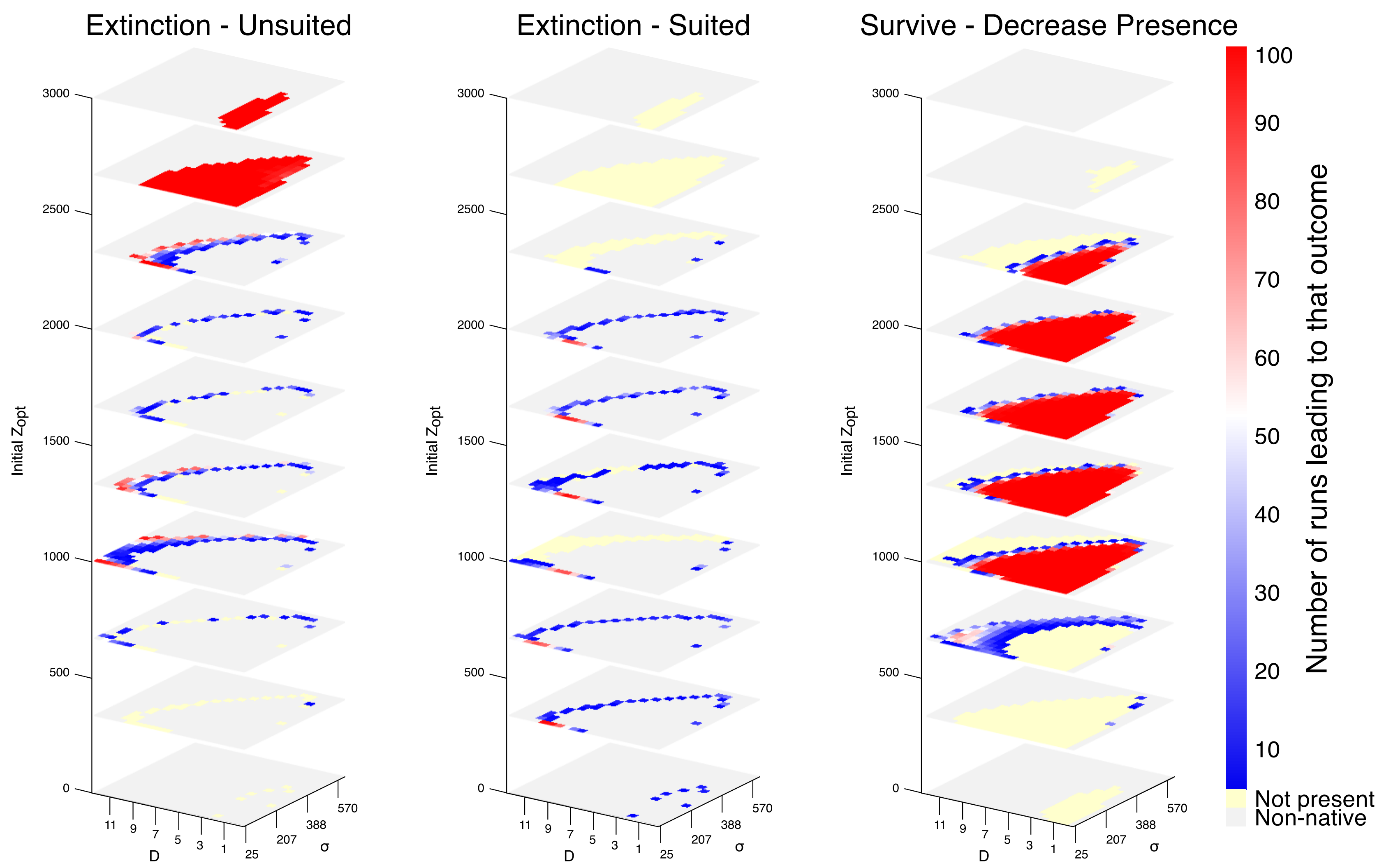

Fig S3.11. Same as figure S3.3 but for the Cone in a square

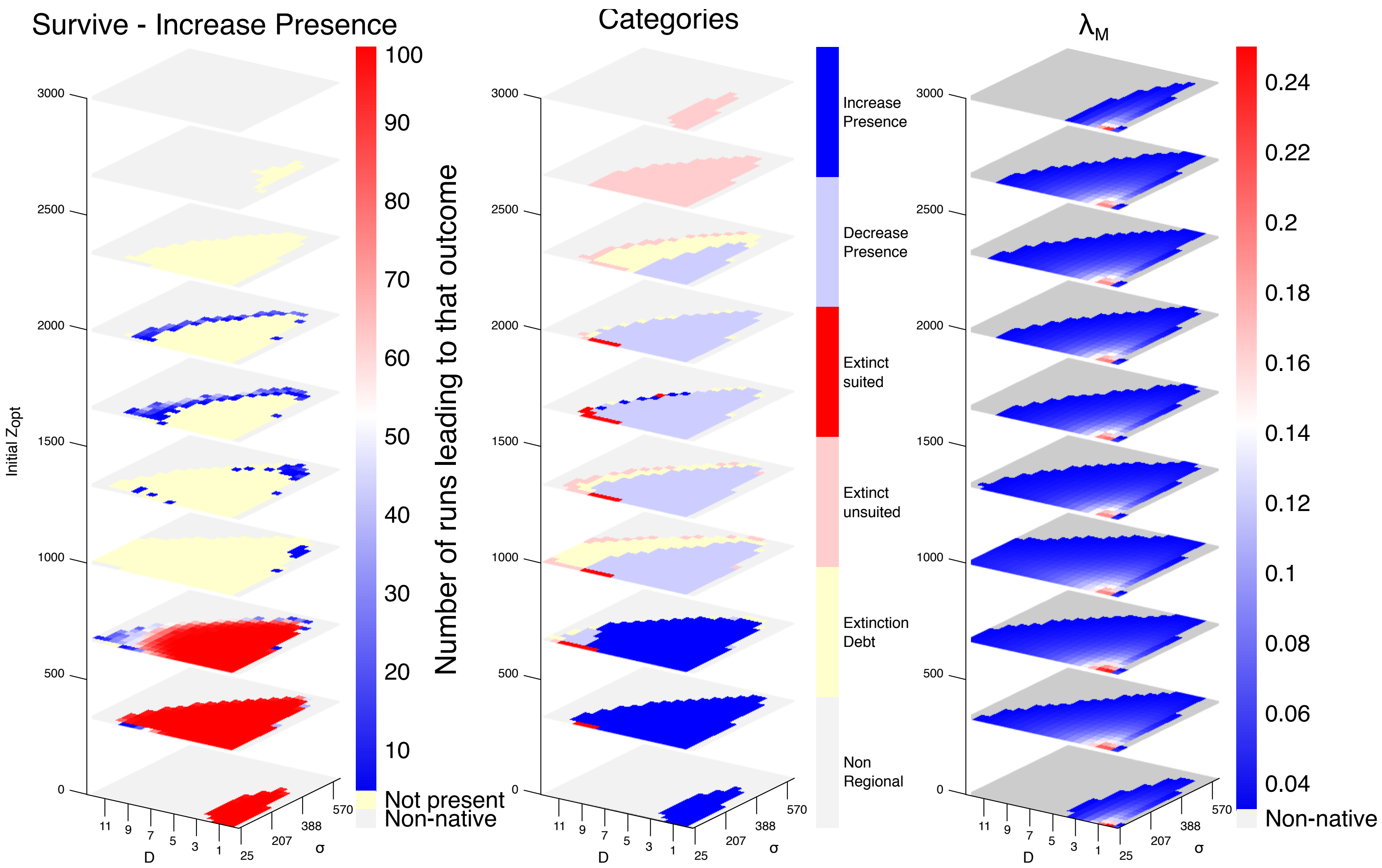

Fig S3.12. Same as figure S3.4 but for the Cone in a square

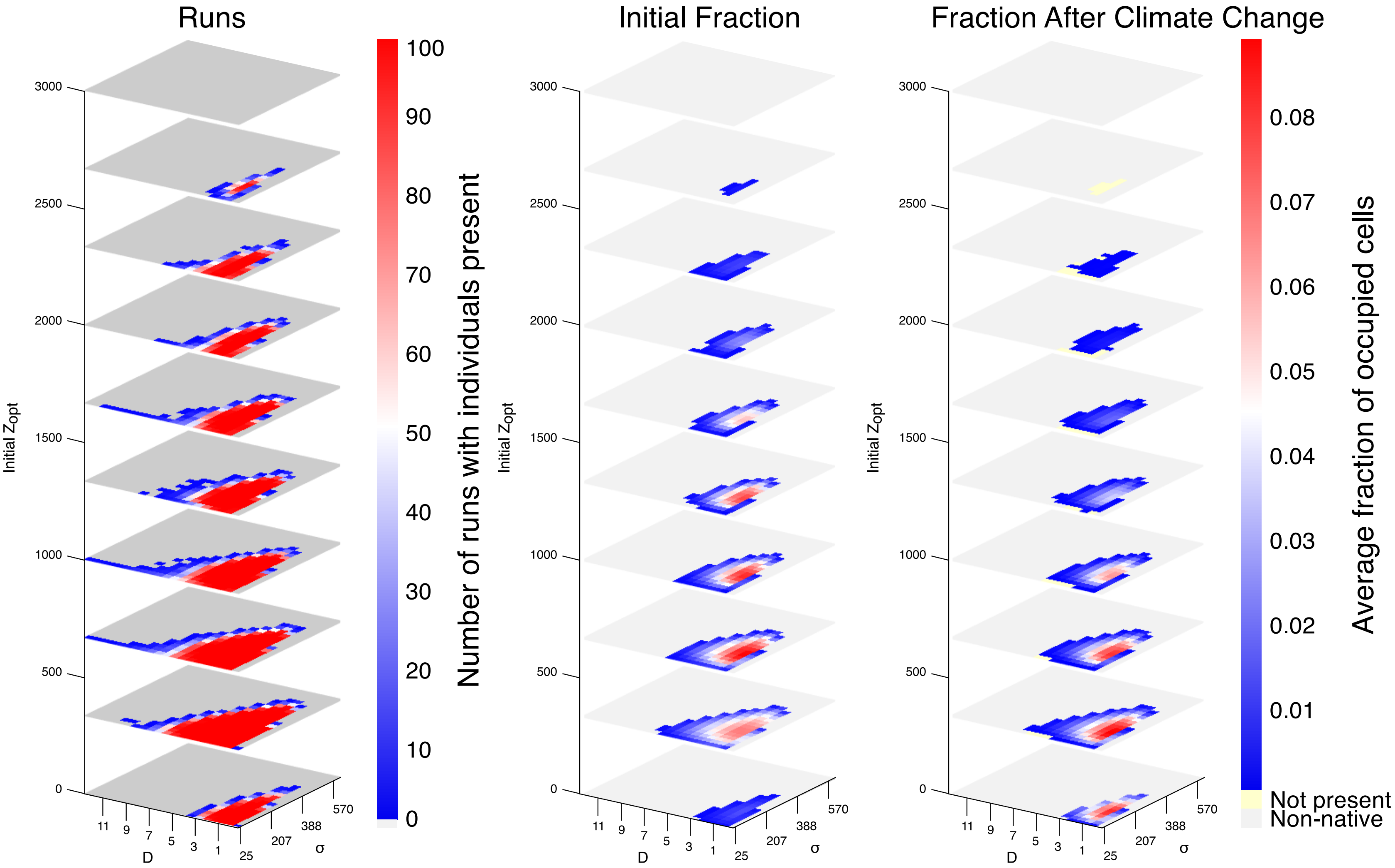

Fig S3.13. Same as figure S3.1 but for the OCN

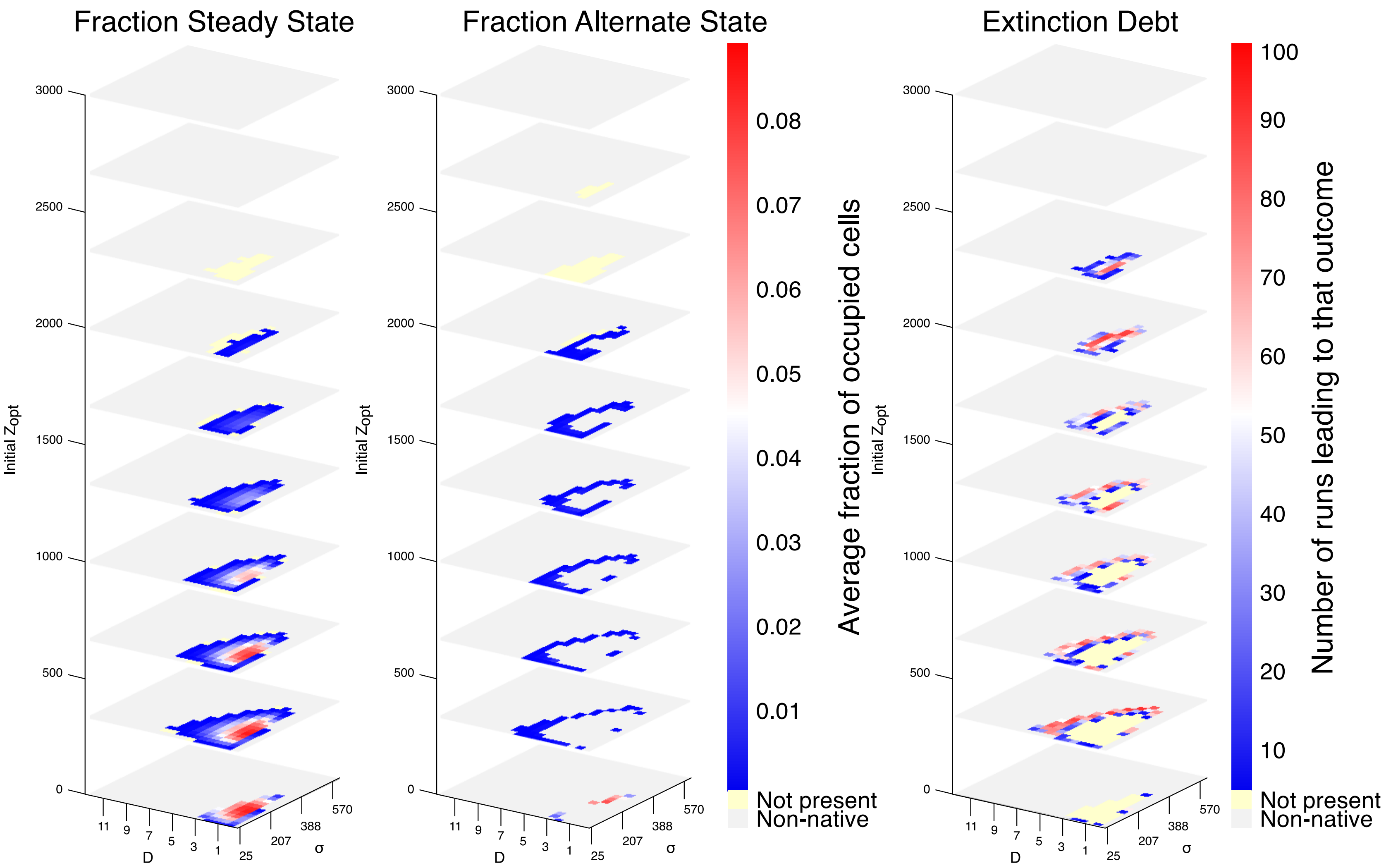

Fig S3.14. Same as figure S3.2 but for the OCN

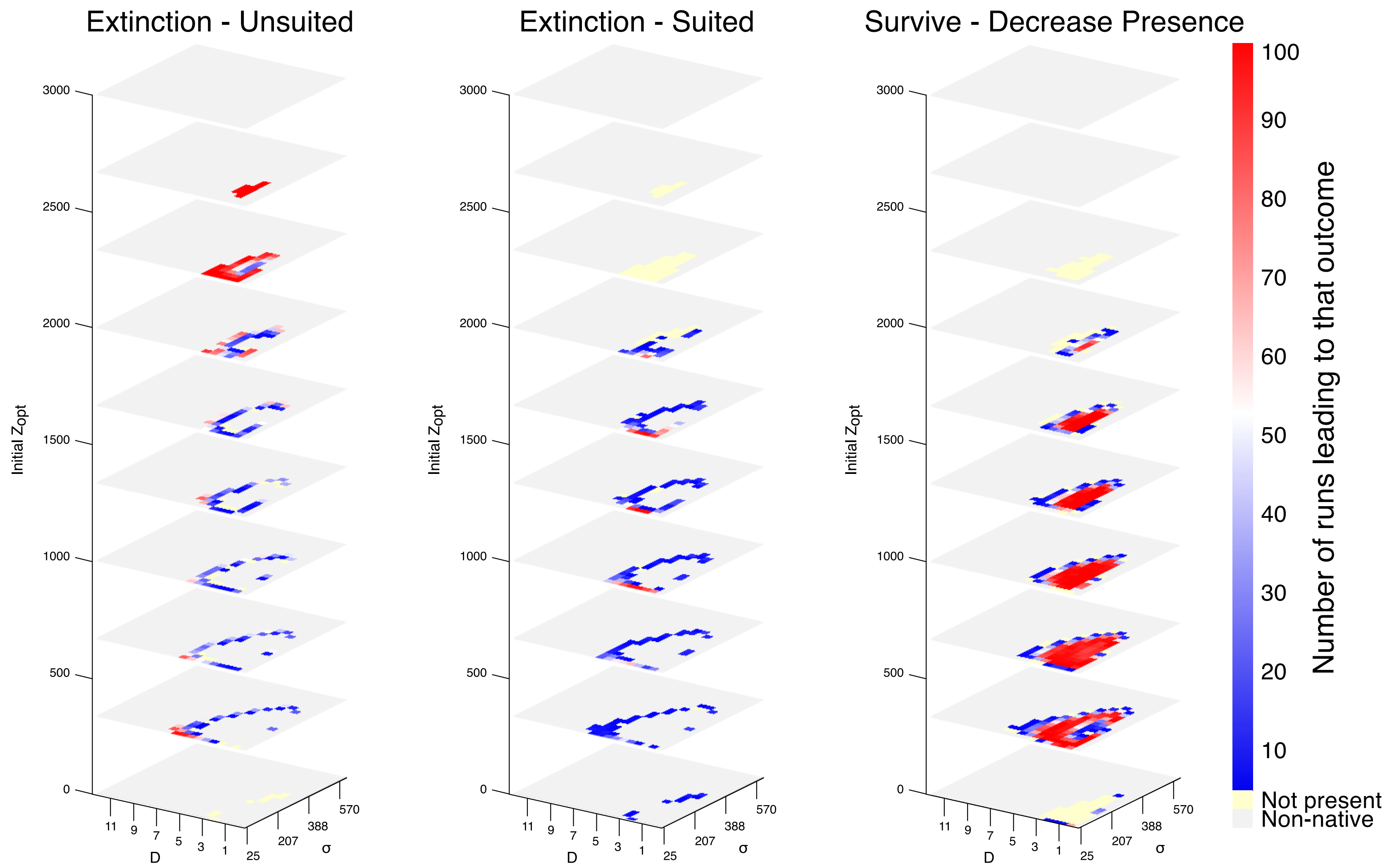

Fig S3.15. Same as figure S3.3 but for the OCN

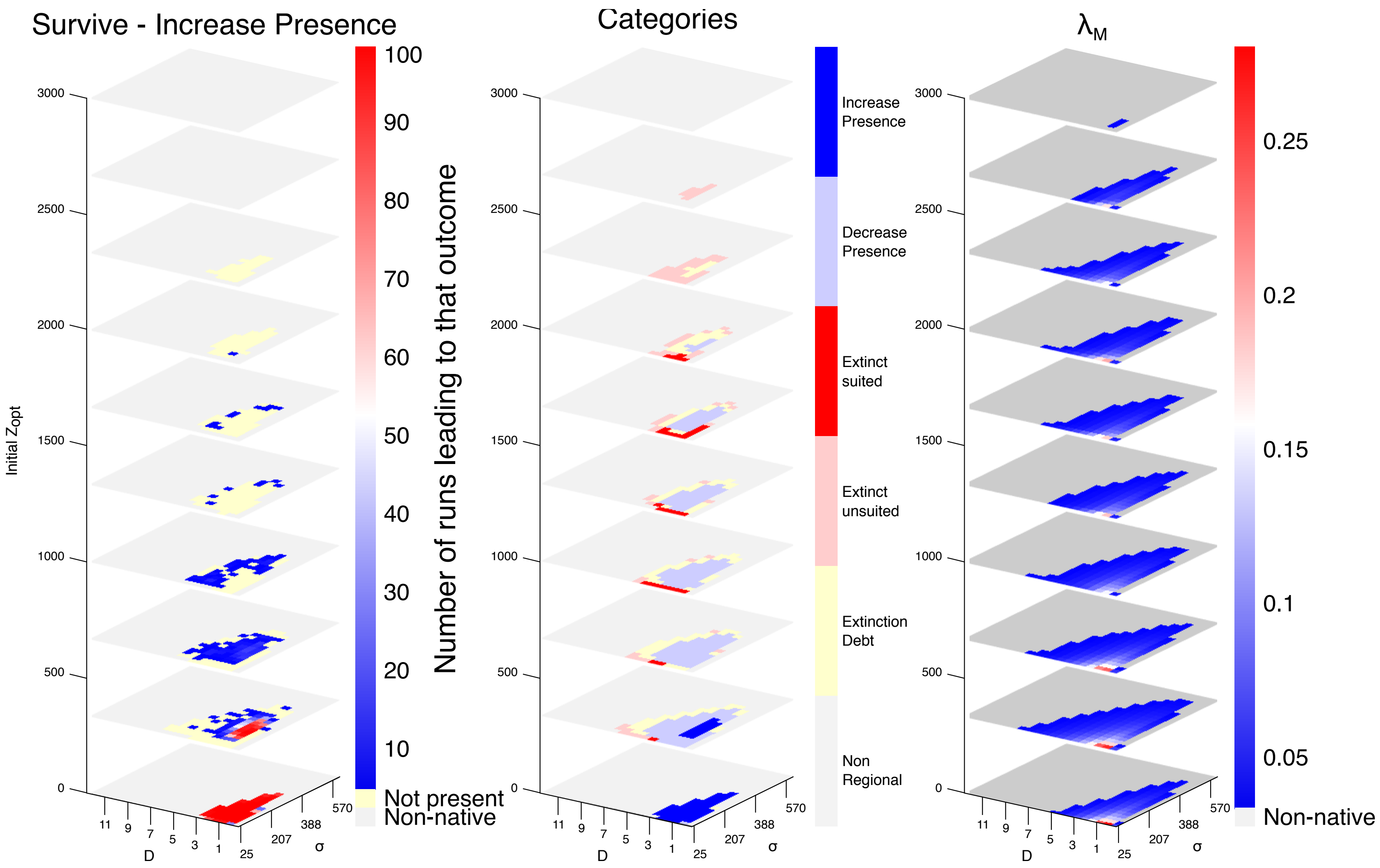

Fig S3.16. Same as figure S3.4 but for the OCN

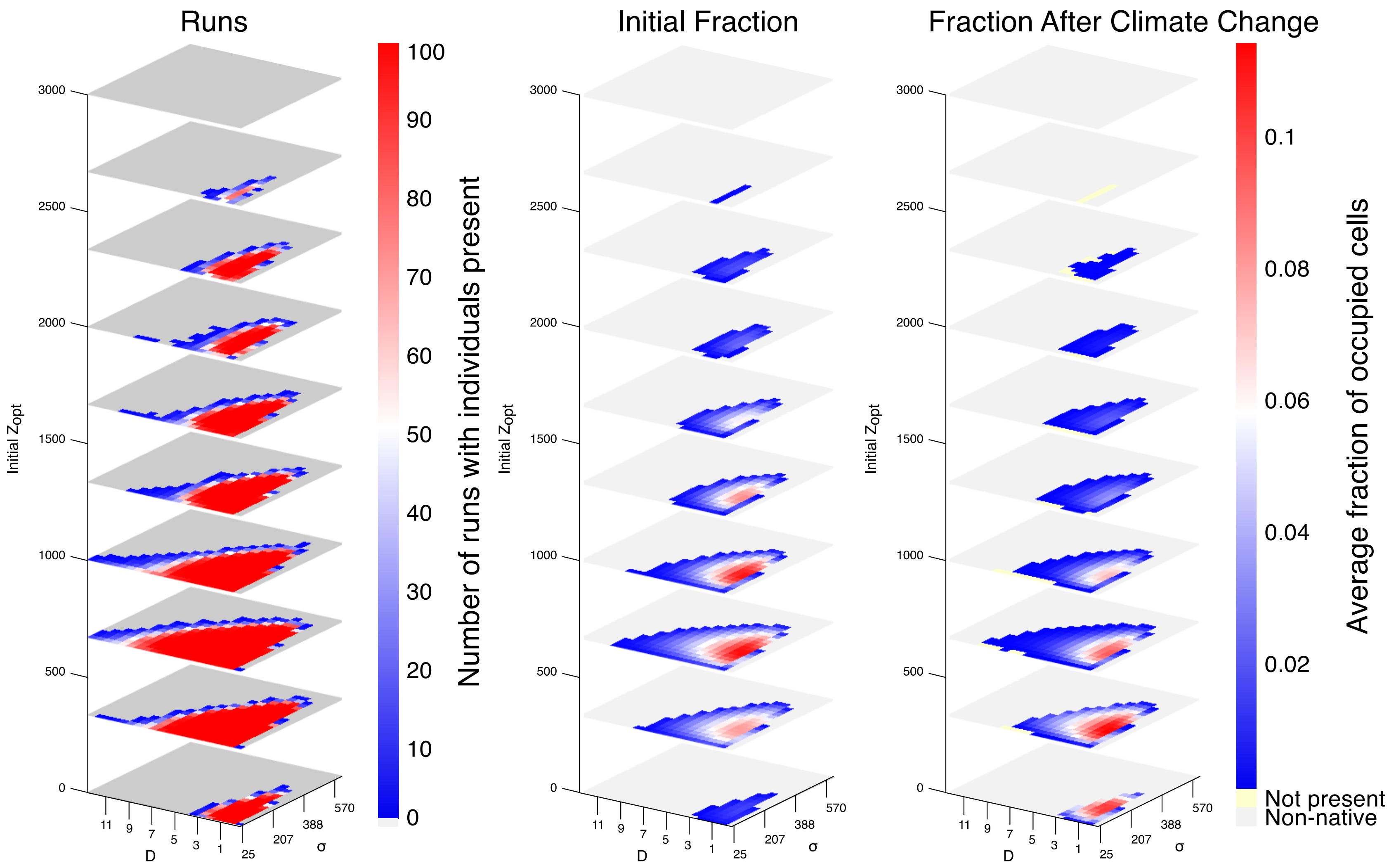

Fig S3.17. Same as figure S3.1 but for the OCN1

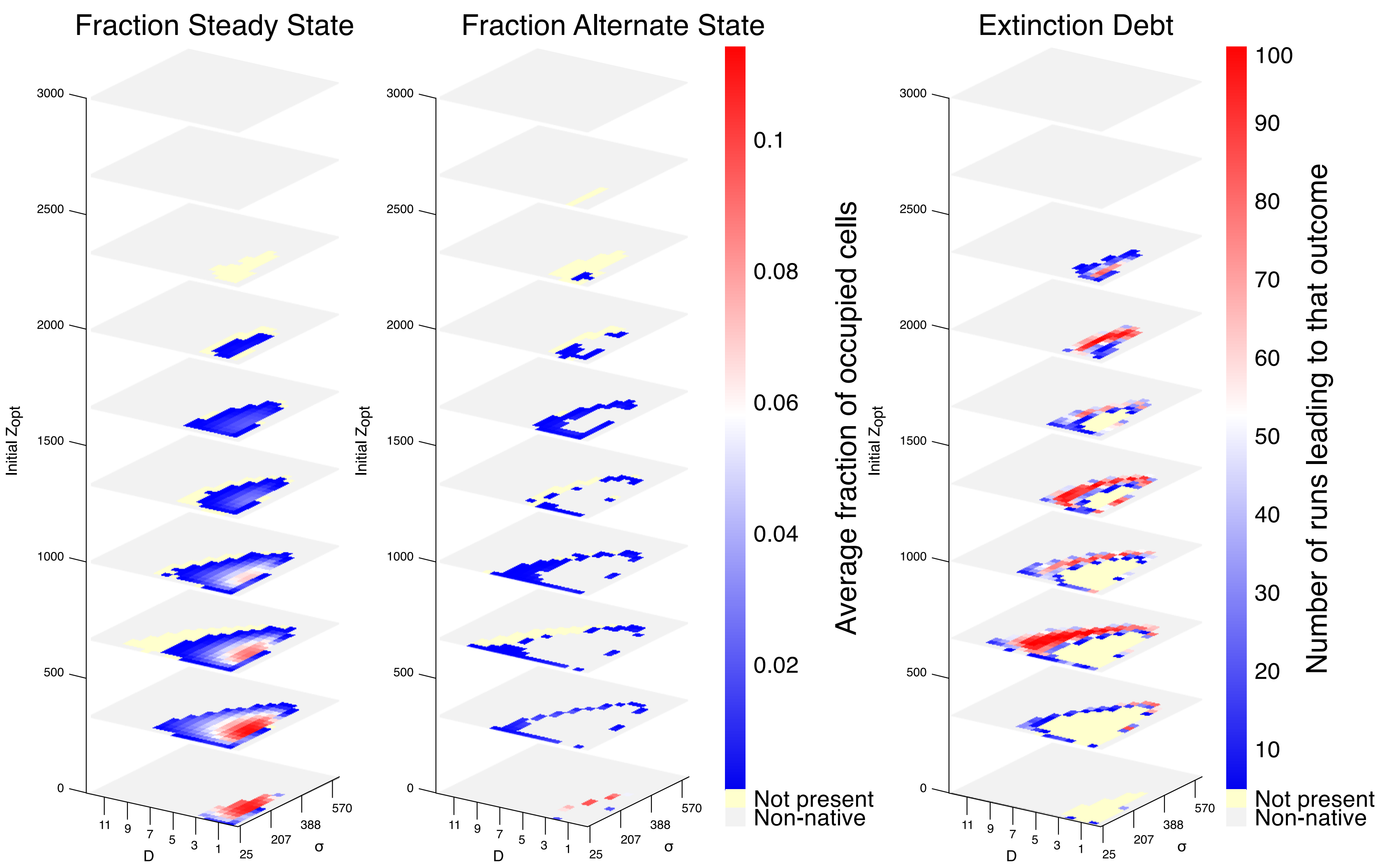

Fig S3.18. Same as figure S3.2 but for the OCN1

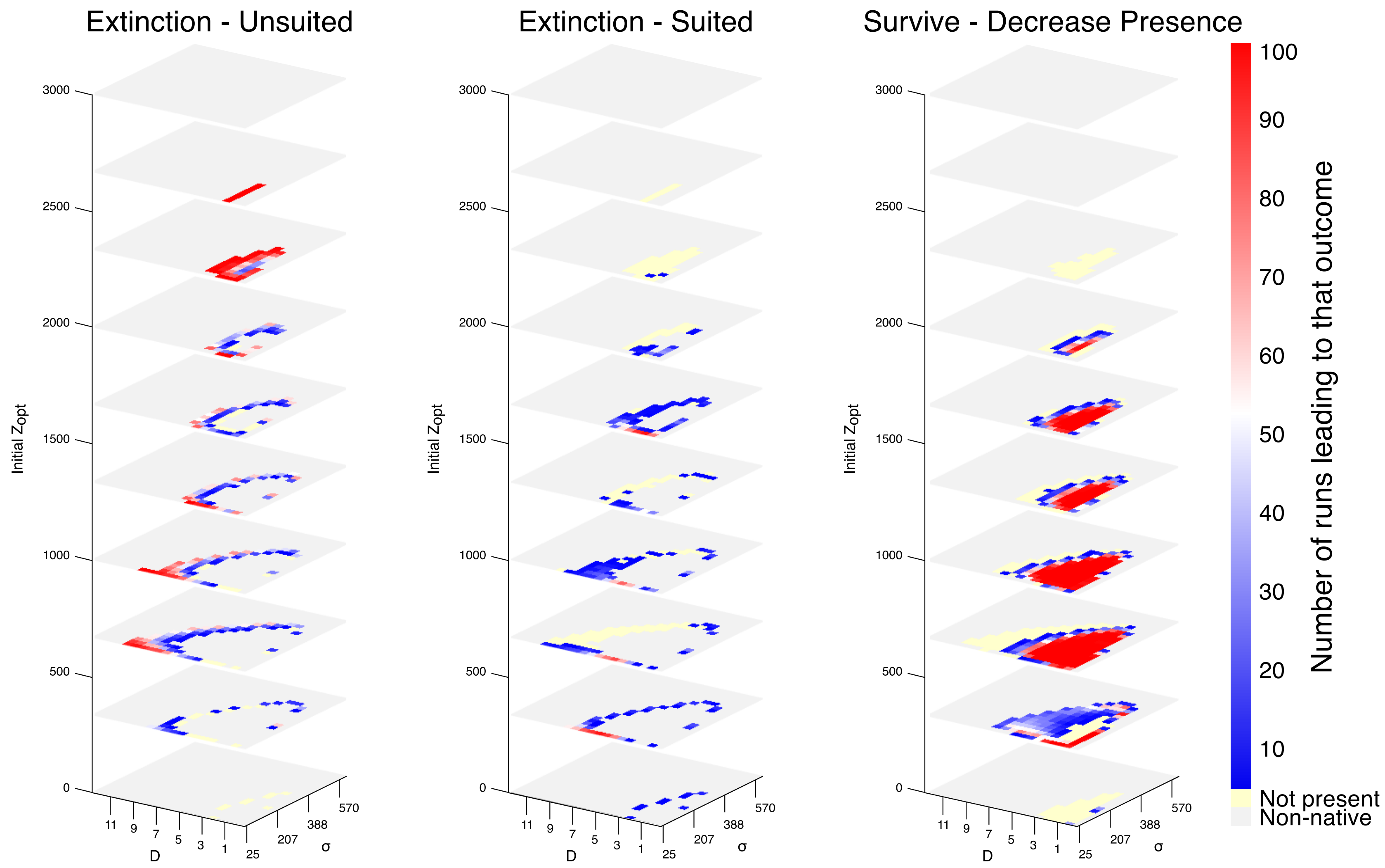

Fig S3.19. Same as figure S3.2 but for the OCN1

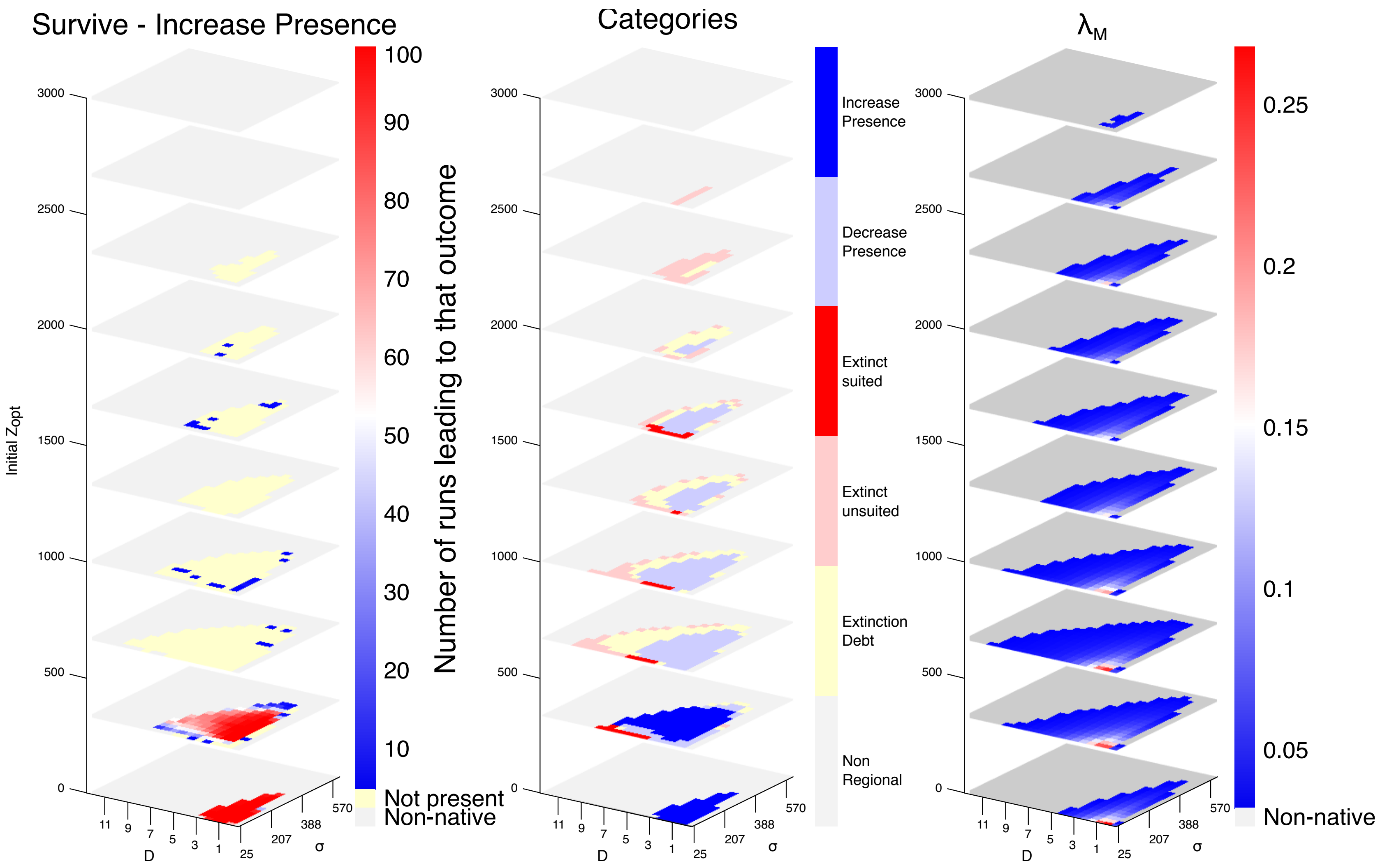

Fig S3.20. Same as figure S3.2 but for the OCN1

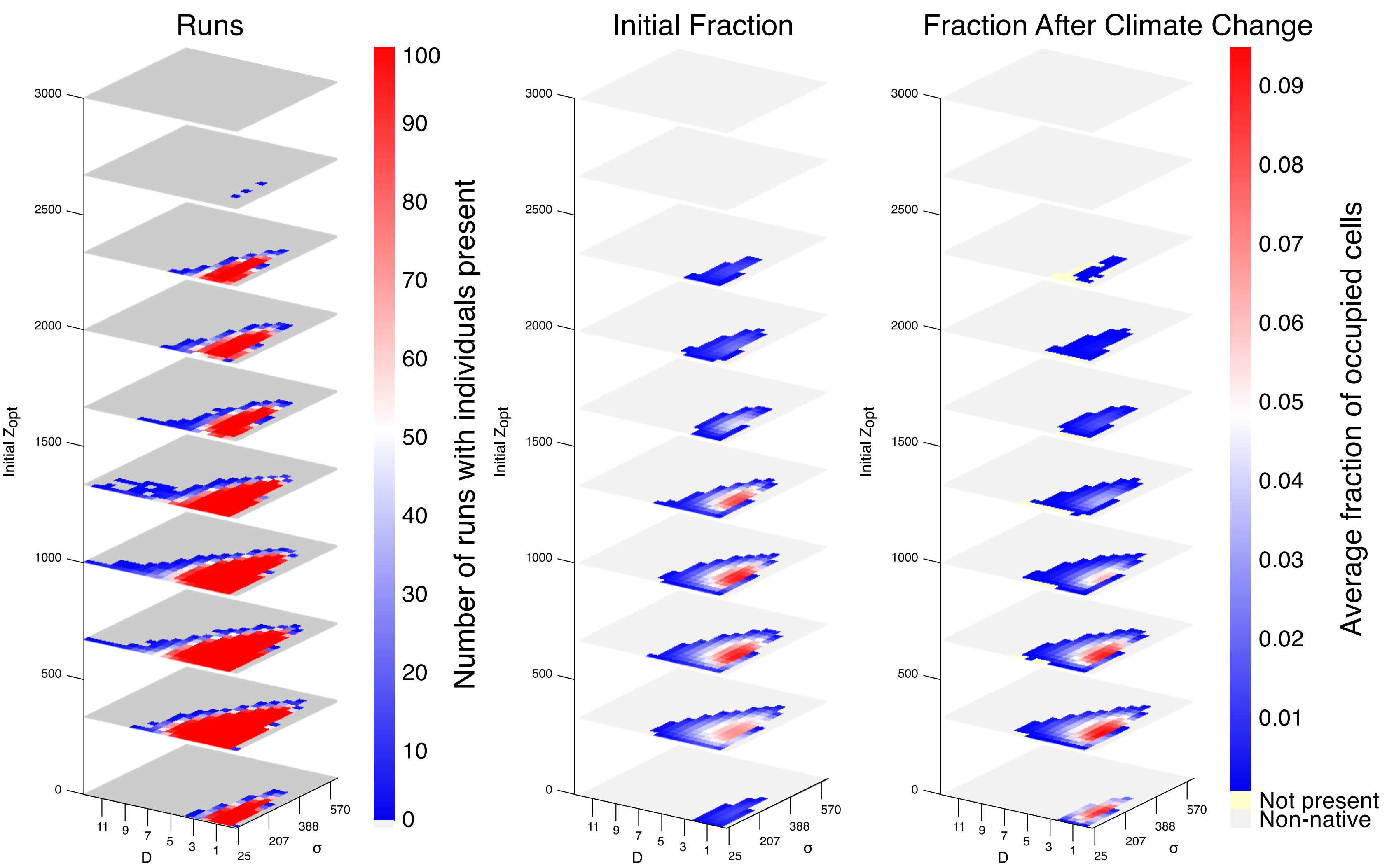

Fig S3.21. Same as figure S3.1 but for the OCN2

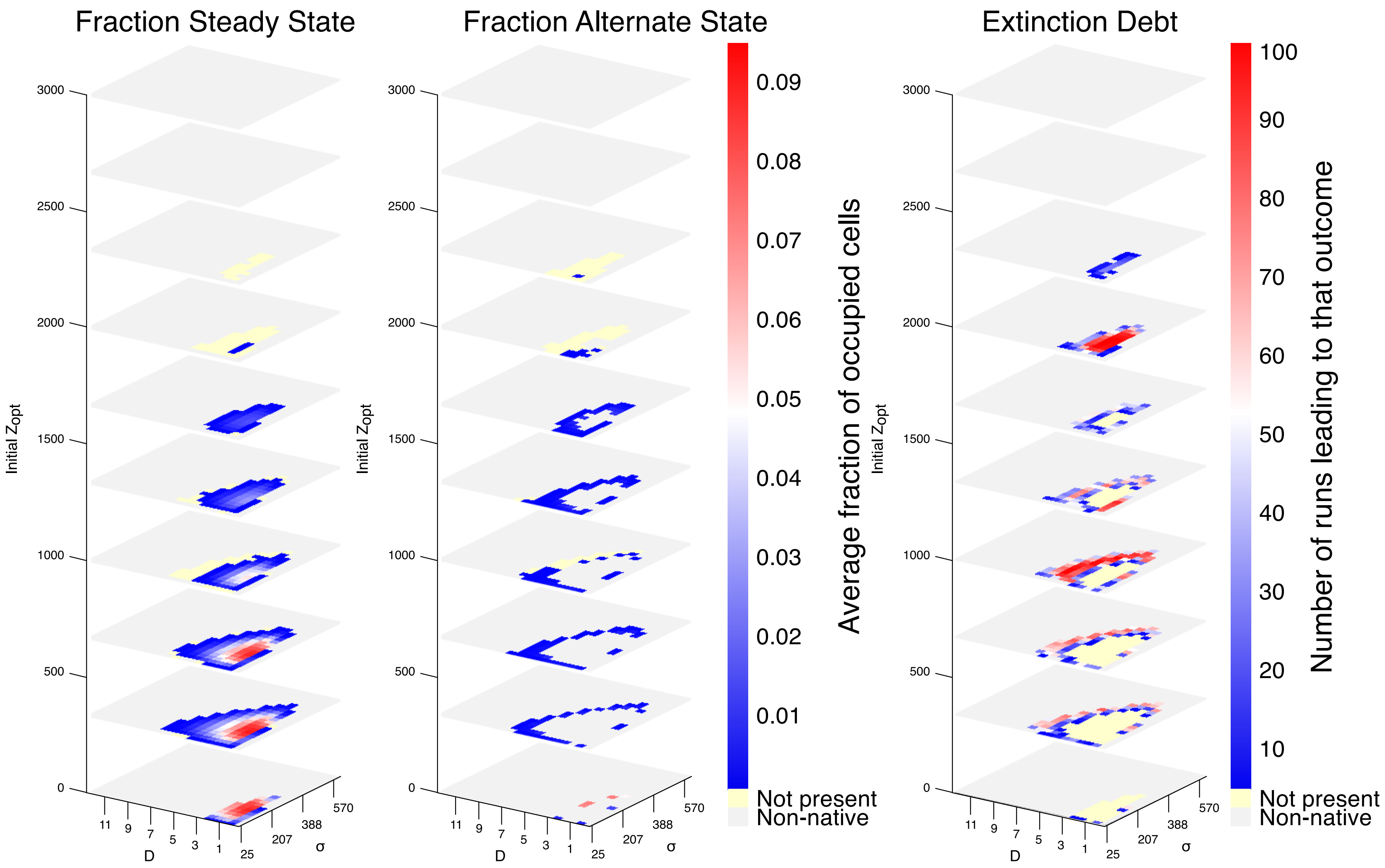

Fig S3.22. Same as figure S3.2 but for the OCN2

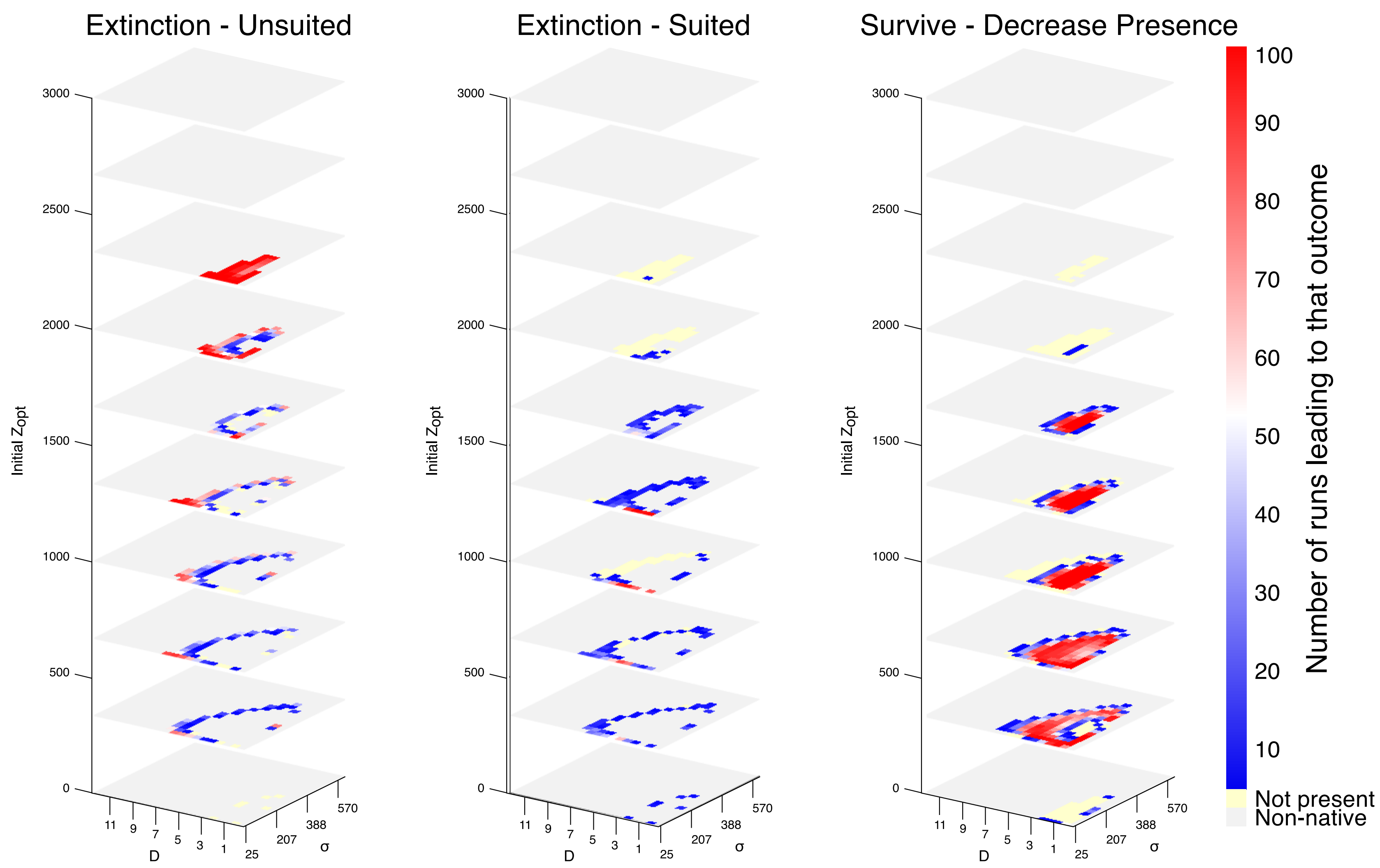

Fig S3.23. Same as figure S3.3 but for the OCN2

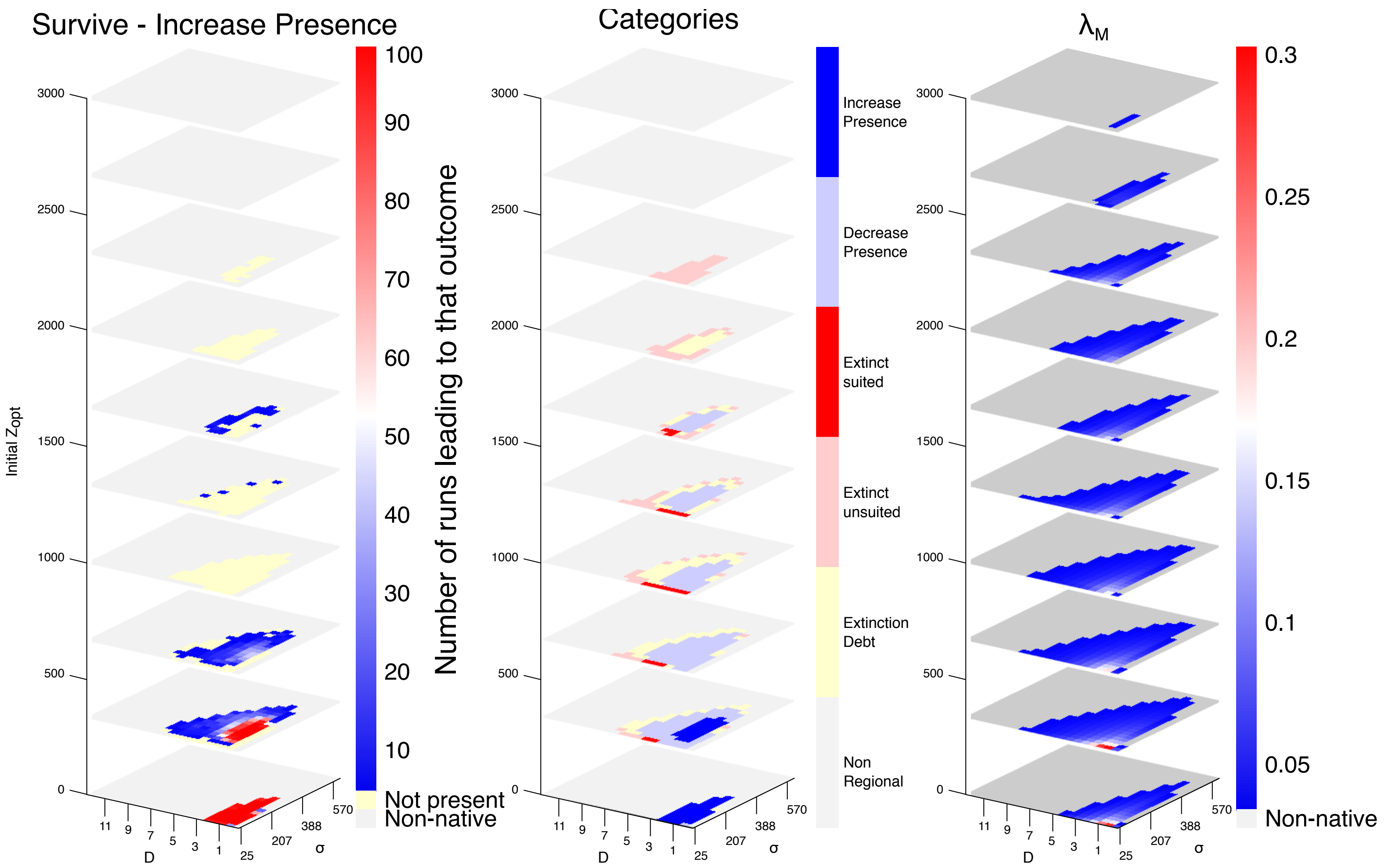

Fig S3.24. Same as figure S3.4 but for the OCN2

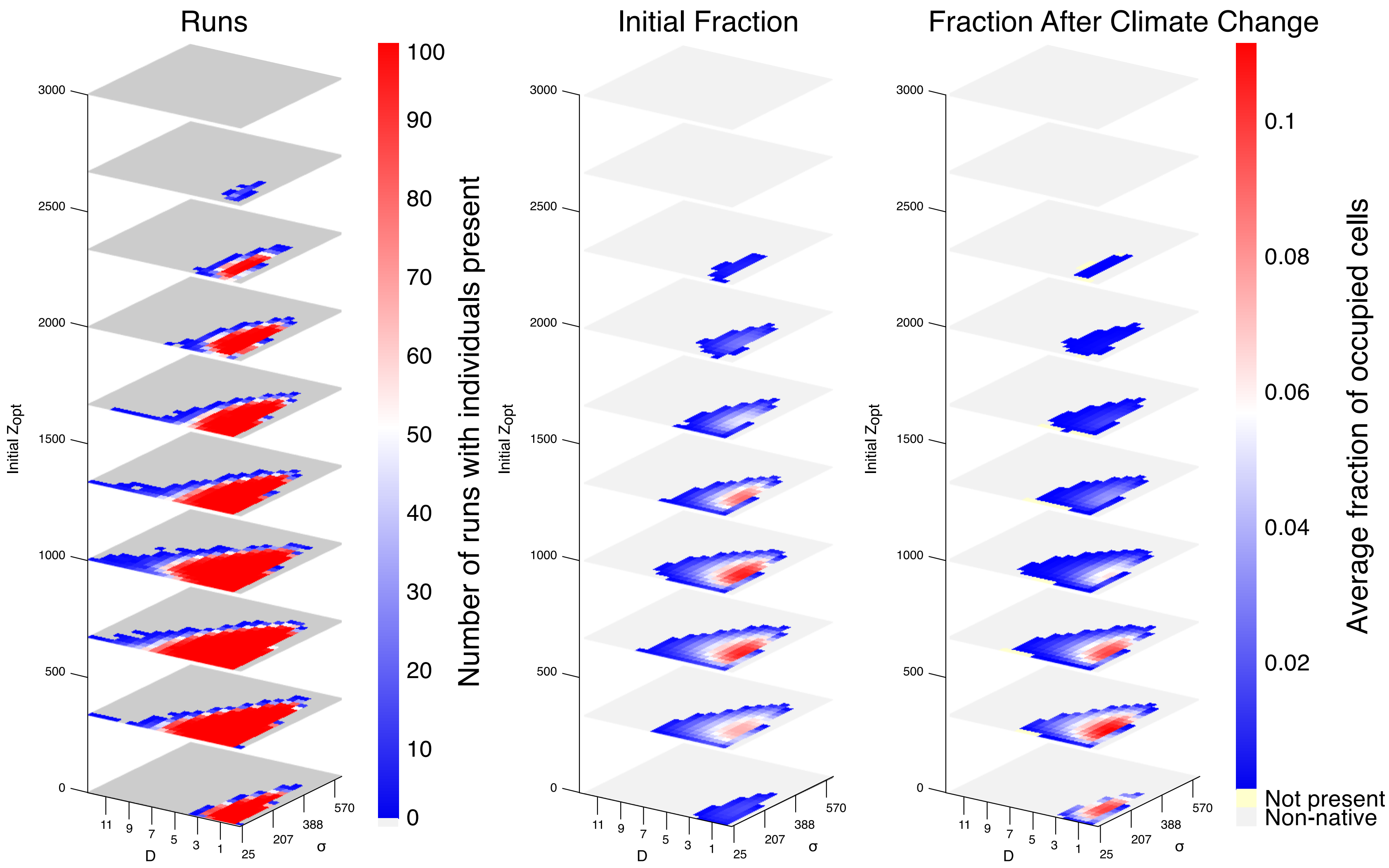

Fig S3.25. Same as figure S3.1 but for the OCN3

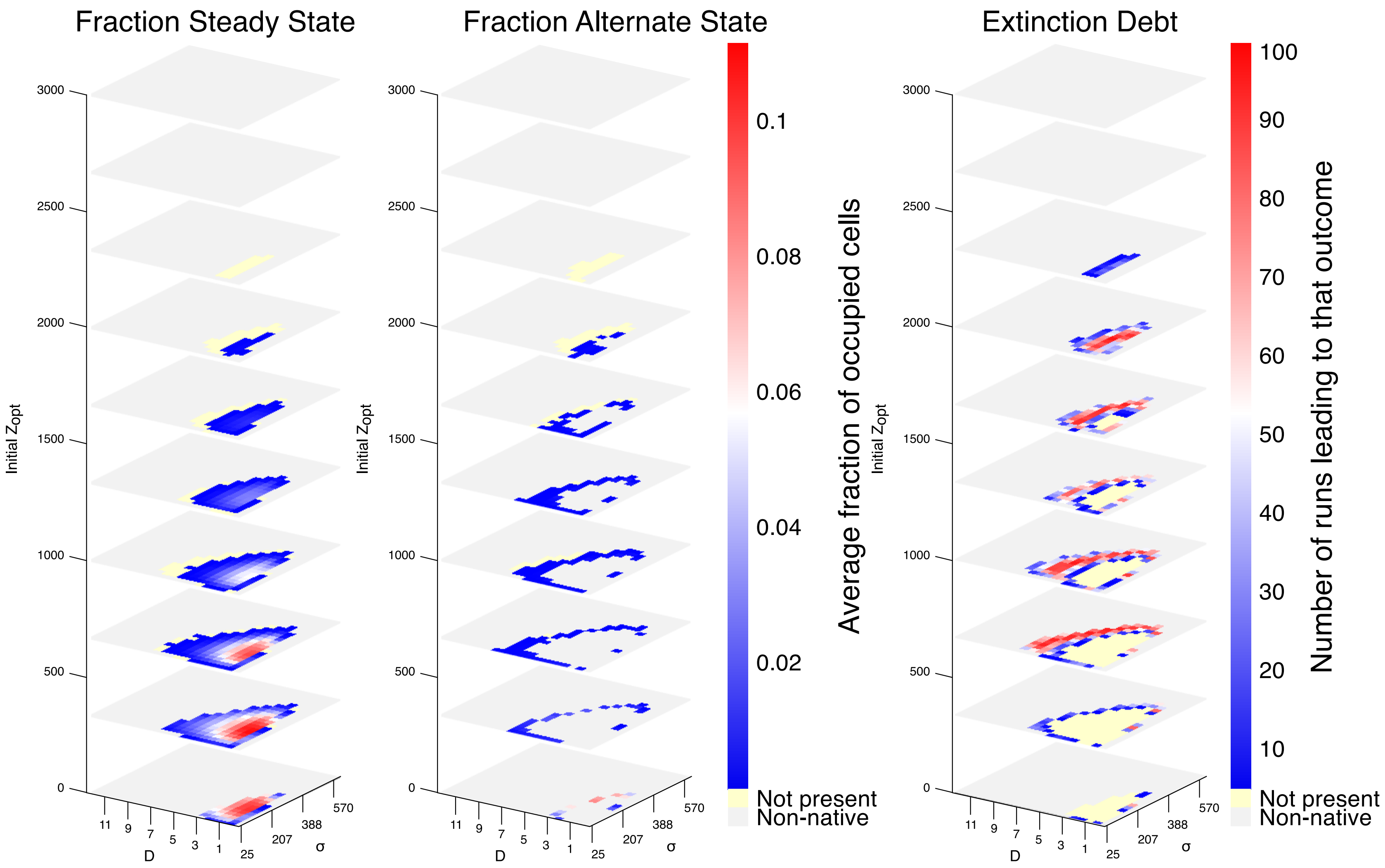

Fig S3.26. Same as figure S3.2 but for the OCN3

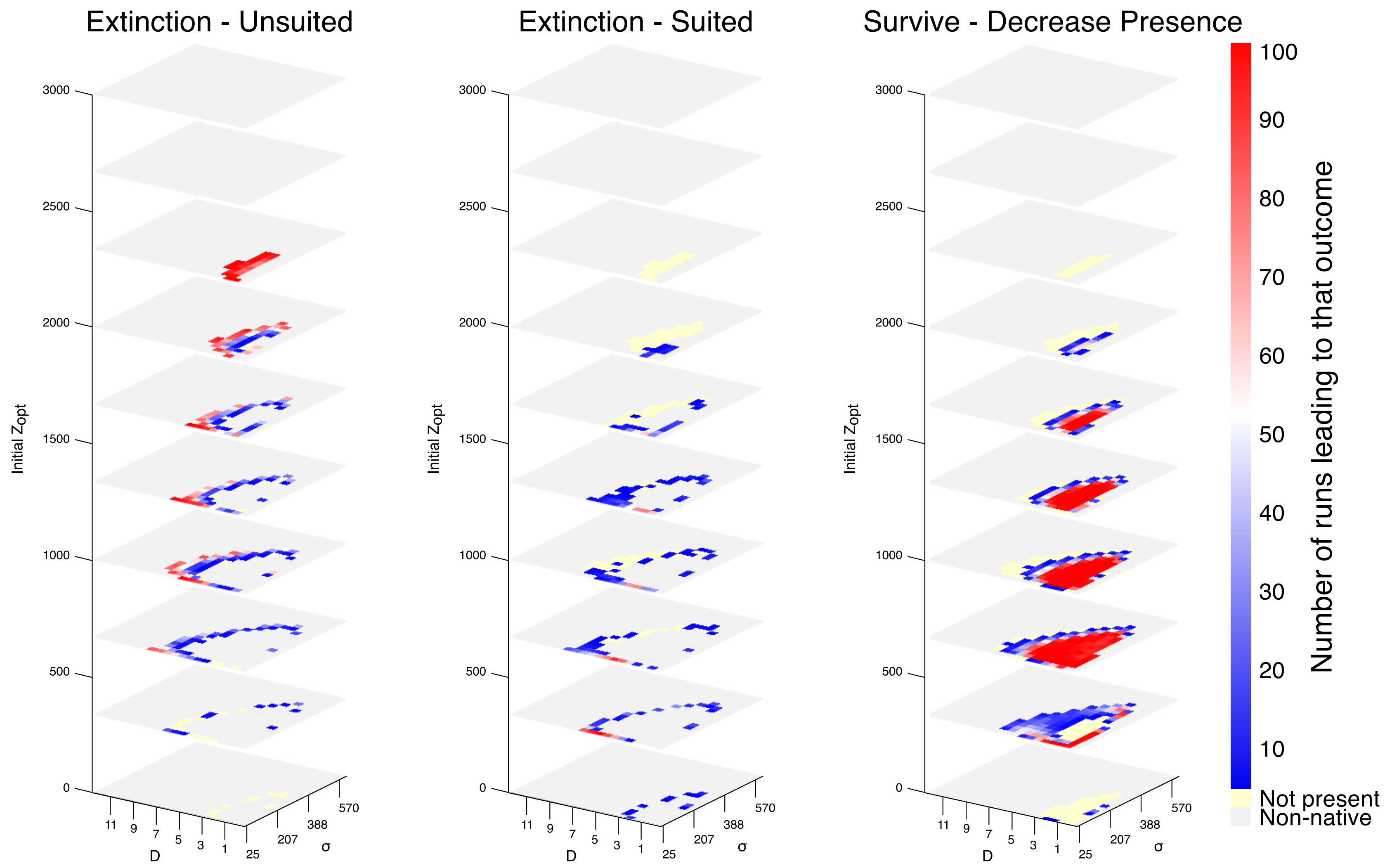

Fig S3.27. Same as figure S3.3 but for the OCN3

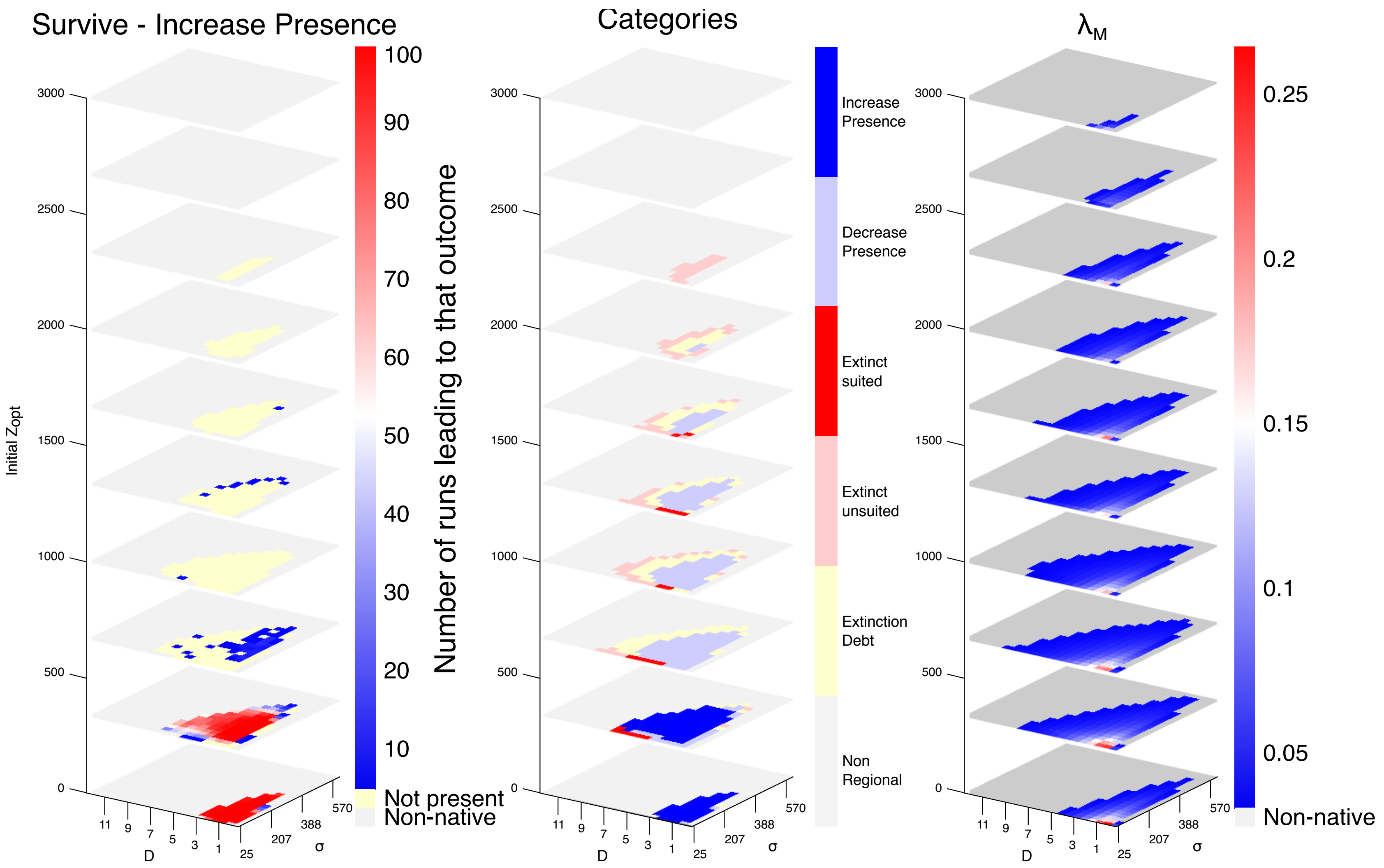

Fig S3.28. Same as figure S3.4 but for the OCN3

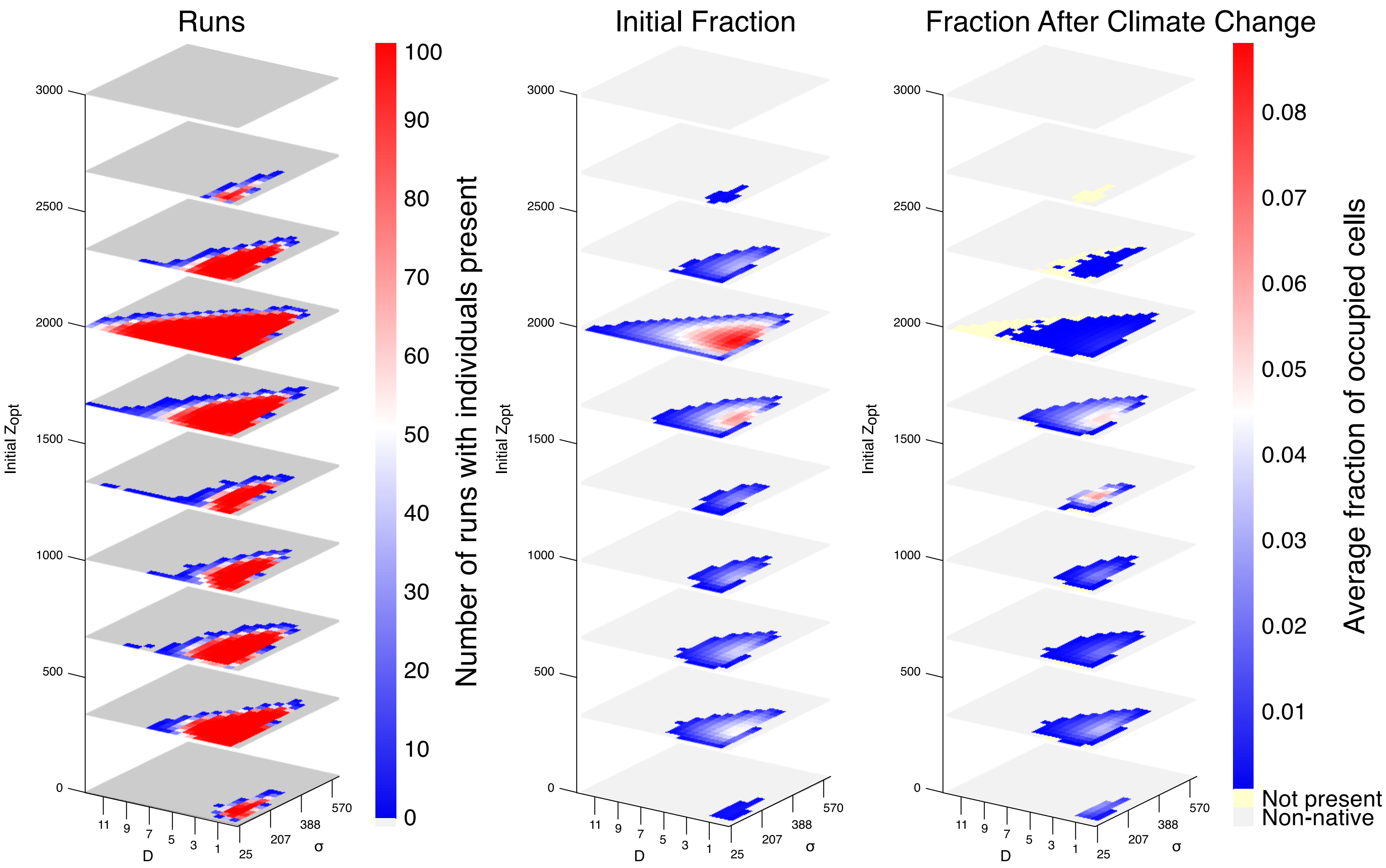

Fig S3.29. Same as figure S3.1 but for the GPNP

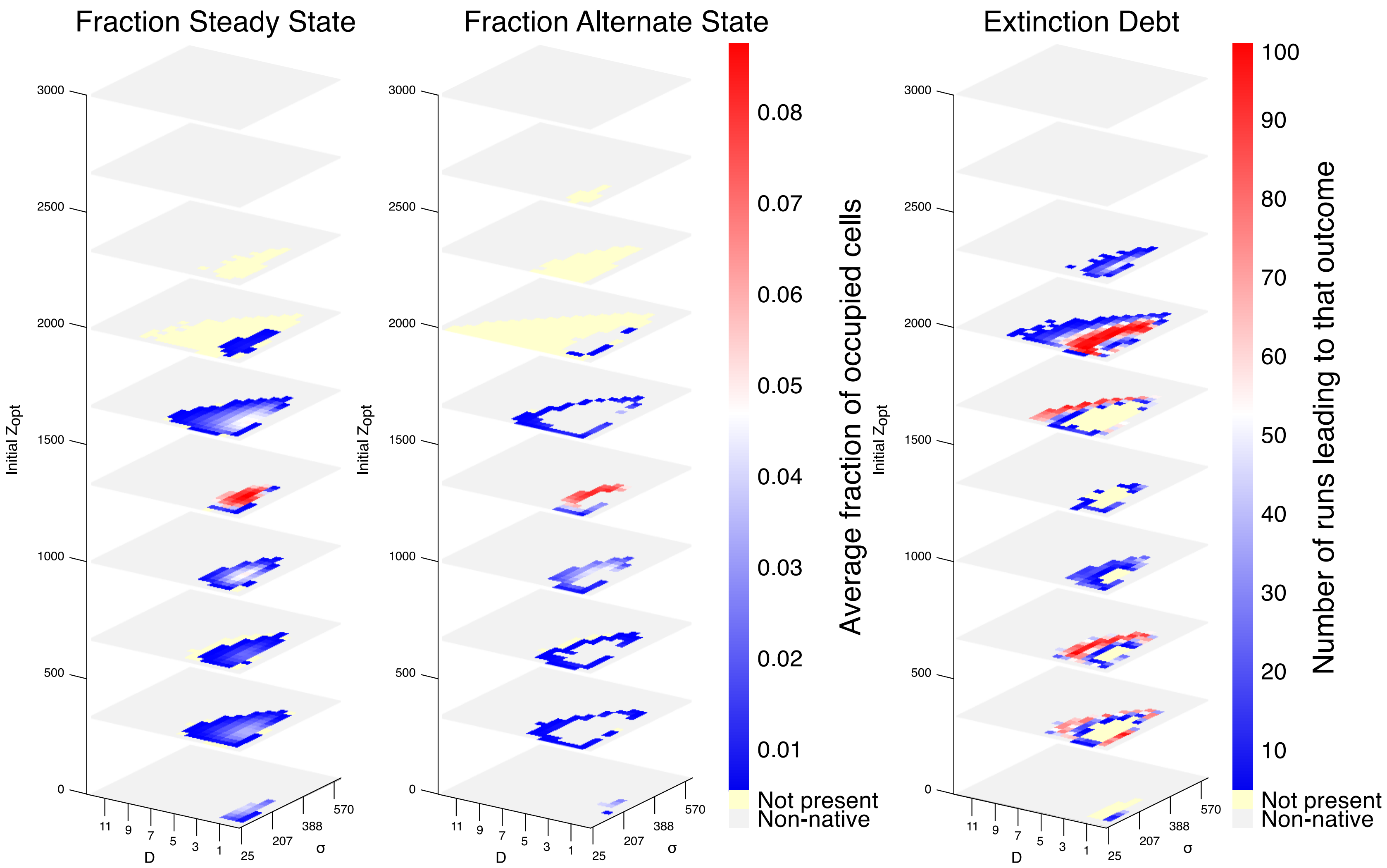

Fig S3.30. Same as figure S3.2 but for the GPNP

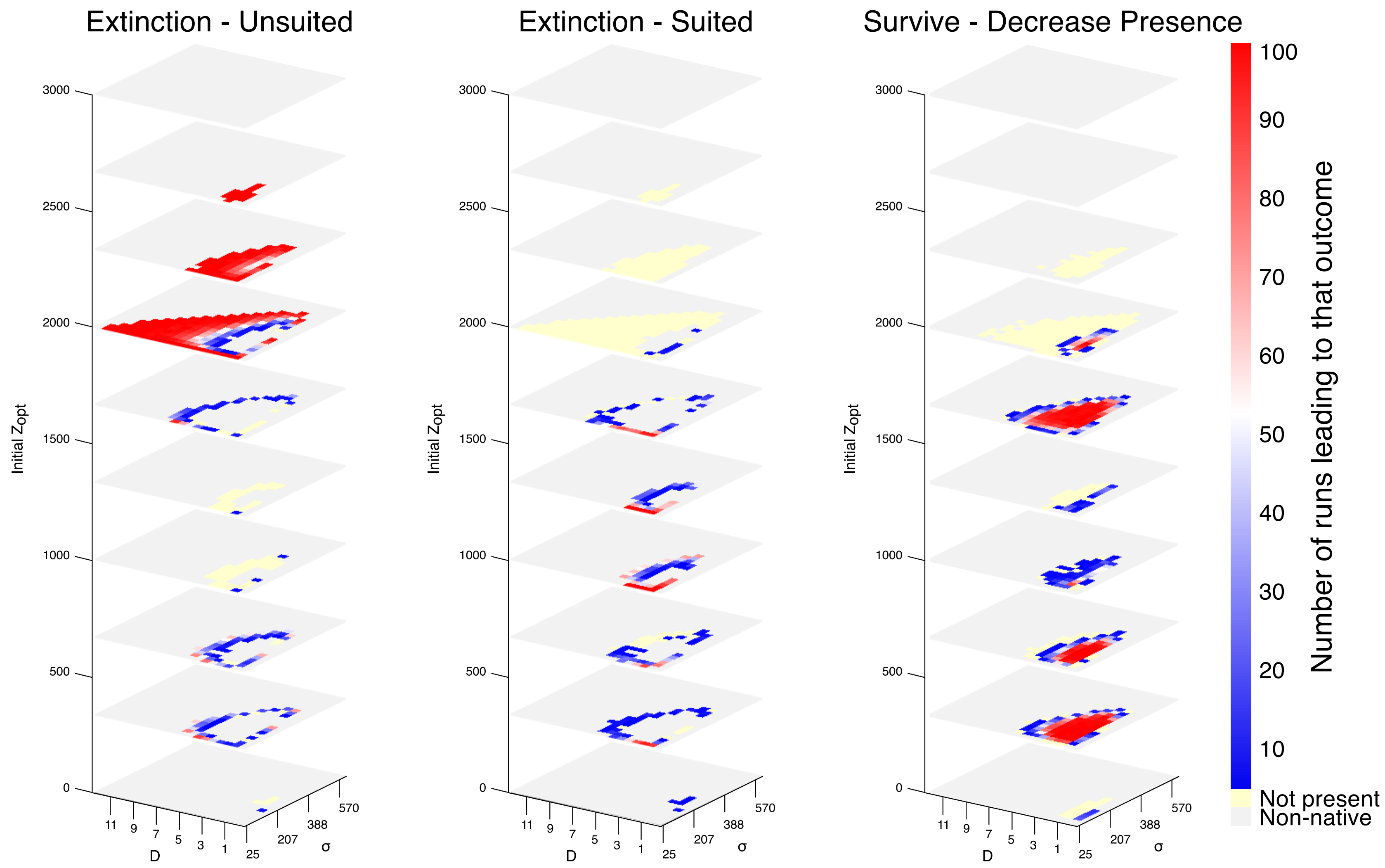

Fig S3.31. Same as figure S3.3 but for the GPNP

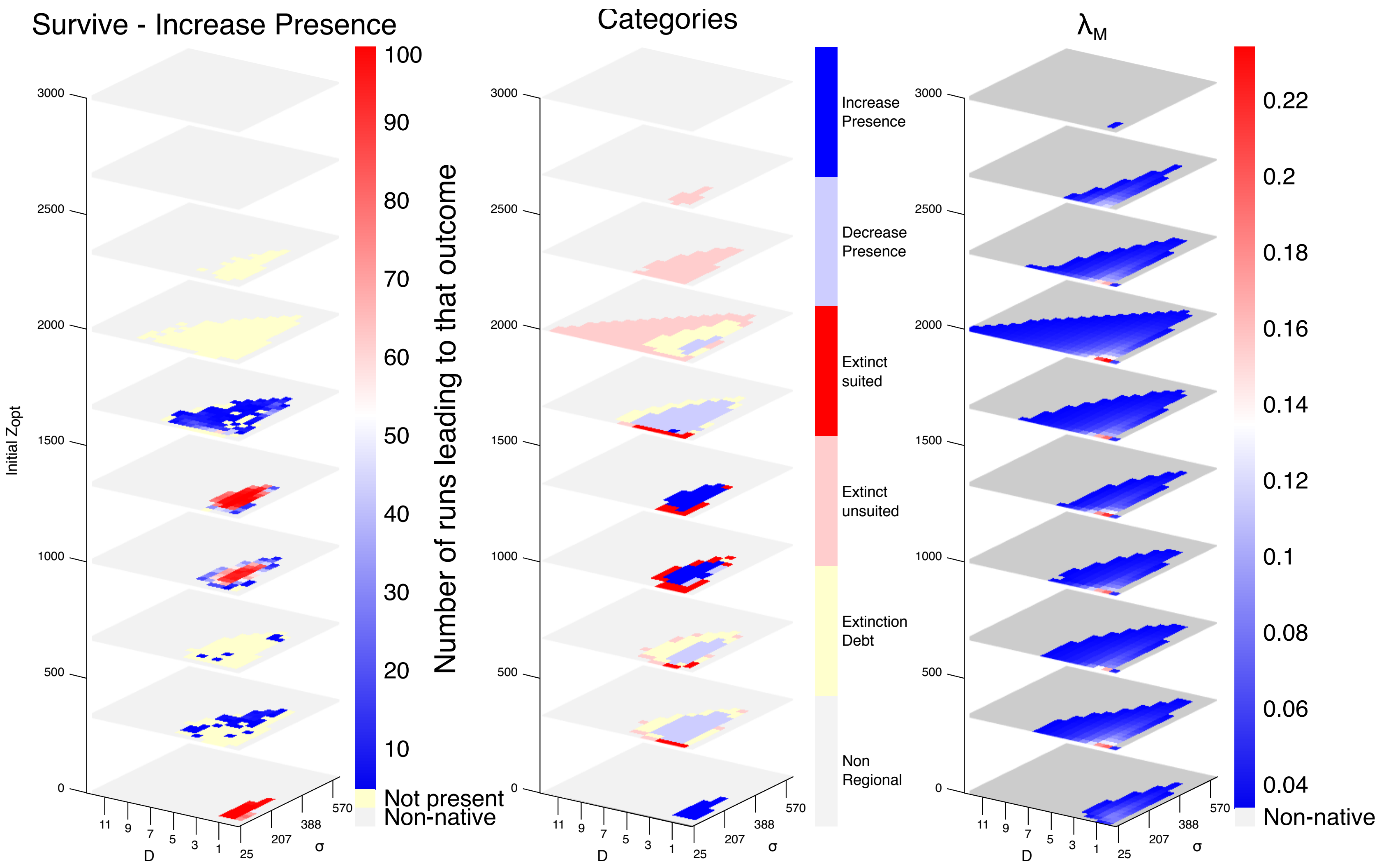

Fig S3.32. Same as figure S3.4 but for the GPNP

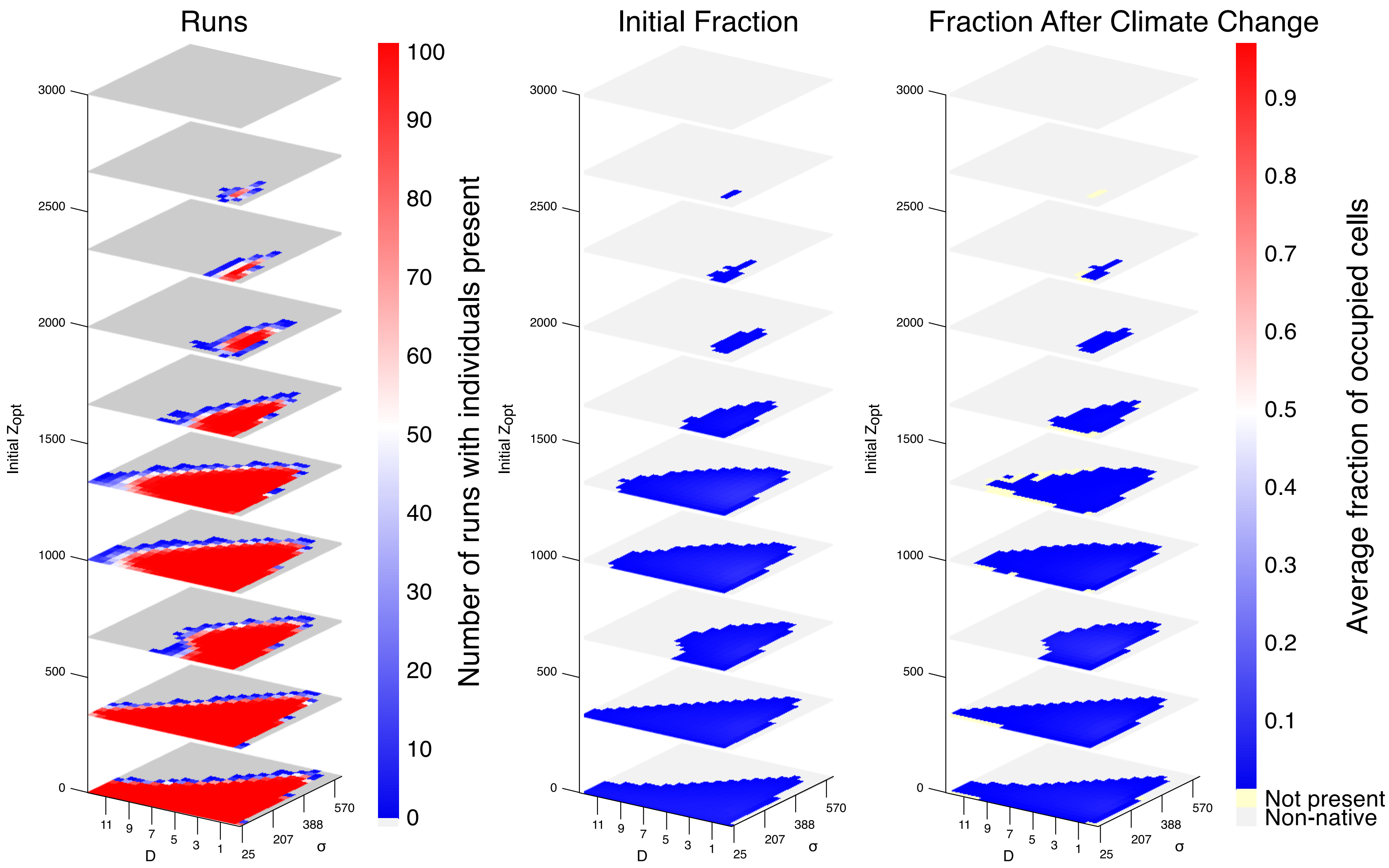

Fig S3.33. Same as figure S3.1 but for the Alpes Vaudoises

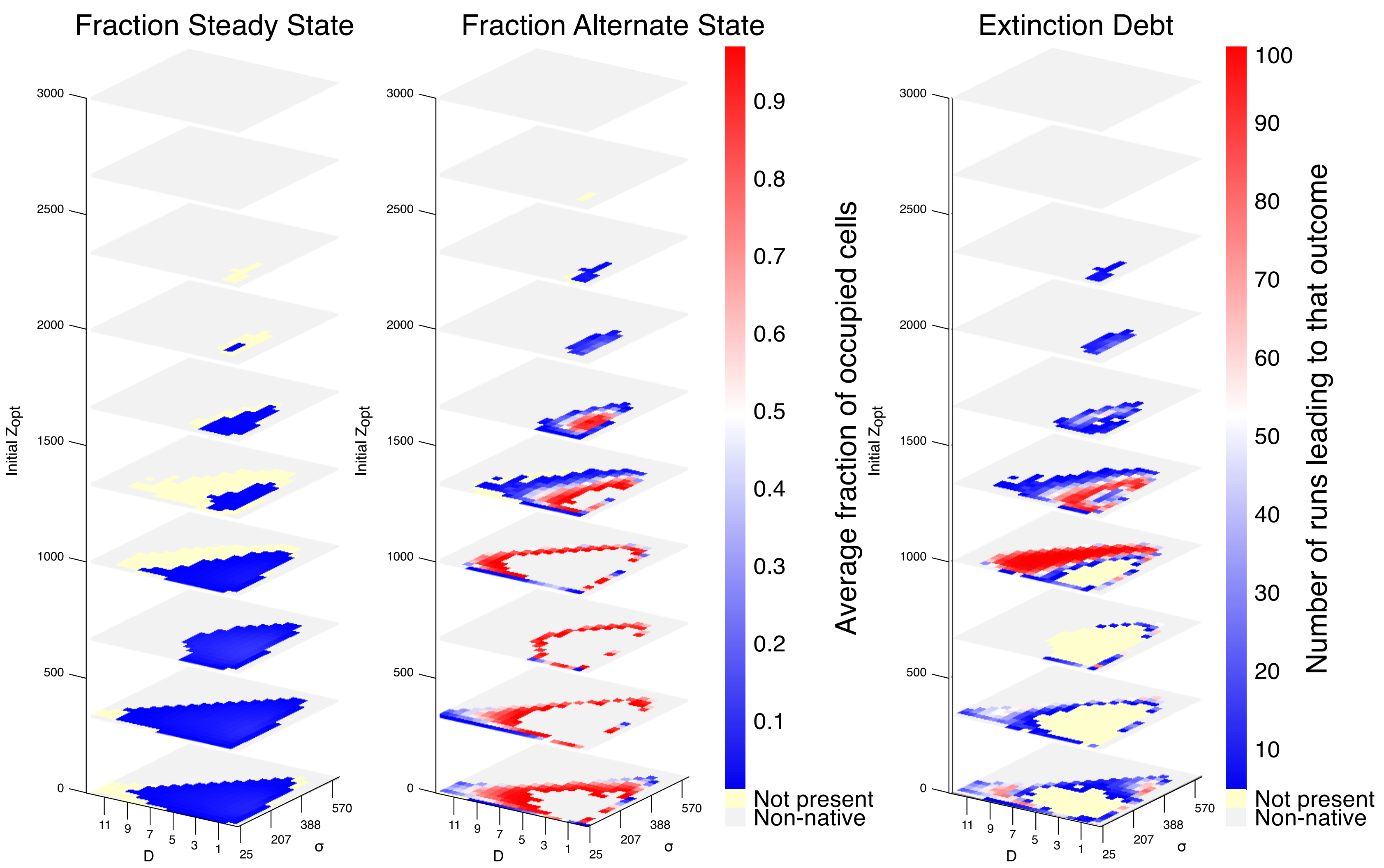

Fig S3.34. Same as figure S3.2 but for the Alpes Vaudoises

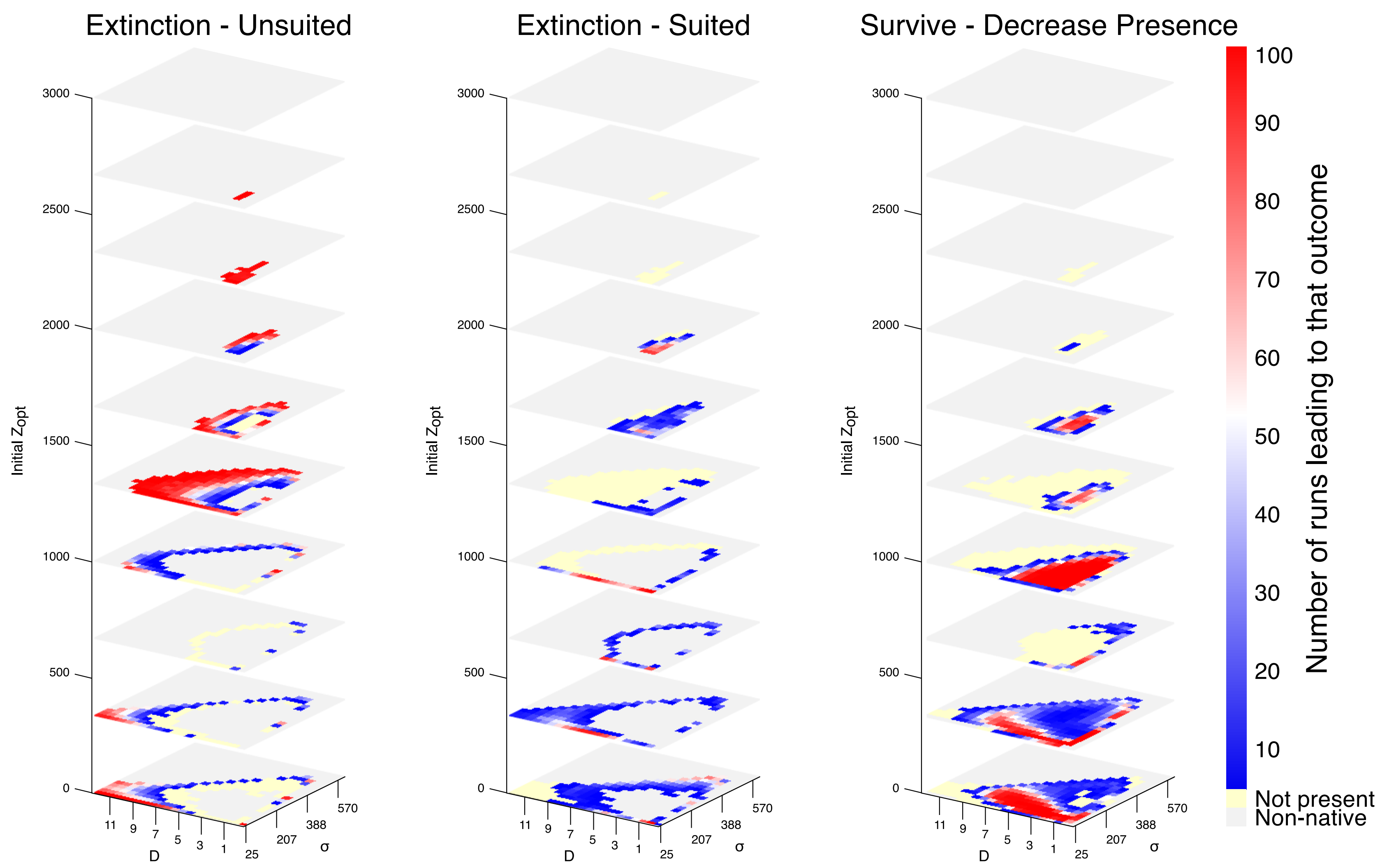

Fig S3.35. Same as figure S3.3 but for the Alpes Vaudoises

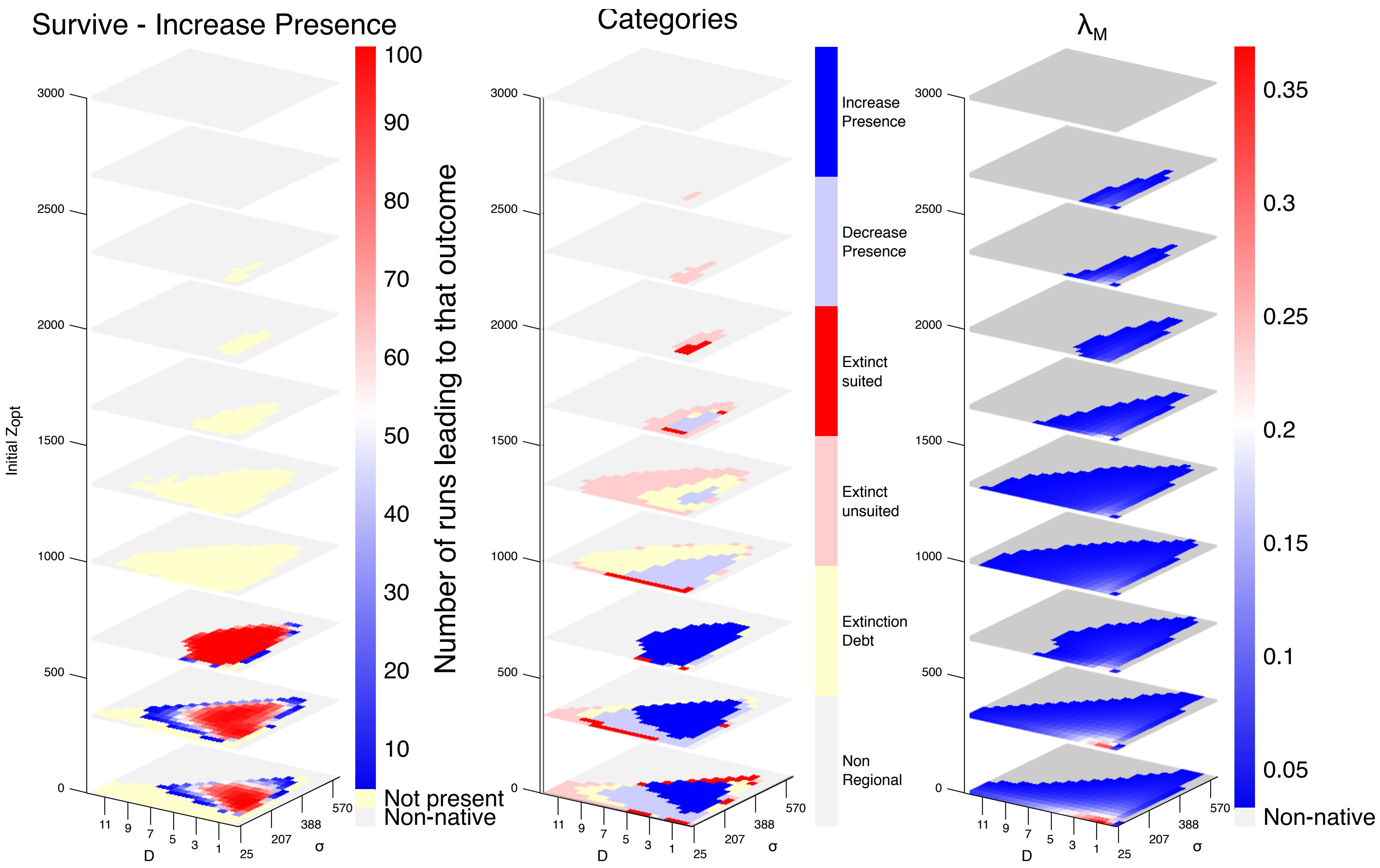

Fig S3.36. Same as figure S3.4 but for the Alpes Vaudoises

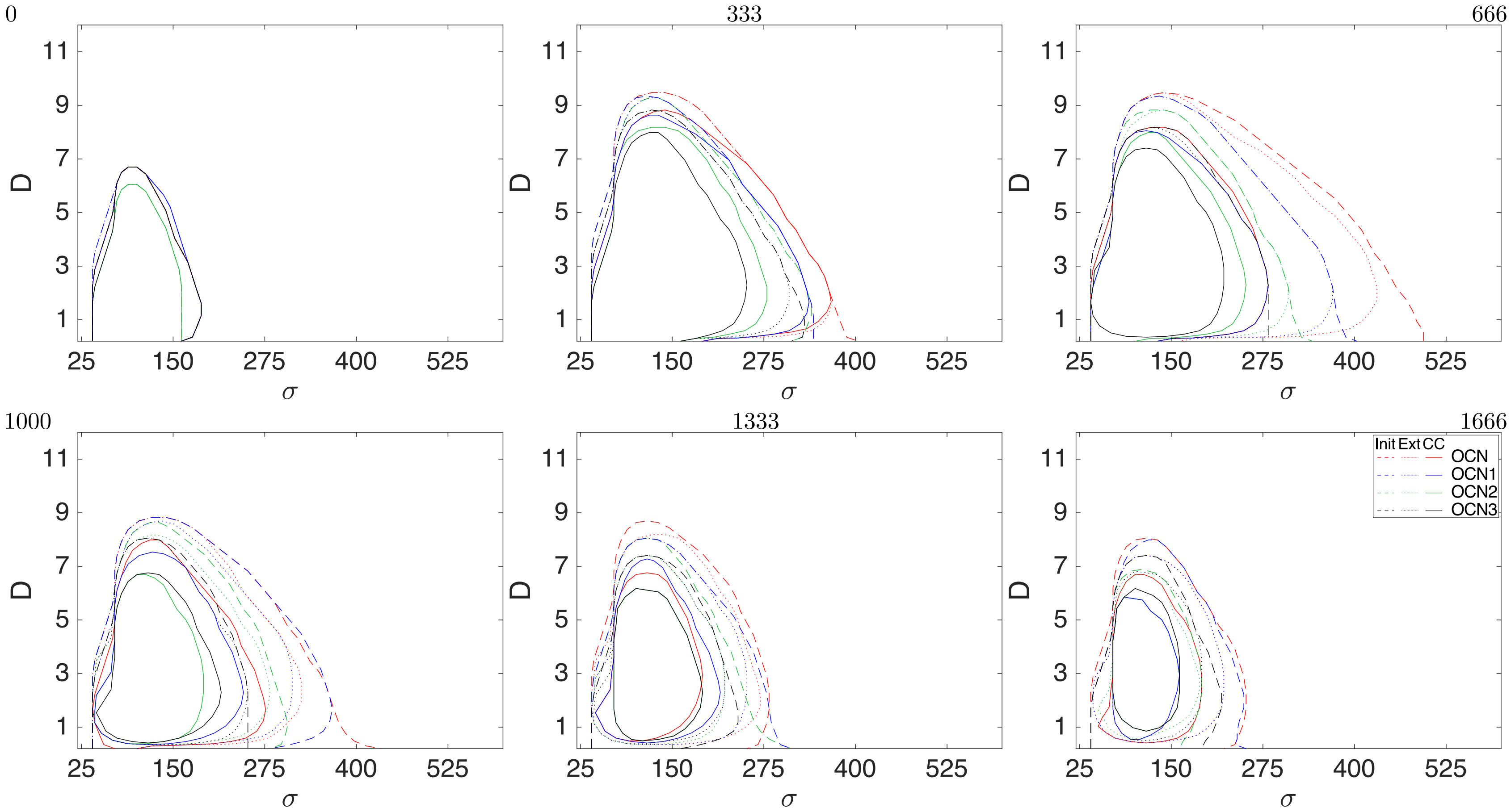

Fig S3.37. As in main text for additional landscapes

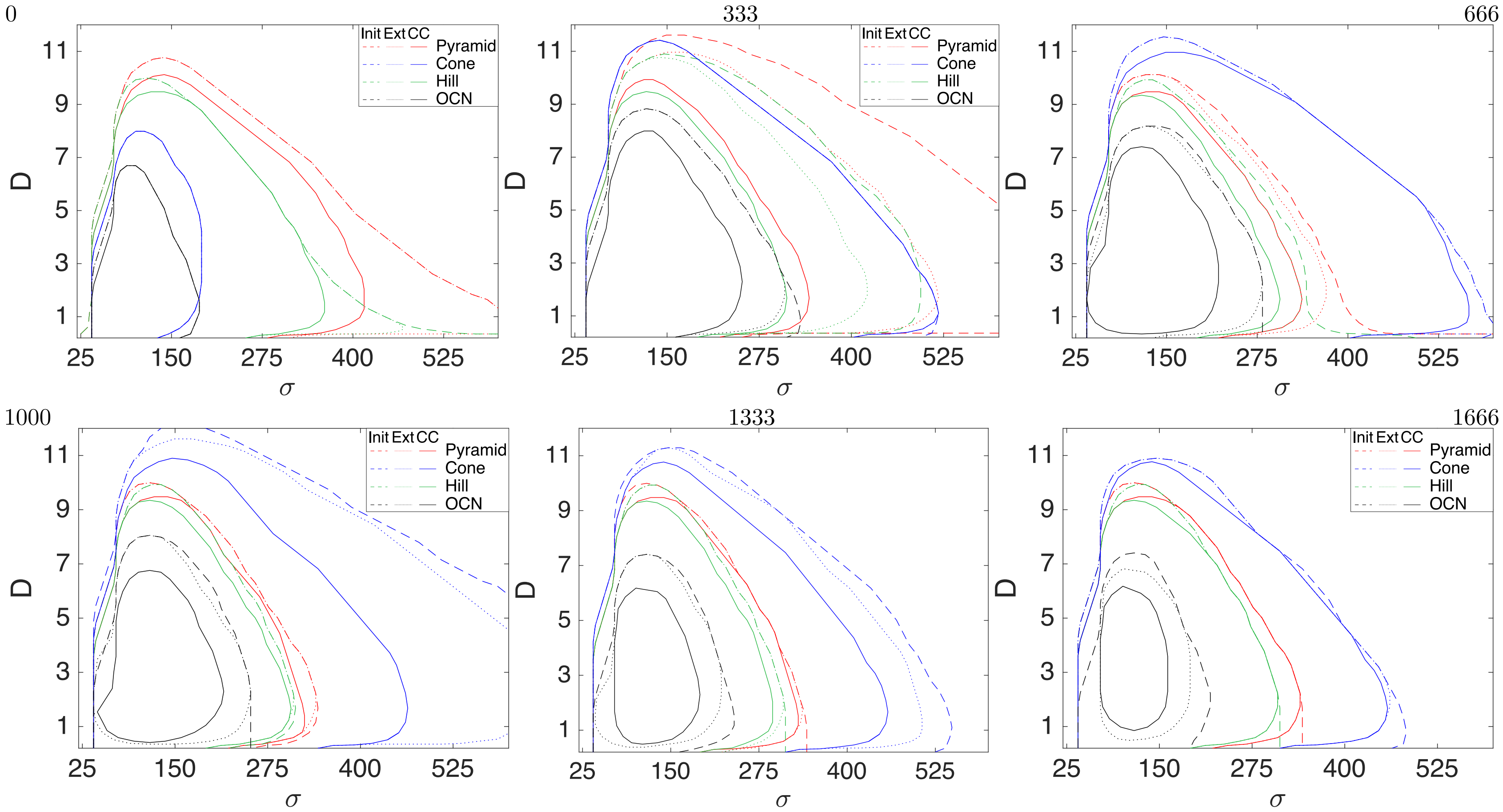

Fig S3.38. As in main text for additional landscapes

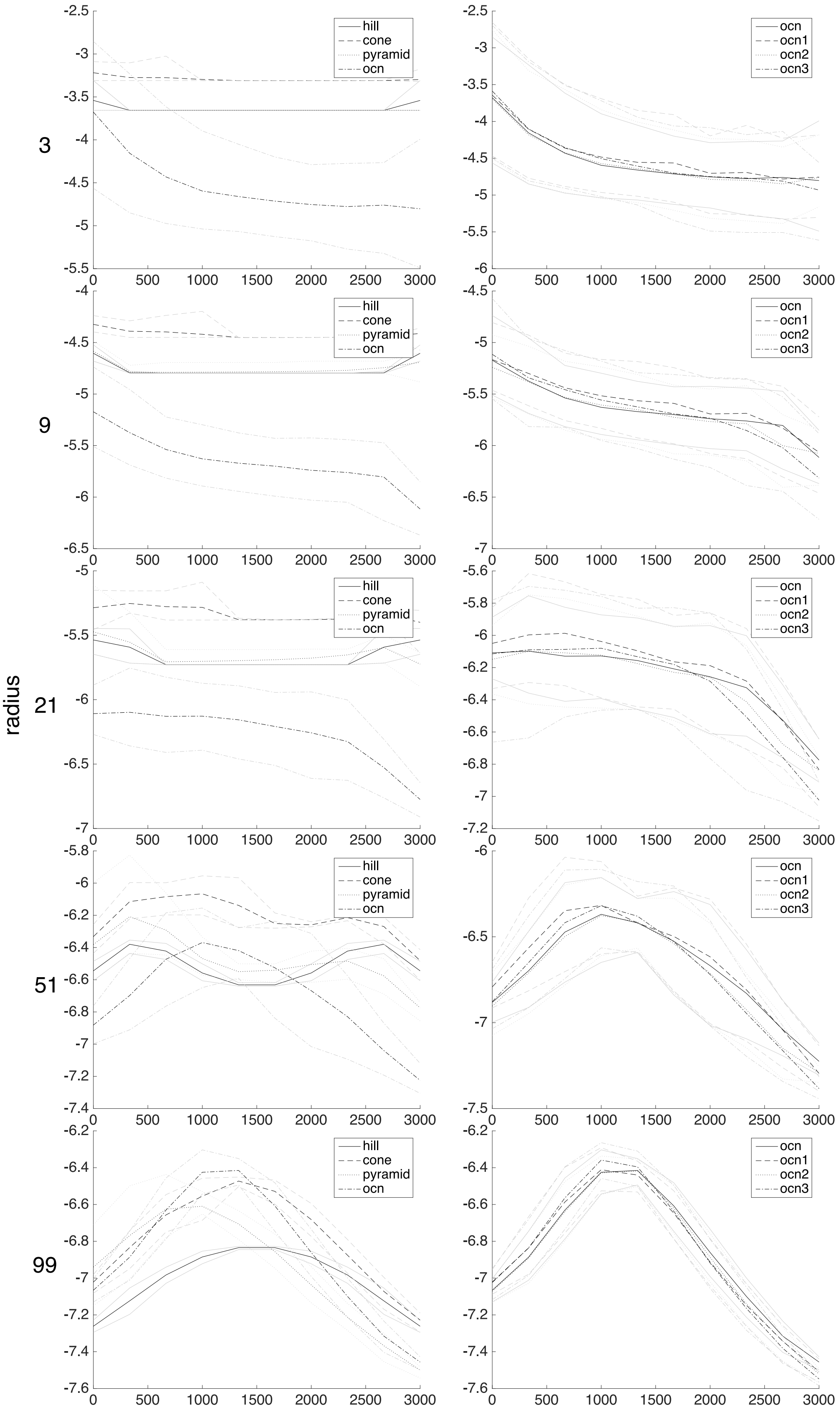

Fig S3.39. Comparison of available area in a given radius around the pixel for the different shapes.

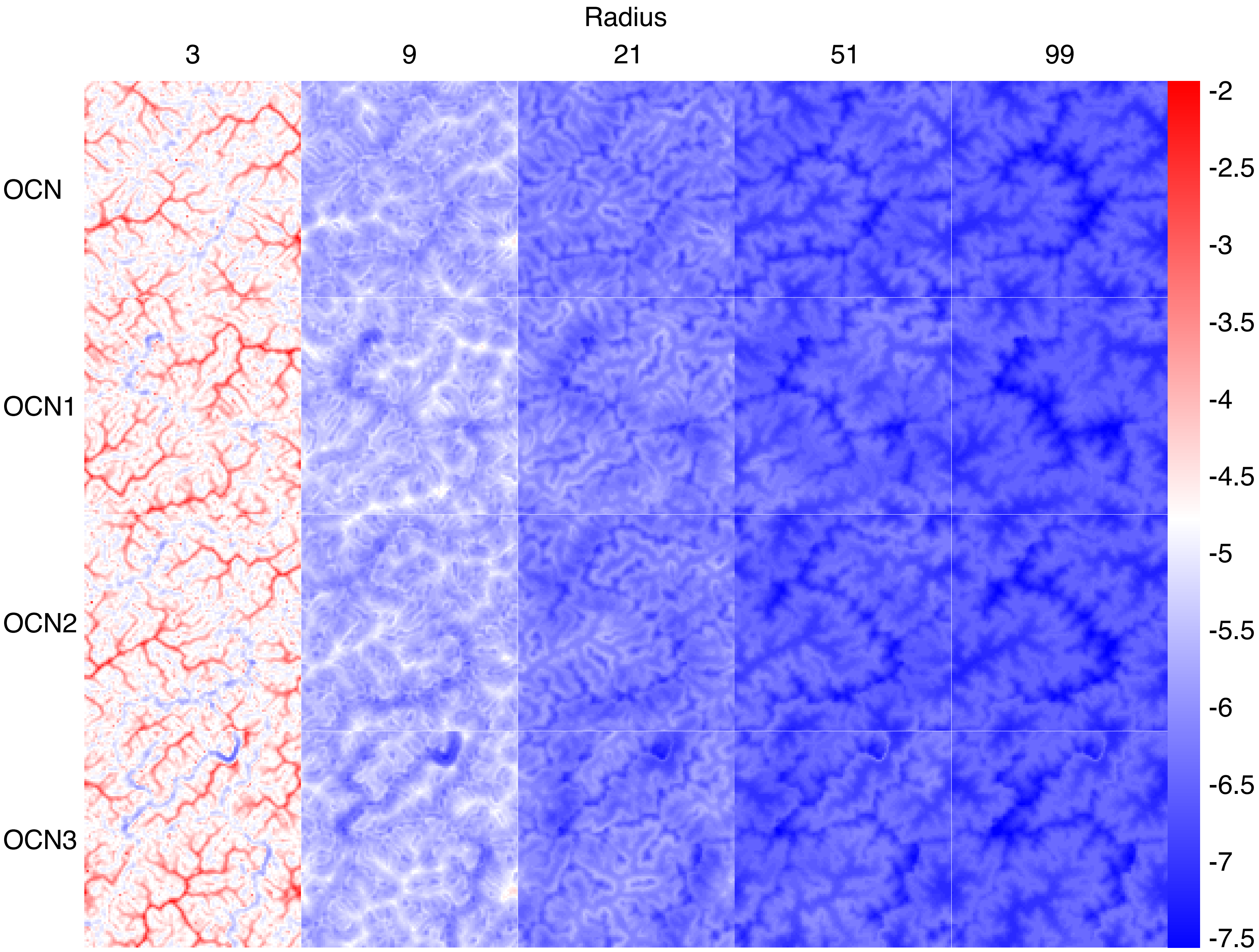

Fig S3.40. Average similar elevation area around each pixel computed as $\overline{z_i} = -\log\left(\sqrt{\frac{\sum_j^N (z_j - z_i)^2}{N}}\right)$, where N represents the number of pixels within a radius r around pixel i.

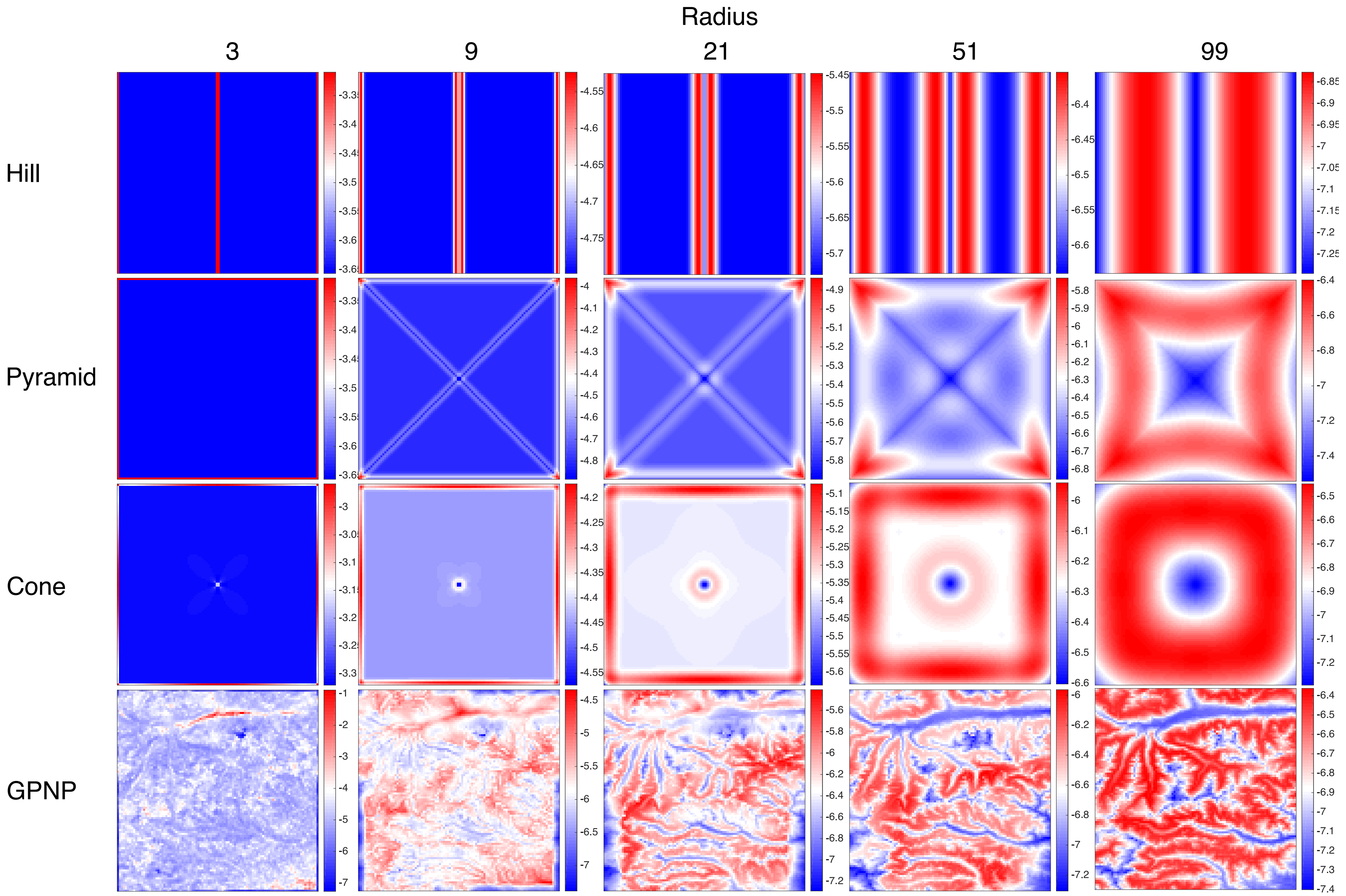

Fig S3.41. As figure S3.40 for the remaining shapes

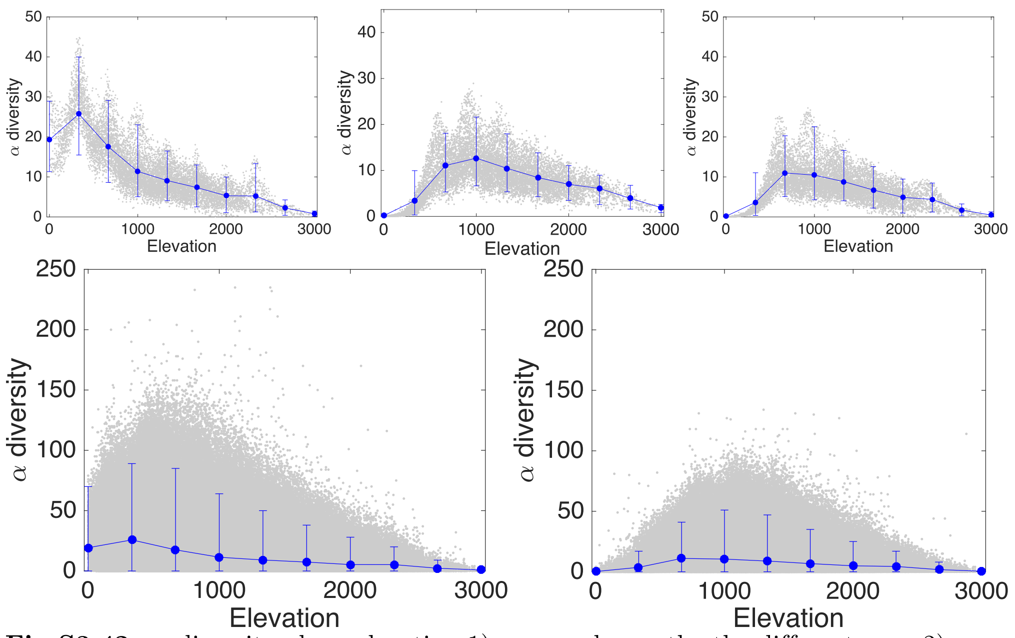

Fig S3.42. α-diversity along elevation 1) averaged over the the different runs 2) raw data

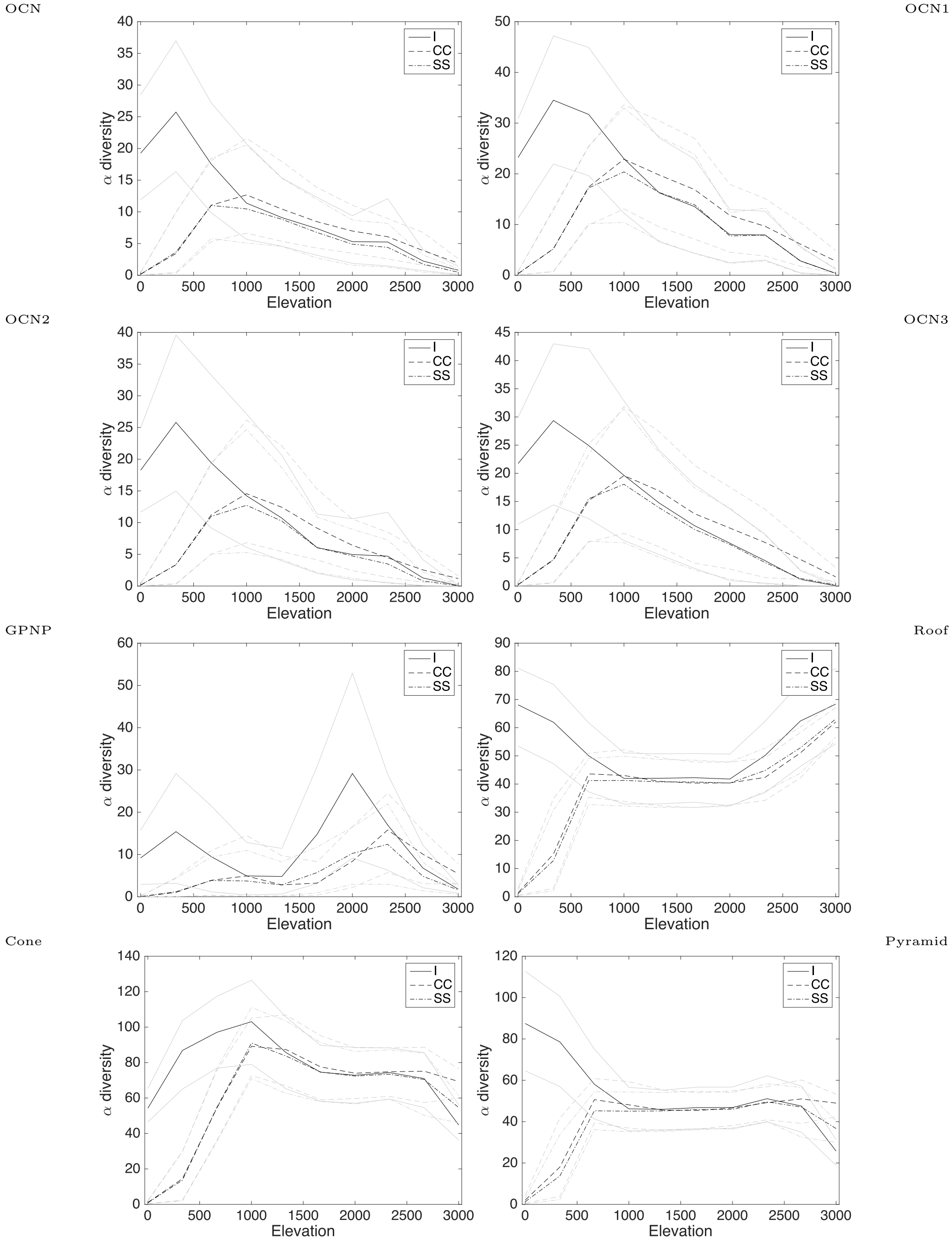

Fig S3.43. Evolution of the α-diversity for the different shapes and the different states - initial (I), after climate change (CC) and at steady state after climate change (SS)

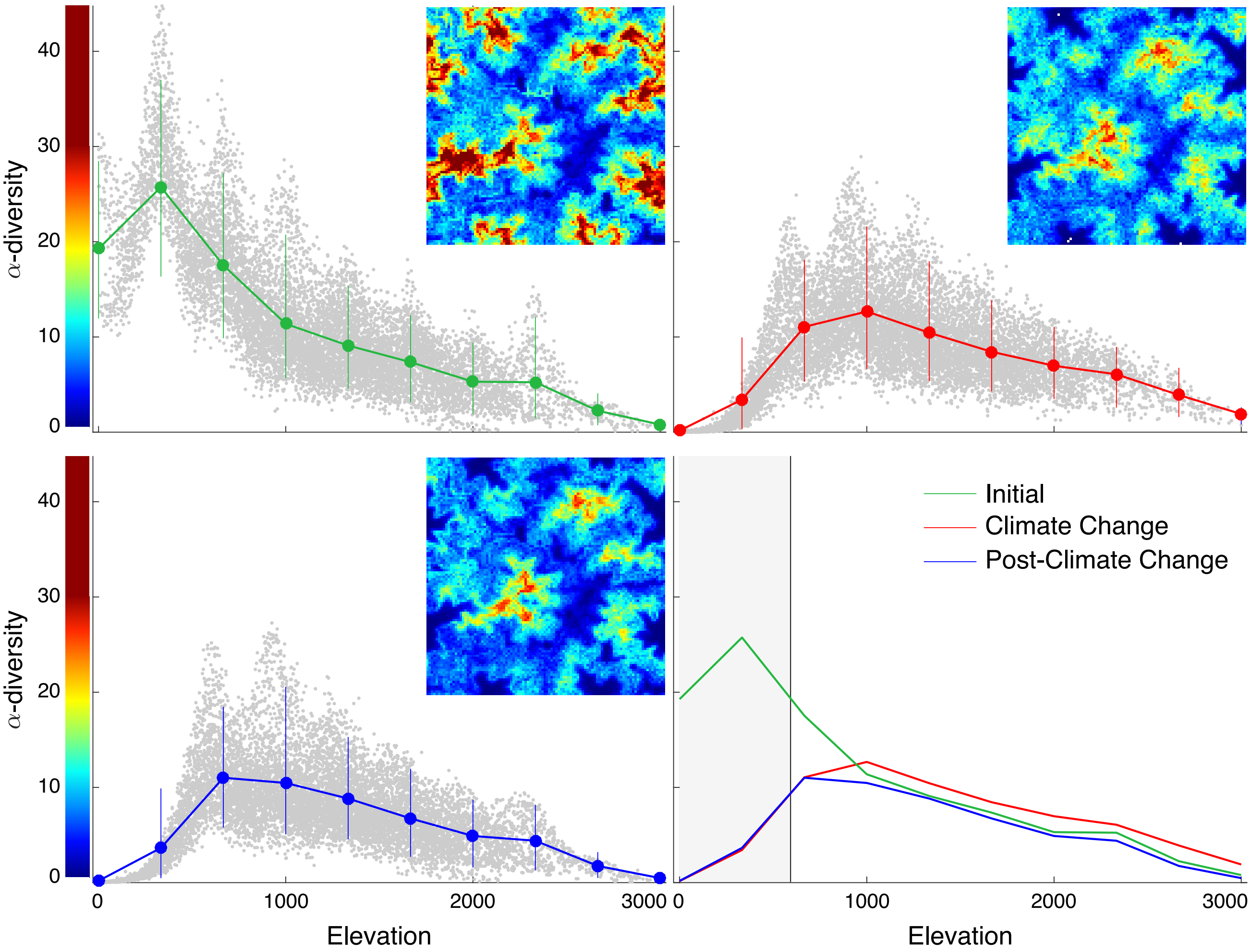

Fig S3.44. α-diversity averaged over 100 runs of the simulation for the OCN. The maps and the scatter plots show the values in the landscape at the initial situation, after climate change and at steady state after climate change. The last plot shows a comparison between the three phases. The grayed-out zone represents the difference in elevation caused by climate change, from the lowest elevation in the area, incidentally defining the part of the study area where caution should be taken in any interpretation, as no new species can colonize from lower elevations outside the area.

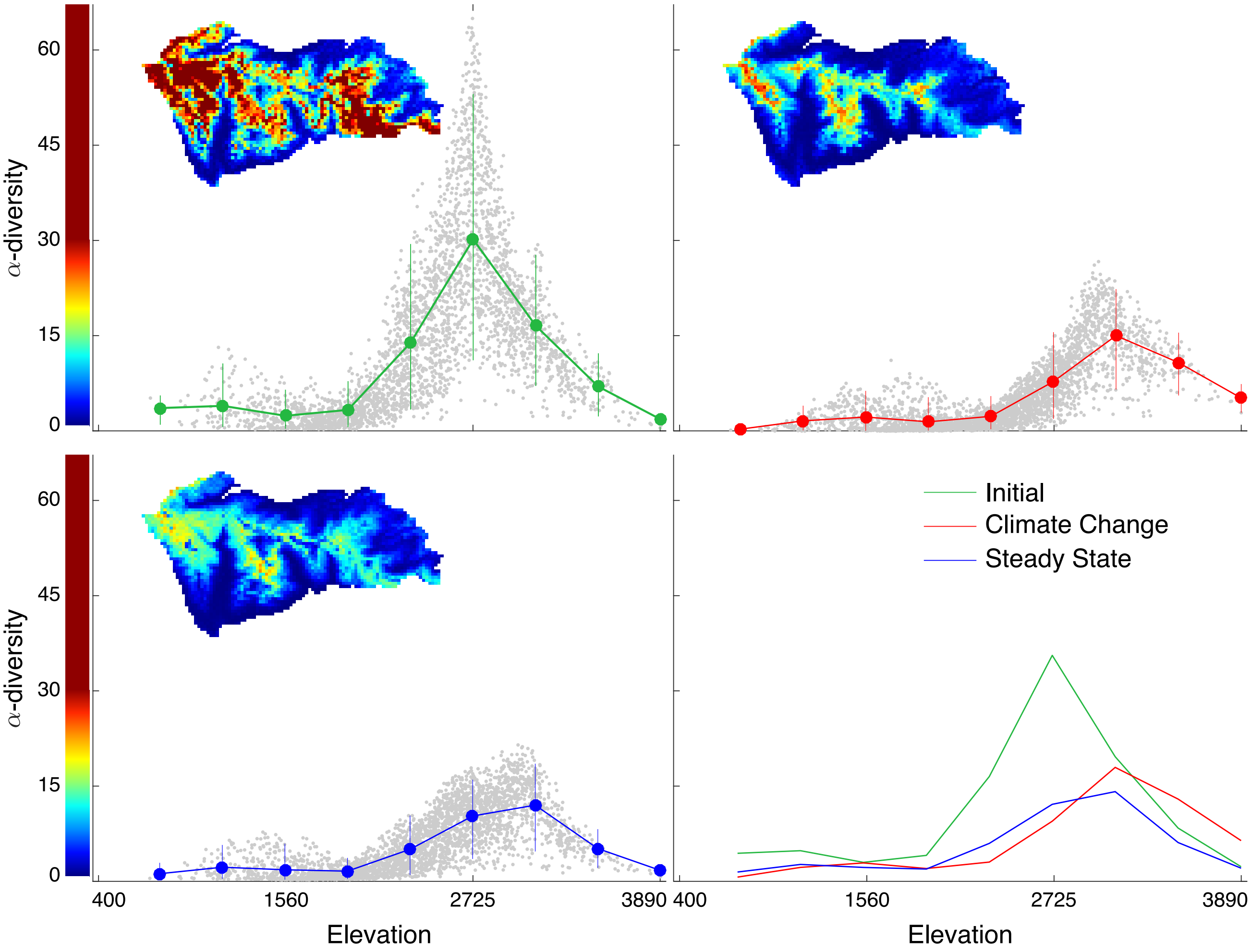

Fig S3.45. α-diversity for GPNP
