# Supplementary material for: A minimalist model of extinction and range dynamics of virtual mountain species driven by warming temperatures
Source: PLoS One. 2019 Mar 18;14(3):e0213775. doi: 10.1371/journal.pone.0213775 (PMC6422262; doi:10.1371/journal.pone.0213775)
Supplement: S4 File — (PDF) [file pone.0213775.s004.pdf]

Appendix S 4
A minimalist model of extinction and range dynamics of mountain species driven by warming temperatures

Jonathan Giezendanner[1*], Enrico Bertuzzo[1,2], Damiano Pasetto[1], Antoine Guisan[3], Andrea Rinaldo[1,4]

1 Laboratory of Ecohydrology, École Polytechnique Fédérale de Lausanne, 1015 Lausanne CH
2 Department of Environmental Sciences, Informatics and Statistics, University Cà Foscari Venice, 30123 Venezia Mestre IT
3 Department of Ecology and Evolution, University of Lausanne, 1015 Lausanne CH
4 Dipartimento ICEA, Università di Padova, 35131 Padova IT

* jonathan.giezendanner@epfl.ch

S 4 Generalized fitness model

S 4.1 Heterogeneous fitness

As stated in the Methods section of the main text, our landscape are highly heterogeneous, with valleys and peaks making up often self-affine topographies [1] defining the connectivity between different areas. Within such types of heterogeneous areas, elevation can be seen as proxy for different local conditions, such as atmospheric pressure, temperature and clear-sky turbidity [2] which vary linearly moving towards mountain tops. These local conditions, among others, influence the distribution and dynamics of mountain species [3]. The distribution of available area at a given elevation often displays a hump at mid-elevation regardless of relief [4], thus influencing the available area for a given species to successfully spread.

Whether building upon an usual grid-like display of DEMs or working through statistical replicas of ONCs, an elevation field z_i. $i = 1, \ldots, N$ spanning all N landscape sites is assigned. In the general case, we assume strictly altitude-dependent fitness. Thus, we propose that a given species response to a certain elevation z_i is given as a fitness field f_i (as assumed elsewhere [5, 6]):

$$f_i = \sqrt{\frac{1}{\sigma}} \exp\left(-\frac{(z_i - z_{\text{opt}})^2}{2\sigma^2}\right)$$

where z_{opt} describes the elevation at which the species shows its maximal fitness, σ the niche width defining the elevation band in which the species can possibly thrive.

Once assigned the two parameters z_{opt} and σ, the heterogeneity of the landscape derives the spatially explicit fitness for each species. The model then proceeds as described therein, noting that E and C are the extinction and colonization rates respectively, Δt is the simulation time step, and p the binary state of occupancy of cell i, either 1 for occupied, or 0 for empty. Note that the colonization and extinction mechanisms are directly related to the fitness (equation 3 of the main text). The

extinction rate is inversely proportional to the fitness of the species to the cell, i.e. the lower the fitness, the higher the chance of going extinct in the given cell:

$$E_i \;=\; e/f_i$$

where e is the extinction constant. The colonization rate of an unoccupied cell is driven by the sum of the pressures from surrounding occupied cells, defined by an exponential kernel multiplied by the fitness associated to the source cells, i.e. $C_i = \sum_{j\neq i} p_j \frac{e^{-d_{i,j}/D}}{2\pi D^2} f_j$ with distance d between cells i and j and average colonization distance (or dispersal) D. Alternatively, a more general formulation could have used $C_i = \sum_{j\neq i} p_j \mathcal{K}(i,j)\, f_j$ where $\mathcal{K}(i,j)$ is a general dispersion function between cell i and j, not necessarily exponential. The coefficients of extinction e and colonization c are maintained constant as $e = 0.02$ and $c = 15$.

In the Methods section of the main text, we have noted that our framework corresponds to a basic modification of the concept of metapopulation capacity [7], in that our landscape matrix $\mathbf{M}$ consists of elements m_{ij} such that:

$$m_{ij} = \frac{e^{-d_{i,j}/D}}{2\pi D^2}\; f_i\; f_j \qquad \text{and} \qquad m_{ii} = 0. \tag{1}$$

Thus, *sensu* Hanski, the maximum eigenvalue of the modified landscape matrix $\mathbf{M}$, λ_M, gives the conditions for persistence of a species in a given landscape ($\lambda_M > e/c$) where fitness, in this case, depends strictly on elevation. Because $\mathbf{M}$ is a nonnegative square and irreducible matrix, according to the Perron-Frobenius theorem it has a positive and simple maximum eigenvalue λ_M and a unique positive eigenvector associated to it. To compute λ_M for a given landscape, what matters is just the spatial scale of connectivity defined by the average dispersal distance D and the spatial locations of the habitat patches, here identified as any pixel of elevation z_i and fitness f_i for the given landscape. For a given species, λ_M is a measure of the ecological suitability of the landscape and it has powerful implications as it allows comparisons of differently connected landscapes where the relative contribution of any site to all others is accounted through d_{ij}, i.e., the distances available to ecological dispersal. Moreover, it has been shown [7] that an appropriately weighted average of the equilibrium occupancy probability values p_i^* can be approximated by $p_i^* \sim 1 - e/(c\lambda_M)$. Therefore, when the conditions for persistence are satisfied, the higher the metapopulation capacity, the higher the expected occupancy of the population. Metapopulation capacity can be used specifically to rank different landscapes in terms of their capacity to support viable metapopulations. We shall employ such concept to analyze how different landscapes fare in their capacity to support long-term persistence of arbitrary metapopulations. It is then possible to derive the contribution of the ith pixel to λ_m by computing the corresponding eigenvector:

$$\lambda_i \equiv x_i^2 \lambda_M \tag{2}$$

where x_i is the eigenvector's i^{th} entry. In the context of the paper, this permits to understand the areas of suitability of a species defined by a combination of the parameters dispersal D, niche width σ and optimal elevation z_{opt}, the latters being used in the definition of f_i. Such a condition can be relaxed, however, to make fitness landscapes more realistic and capable of contrasting real data, e.g. including climatic and topographic predictors like degree-days above 3°, suitable moisture indices, daily average global potential shortwave radiation per month or annual average number of frost days during the growing season for plants [8]. Fitness f_i may easily be made dependent also on elevation-independent factors (SI). It is then possible to derive the contribution of the i-th pixel (λ_i) to the metapopulation capacity λ_M of the landscape by computing the eigenvector x_i corresponding to the maximum eigenvalue of m_{ij} and by projecting x_i to yield [7] $\lambda_i = x_i^2 \lambda_M$. The probability of occurrence of actual species in real landscapes could therefore be mapped by our fitness model.

S 4.2 Comparative studies

Thibaud et al. [8] have measured the relative effect of factors affecting species distribution model predictions and mapped the probability of occupancy in the Vaud Alpes for different species. They employed a statistical framework using spatial autocorrelation with respect to five climatic and topographic predictors.

Using the method described earlier as a proof of concept, their results can be qualitatively approximated (Fig. S4.1).

While obviously no serious attempt at calibrating parameters has been made at this stage, the general patterns observed in Thibaud et al. [8] seem to be relatively well reproduced by the eigenvector projection method when cherrypicking the right parameter set.

We deem the above result remarkable and worth future investigations, as demonstrated by the following exercise. In fact, the strength of the metapopulation capacity approach, modified to embed heterogeneous local fitnesses, is that f_i can be suitably made a function of any relevant factor of choice. For instance, following *ex post* the suggestion of Thibaud et al. [8], one could take into account factors derived from any GIS technique or database like e.g.: degree-days above +3°C, a moisture index between June and August, daily average potential short-wave radiation per month, annual average number of frost days during the growing season and an index of topographic position. Here, as a proof or concept, we assume that only aspect is taken into account to define the metapopulation fitness. Specifically, the fitness is then modified in the following way:

$$f_i = \sqrt{\frac{1}{\sigma}} \exp\left(-\frac{(z_i - z_{\text{opt}})^2}{2\sigma^2}\right) \cdot \phi(\alpha) \tag{3}$$

where α is the aspect, and $\phi(\alpha)$ a function of the aspect, chosen here in this way:

$$\phi(\alpha) = a + \frac{\sin(\alpha + b) + 1}{2} \cdot (1 - a) \tag{4}$$

where a defines the minimum modifier value, and b the angle with maximal fitness. Figure S4.2 shows the same four species as shown before, but with the additional aspect modifier (with the max fitness being fixed when south-facing and a constrained minimal modifier of 0 and 0.5). The northern and southern valleys are more easily distinguished by the projection of the eigenvector, but overall it seems that the elevation already explains most of the observed patterns, leading to the suggestion that beyond a certain scale, only the elevation field remains the key determinant, as noted earlier [9]. We thus conclude that the present method can be made compatible with the procedure of Thibaud et al. [8] while retaining the predictive character implicit in the metapopulation model. A detailed comparative study will be the subject of a forthcoming paper. For the goals of the present paper, centered as it is on a predictive framework to discuss how climate change might impact biodiversity in mountain environments, suffice here to note that the landscape matrix approach allows a novel perspective, akin to scenarios of temperature rise objectively treated.

S 4.3 Random Noise Field

The principal aim in this paper was to single out the different effects of geomorphology on metapopulation dynamics, principal reason for the sole use of elevation as fitness in the main paper. Of course, in nature, species are being affected by more than simply elevation. To simulate the effect of other factors on the species fitness, a random noise filed was used to perturb the elevation-driven fitness. The random fields were generated

using HYDRO_GEN [10] (Fig. S4.3), with the integral scale between 1 and 50 (half of the field size), and variance between 0.1 and 1. The mean remained at 1 for all randomly generated fields. For each species, a random field was generated, and the fitness modified:

$$f_i' = X_i \cdot f_i \cdot \frac{\sum_i f_i}{\sum_i f_i \cdot X_i} \quad (5)$$

such that the sum of the fitness over the landscape was preserved. X_i represents here the value of the random field at pixel i.

Figure S4.3 shows the difference between fates of species before, and after the addition of noise to the elevation field, for the OCN, Alpes Vaudoises and GPNP landscapes. The added noise field reduces the effect of the niche width, while at the same time highlighting the effect of dispersal, with a clear gradient of 'surviving' → 'extinction debt' → 'extinction' when going from small to large niche width in the upper parts of optimal elevation.

These results suggest that the observations made in the main text are indeed to be related to geomorphic effects.

References

1. Rodríguez-Iturbe I, Rinaldo A. Fractal river basins: chance and self-organization. Cambridge University Press; 2001.

2. Körner C. The use of 'altitude' in ecological research. Trends in Ecology & Evolution. 2007;22(11):569–574.

3. McCain CM, Grytnes JA. Elevational Gradients in Species Richness. Encylcopedia of Life Sciences. 2010;15:1–10.

4. Elsen PR, Tingley MW. Global mountain topography and the fate of montane species under climate change. Nature Climate Change. 2015;5:5–10.

5. Rybicki J, Hanski I. Species-area relationships and extinctions caused by habitat loss and fragmentation. Ecology Letters. 2013;16:27–38.

6. Bertuzzo E, Carrara F, Mari L, Altermatt F, Rodriguez-Iturbe I, Rinaldo A. Geomorphic controls on elevational gradients of species richness. Proceedings of the National Academy of Sciences of the United States of America. 2016;113(7):1737–1742.

7. Hanski I, Ovaskainen O. The metapopulation capacity of a fragmented landscape. Nature. 2000;404:755–758.

8. Thibaud E, Petitpierre B, Broennimann O, Davison AC, Guisan A. Measuring the relative effect of factors affecting species distribution model predictions. Methods in Ecology and Evolution. 2014;5(9):947–955.

9. Pradervand JN, Dubuis A, Pellissier L, Guisan A, Randin C. Very high resolution environmental predictors in species distribution models: Moving beyond topography? Progress in Physical Geography. 2014;38(1):79–96.

10. Bellin A, Rubin Y. HYDRO_GEN: A spatially distributed random field generator for correlated properties. Stochastic Hydrology and Hydraulics. 1996;10:253–278.

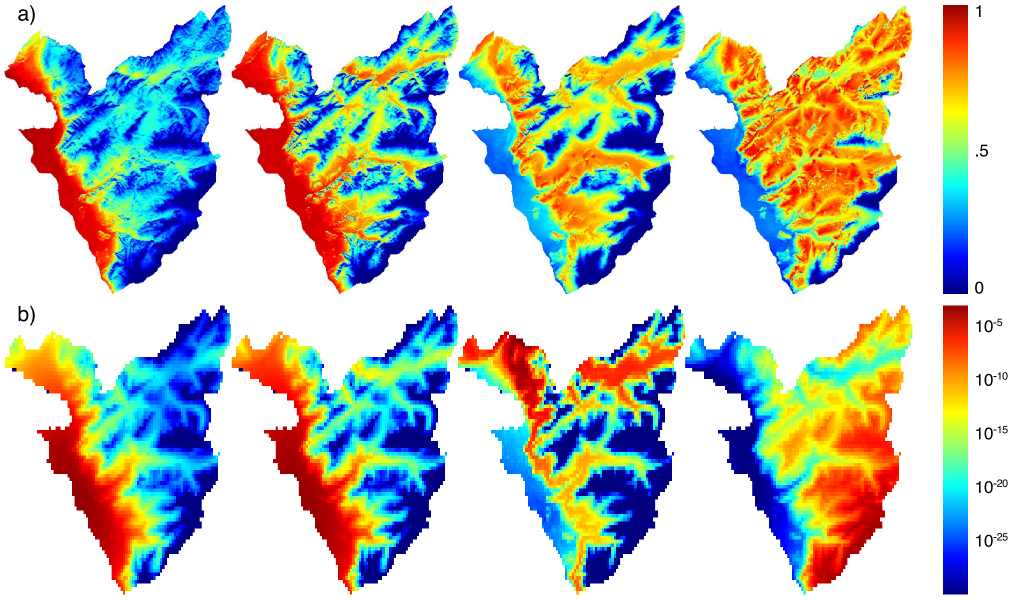

Fig S4.1. Four of the species computed by Thibaud et al. (a) and the visual approximation obtained with the landscape capacity method (log-value) (b). The parameters found to best suit the original images are as follow (σ - D - z_{opt}): (300 - 2.3 - 0), (240 - 4.2 - 0), (115 - 2.9 - 666), (297 - 4.2 - 2000)

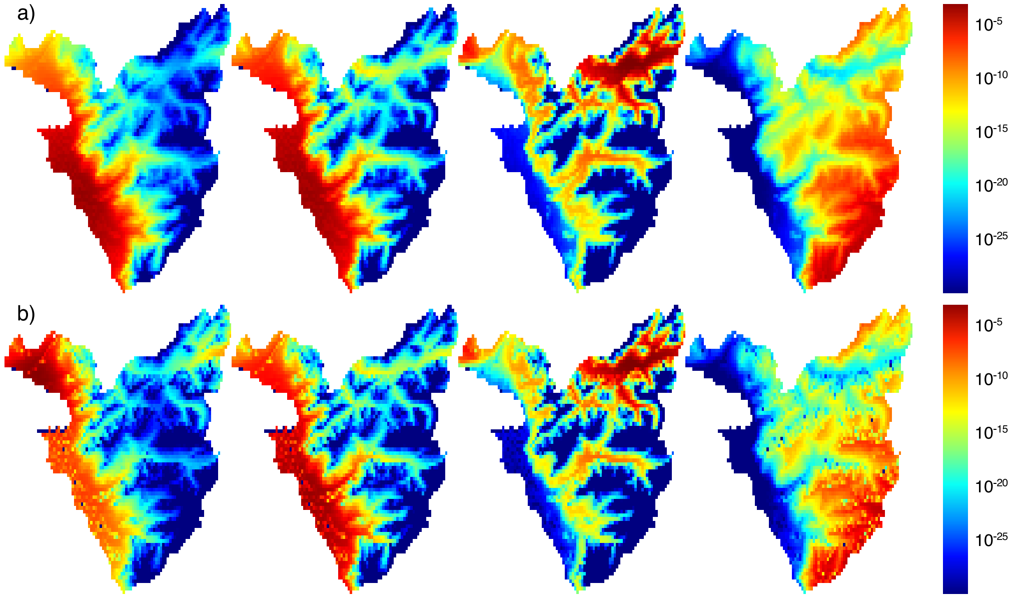

Fig S4.2. The four species selected, with the aspect modifier included (a): $a = 0.5$, b): $a = 0$)

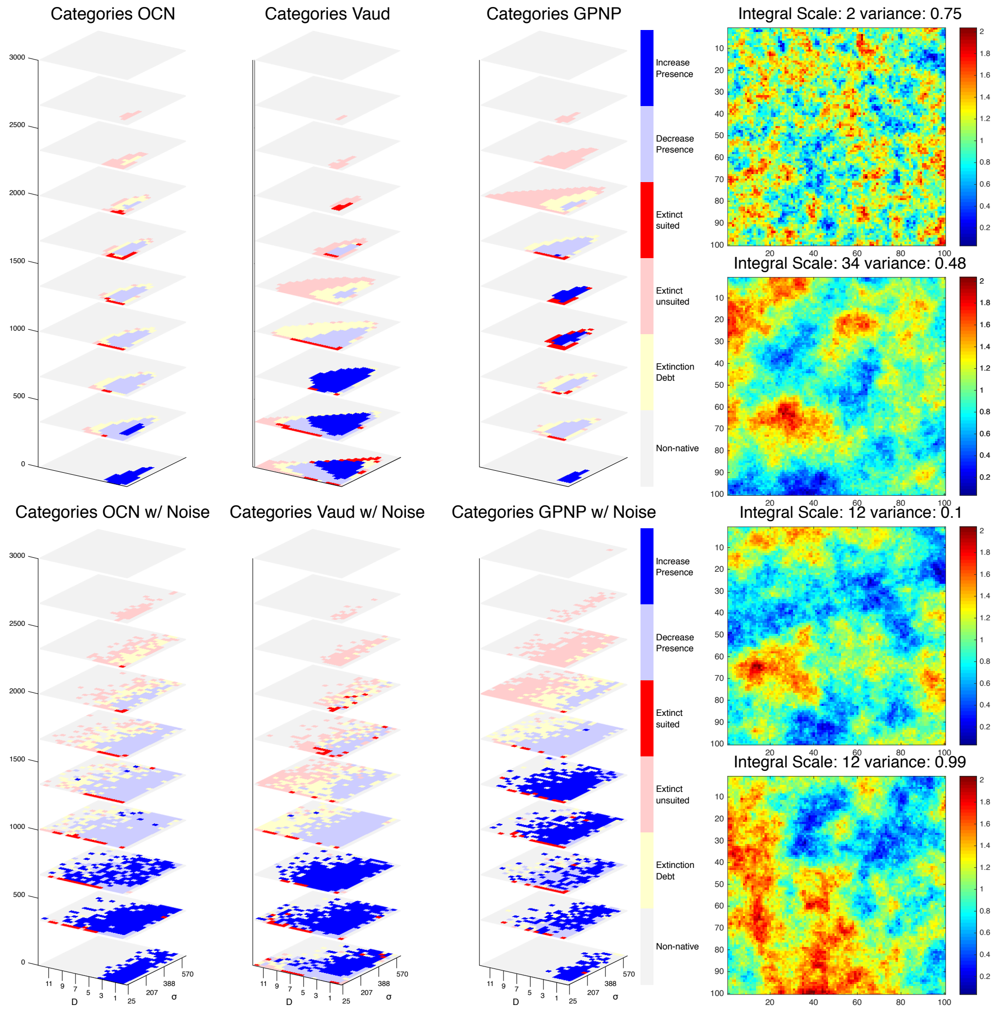

Fig S4.3. Comparison between using only elevation as fitness and adding noise while conserving summed fitness for the OCN, Vaud Alpes and GPNP. The noise fields (sample on the right) were generated using `HYDRO_GEN` [10], with integral scale and variance randomly selected for each species.
